# Inhibitory Effects of *Mangifera indica* Secondary Metabolites and Their Synthetic Derivatives against SARS-CoV-2 M^pro^ and NS2B/NS3 (ZIKV and DENV-2)

**DOI:** 10.1021/acsomega.4c07148

**Published:** 2024-10-24

**Authors:** Gabriella B. Souza, Carime L. M. Pontes, Geovanna de O. Costa, Natália F. de Sousa, Tiago Tizziani, Luiz Antonio E. Pollo, Bibiana P. Dambrós, Marcus T. Scotti, Mario Steindel, Antonio L. Braga, Tanja Schirmeister, Francisco F. de Assis, Louis P. Sandjo

**Affiliations:** † Programa de Pós-graduação em Química, Department of Chemistry, CFM, Universidade Federal de Santa Catarina, 88040-900 Florianópolis, SC, Brazil; ‡ Johannes Gutenberg Universität Mainz, Institute of Pharmacy and Biochemistry, Staudingerweg 5, Mainz DE-55128, Germany; § Chemistry Department, Exact and Nature Sciences Center, Federal University of Paraiba, Campus I, 58051-900 João Pessoa, PB, Brazil; ∥ Department of Pharmaceutical Sciences, CCS, Universidade Federal de Santa Catarina, 88040-900 Florianópolis, SC, Brazil; ⊥ Department of Microbiology, Immunology and Parasitology, Universidade Federal de Santa Catarina, 88040-900 Florianópolis, SC, Brazil

## Abstract

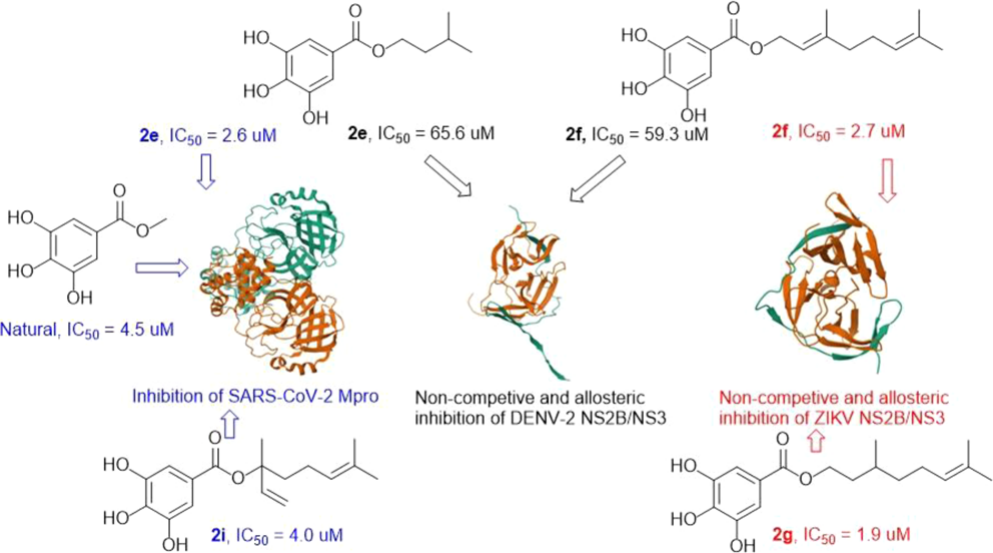

Chemical studies of *Mangifera indica* twigs yielded two compounds, identified as taraxerol (**1**) and methyl gallate (**2**). The galloyl moiety was suggested as a potential scaffold that can interfere with proteases by previous biological investigations on SARS-CoV-2 main protease (M^pro^) inhibitors in combination with docking studies. Therefore, a series of 13 gallate esters were prepared by treating gallic acid with natural and non-natural alcohols. Their inhibitory effects were evaluated against M^pro^ and NS2B/NS3 of Zika and Dengue viruses. Among the obtained compounds, **2e** and **2i** were the most potent against M^pro^ with IC_50_ values of 2.60 and 4.0 μM, respectively. Compounds **2f** and **2g** were more potent than others against ZIKV protease with IC_50_ values of 2.7 and 1.9 μM, respectively. The bioactivity profile against DENV NS2B/NS3 was different with **2e** and **2f** showing moderate inhibition with IC_50_ values of 66.0 and 59.29 μM, respectively. It was found that **2f** and **2g** inhibited ZIKV NS2B/NS3 via a noncompetitive mechanism. The study also showed that **2e** and **2f** could exert noncompetitive inhibition at the previously described allosteric pocket of flaviviral NS2B/NS3 proteases. Molecular docking revealed different types of interactions among the most prominent were hydrogen bonding with the galloyl moiety.

## Introduction

The control of SARS-CoV-2 infection completely relies on the vaccines. However, their efficiency and safety as well as the virus lethality are still being undermined in many countries.^1^ This lack of consideration, together with the disruption caused by the coronavirus pandemic to epidemiological control programs for tropical infectious diseases, is challenging the healthcare systems of tropical countries in this postpandemic era.^2^ These two elements are partially responsible for the growing rate of COVID-19 infections in patients suffering from comorbidity or coinfected with tropical infectious diseases such as Zika, Dengue, Chikungunya, Leishmaniasis, Chagas disease, and others.^3,4^ From 2020 to 2023, numerous COVID-19 cases coinfected with either influenza A virus (H3N2), Chagas disease, malaria, Dengue or Zika were registered.^5−9^

Dengue and Zika are mosquito-borne viral infections caused by arboviruses DENV and ZIKV. For Dengue, one or more serotypes including DENV-1, DENV-2, DENV-3, and DENV-4 can be responsible for the infection. From January to February 2024, Brazil registered around 600 thousand Dengue infection cases (caused mostly by DENV-2 and probably DENV-3) with almost 100 deaths.^10^ Like Zika, the Dengue infection has no specific medicine, and its treatment relies on controlling symptoms.

The resurgence of DENV-3 in Brazil and the cocirculation of viruses such as SARS-CoV-2, H3N2, DENV-2, Chikungunya (CHIKV), and ZIKV among others became an epidemiological concern in Brazil.^11^ Popular knowledge of herbal medicines has boosted the use of plant-derived products to mitigate the symptoms of these viral infections in Brazil. Decoctions from *Carica papaya* or *Euphorbia hirta* leaves are used for the treatment of Dengue fever.^12^ Reports revealed that the bitter apricot known as *Prunus armeniaca* L (seeds), *Pinellia temata* (rhizome), *Trichosanthes kirilowii* Maxim. (dried ripe fruit), *Curcuma longa* (rhizome), *Patrinia scabiosaefolia* Fisch and *Patrinia villosa* Juss (aerial part) and others used as adjuvants to Western medicines, significantly improve COVID-19 patients’ symptoms.^13^ In this sense, the present collaborative studies focus the investigation on the edible and medicinal plant, *Mangifera indica* (Anacardiaceae) not as a mixture with other plants but to identify secondary metabolites that possess inhibition potential against virus proteases. The study of *M. indica* was motivated by previous studies found in the literature. Its pulp crude extract showed intracellular antiviral activity against the influenza virus, H9N2.^14^ Also, this plant possesses antimalarial activity as previously reported.^15^ Fermented papaya and mango pulp alongside other ingredients resulted in a complex that showed antiviral activity against the Zika virus.^16^ Essential oil from mango leaves displayed insecticide properties against the Zika/Dengue main vector meanwhile it also inhibited this mosquito egg hatching.^17^

Phytochemicals of *M. indica* are well-documented with the most reported being benzoic and phenolic acids (benzoic acid, gallic acid and its esters), flavonoids (catechin, quercetin), xanthone (mangiferin), terpenoids (Friedelin and β-sitosterol) and others.^18^ Part of the biological properties reported on *M. indica* such as antibacterial, anti-inflammatory, antimalarial, and cardioprotective activities were associated with mangiferin, quercetin, catechin, and gallic acid.^18^ However, this is the first study of gallate ester against cysteine and serine proteases.

Based on the above information, *M. indica* plant was studied chemically. The obtained compounds were subjected to chemical modifications and tested against M^pro^ and nonstructural proteins (NS2B/NS3) of ZIKV and DENV-2.

The protein M^pro^ plays essential biological functions in the coronavirus life cycle characterized by proteolytic reactions to produce small proteins important for virus replication.^19^ Likewise, NS2B/NS3 in ZIKV and DENV-2 also have a proteolytic cleavage function of polyproteins to produce essential proteins for the maturation of these viruses.^20^ Therefore, impairing these proteins’ biological functions could attenuate the virus replication making them attractive drug targets.

## Materials and Methods

All reagents and solvents were acquired from local suppliers (Servylab, a Sigma-Aldrich representation in Brazil, Rauter, Vetec, and Synth, São Paulo). Silica 70–230-mesh (0.063–0.2 mm) was used for chromatographic columns. Thin layer chromatography (TLC) was performed on silica gel 60F supported on aluminum sheets with the fluorescent indicator UV254–366 (Macherey-Nagel, Germany) 0.20 mm. Melting points (m.p.) were measured using the melting point device Microquímica (MQAPF-302, São Paulo). Infrared (IR) spectra were acquired on a Bruker α model spectrometer. KBr pellets were used for all measurements and the acquisition range was 4000–400 cm^–1^. All NMR data were recorded using Bruker equipment, which included an AVANCE DRX-400 and DPX-200 as well as a Bruker Fourier 300 spectrometer (Bruker, Germany). The Xevo GS-2 QTof mass spectrometer, which was acquired from Waters, USA, was used for all mass spectrometry analyses.

### Experimental Section

Sample collection and Extraction: The plant material (twigs) was collected in March 2021 at the Trindade campus of the Federal University of Santa Catarina (UFSC), located at Florianópolis, Santa Catarina State, Brazil. The species was identified as *M. indica* at the botanic department at UFSC by matching with a voucher kept at the herbarium of INPA (Instituto Nacional de Pesquisas da Amazônia) under the registration number 234423. The dried twig (1 kg) was macerated for 7 days with dichloromethane (DCM)/methanol (1:1, v/v) at room temperature. The resulting extract was filtered and dried in vacuo at 40 °C to yield the crude extract (271.5 g). Part of this dark organic solid (200 g) was poured onto H_2_O and liquid–liquid separated using hexane (Hex), DCM, and *n*-butanol. The solvent removal from each organic phase was carried out under reduced pressure by rotary evaporation to yield the respective fractions (F1–F4).

### Isolation and Structure Determination

**Figure 1:**
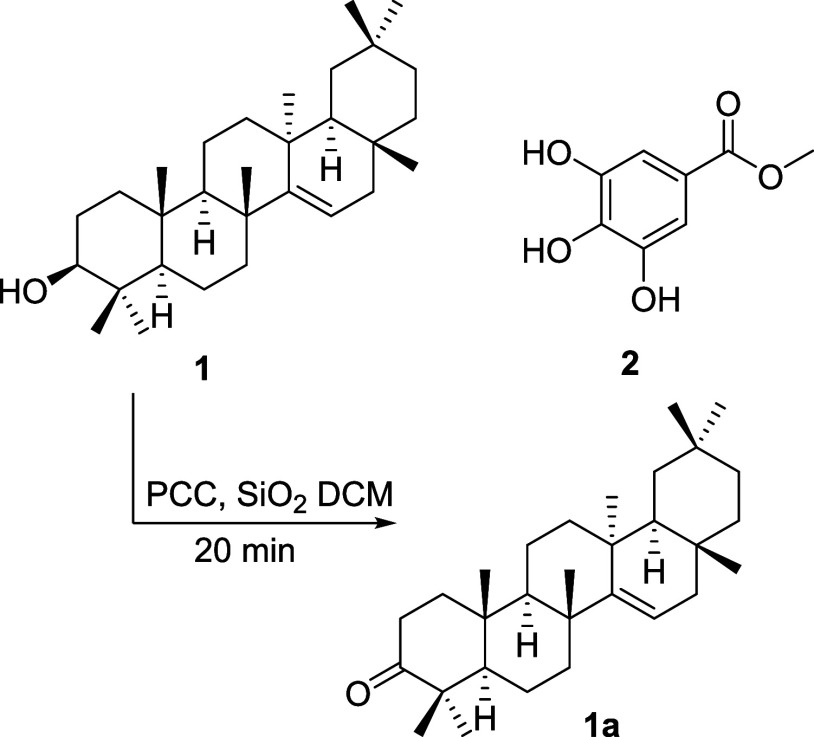
Structures of compounds **1**, **2**, and taraxerone (**1a**).

Compound **1** was obtained from fractions F1 and F2. Its purification was possible by column chromatography on silica gel using the mixture of Hex and ethyl acetate (EA) in increasing polarity. An amorphous solid in subfractions eluted with the mixtures Hex/EA (85:15 to 90:10). A quantity of 120 mg of compound **1** was obtained by recrystallization in ethanol of these subfractions. Compound **2** was isolated in the butanol fraction (F3). Gradient conditions of the mixture DCM and methanol (MeOH) were used as mobile phases for the silica gel chromatographic column. Compound **2** (300 mg) was obtained from fractions eluted with the mixture DCM/MeOH (95:5 to 90:10). Their structures were determined as taraxerol (**1**) and methyl gallate (**2**) by using NMR data (Supporting Information) which were compared to those previously reported in the literature (Figure 1).^21,22^

### Experimental Procedure for the Oxidation of Taraxerol

Several oxidation reactions using only PCC in1–3 equiv were attempted. Part of these temptations led to incomplete oxidation and others lasted more than an hour to consume completely the starting material. Therefore, taraxerol (30 mg) was subjected to an oxidation reaction using pyridinium chlorochromate PCC (5 equiv) supported by silica (1:1 weight). The reaction was performed at room temperature in 2 mL DCM for 20 min. TLC was used to monitor the oxidation until complete consumption of the substrate. The product was liquid–liquid extracted 3 times in H_2_O/DCM (1:1). Anhydrous Na_2_SO_4_ was used to remove the remaining drops of water from the organic layer. Furthermore, the mixture was filtered, and rotary evaporated. A silica gel column chromatography of the remaining solid, eluted with hex/EA (9:1) in an isocratic condition provided taraxerone **(1a)** with an 85% yield.

### Experimental Procedure for the Synthesis of Gallate Esters

**Figure 2:**
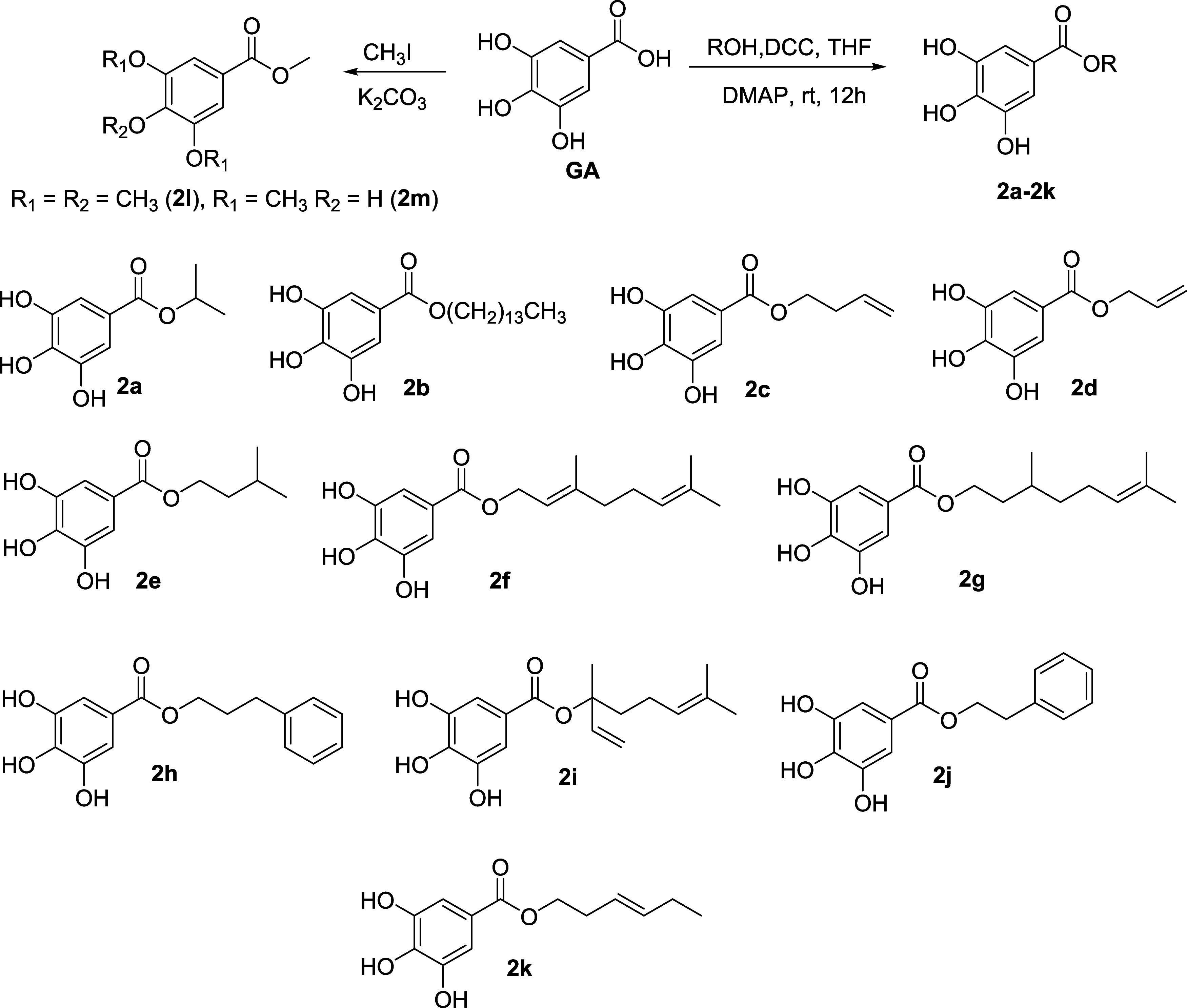
Preparation condition of gallate esters and their chemical structures.

To a THF solution (3 mL) of gallic acid (0,88 mmol) and the corresponding alcohol (2 mmol), *N*,*N*′-dicyclohexylcarbodiimide DCC (1.33 mmol) was added. The temperature of the medium was reduced to near 0 °C using an ice bath for 1 h. The reaction was then treated with DMAP (0.147 mmol) and stirred at room temperature. The progress of these experiments was followed by TLC. Reaction mixtures were concentrated by rota-evaporation in vacuo. The products were purified by silica gel column chromatography using isocratic DCM/MeOH (98:2). Eleven esters (**2a**-**2k**) were obtained with 65–90% yield. Compounds **2l** and **2m** were obtained by treating gallic acid (100 mg) with 4.1 equiv of CH_3_I at room temperature. Methyl 3,4,5-trimethoxybenzoate (**2l**) and methyl 3,5-dihydroxy-4-methoxybenzoate (**2m**) were obtained with yields of 20 and 18%, respectively. All the structures (Figure 2) were characterized by using spectroscopic and spectrometric data (Supporting Information).

### Inhibition Assays of the Obtained Compounds on SARS-CoV-2 M^pro^

Inhibitory effects against M^pro^ of all compounds were evaluated based on the experimental procedure provided by the manufacturer with some modifications.

Ten milliliters (mL) of chemicals dissolved in DMSO, with a final concentration of 200 μM, were put in triplicate to each well of a 96-well flat bottom microplate. After that, the plate was incubated for 10 min at room temperature with 30 μL of Mpro enzyme (Sigma-Aldrich) at a final concentration of 10 μg/mL diluted in buffer containing 25 mM HEPES (Sigma-Aldrich) and 0.2% of Tween-20 (Sigma-Aldrich). Furthermore, the chromogenic peptide substrate (Thr-Ser-Ala-Val-Leu-Gln-pNA, Sigma-Aldrich) was added in a volume of 10 μL to each well, at a final concentration of 200 μM and incubated for another 45 min at 37 °C. Following the incubation period, the microplate was read at 405 nm using a Tecan Model Infinite M200 spectrophotometer after 50 μL of 4% acetic acid (Merck) was added. As a positive control, ebselen was used at a concentration of 10 μM in DMSO, and a final concentration of 20% DMSO was employed as a negative control. As directed by the manufacturer, with minor modifications, the experiment was carried out to ascertain the half-maximum inhibitory concentration (IC_50_) that may block the activity of the M^pro^ enzyme. The experiment involved filling each well in a 96-well plate with 10 μL of the final concentrations of 150, 30, 6, and 1.2 μM of each compound, diluted in DMSO, in triplicate. Next, 30 μL of M^pro^ enzyme (Sigma-Aldrich), diluted in buffer containing 25 μM HEPES (Sigma-Aldrich) and 0.2% of Tween-20 (Sigma-Aldrich), was added to each well. The plate was then kept at room temperature for 10 min. Following this time interval, 10 μL of a 200 μM final concentration of chromogenic peptide substrate (Sigma-Aldrich) was added to each well, and the mixture was incubated for an additional 45 min at 37 °C. Subsequently, the microplate was read at 405 nm using a Tecan Model Infinite M200 spectrophotometer after 50 μL of 4% acetic acid (Merck) was added. As a positive control, ebselen was diluted to a concentration of 10 μM in DMSO, and a final concentration of 20% DMSO was employed as a negative control.^23,24^

### Cytotoxicity Activity of Target Compounds against THP-1 and Vero Cell Lines

In a 96-well flat bottom microplate, THP-1 cells (12 × 10^4^ cells/well) were seeded with 180 μL of RPMI 1640 medium (Sigma-Aldrich) without phenol red supplemented with 10% FBS (Sigma-Aldrich), 2 mM l-glutamine (Gibco), 1 μM sodium pyruvate (Sigma-Aldrich), 10 U/mL penicillin (Gibco), and 10 μg/mL streptomycin (Gibco) in the presence of 100 ng/mL of phorbol-12-myristate-13-acetate (PMA) (Sigma-Aldrich). The microplate was then incubated at 37 °C in a 5% CO_2_ atmosphere for 72 h. Vero cells (four times as many cells as a well) were seeded in 180 μL of RPMI 1640 media (Sigma-Aldrich), which was then supplemented with 10% FBS (Sigma-Aldrich), 10 U/mL penicillin (Gibco), and 10 μg/mL streptomycin (Gibco). The cells were then incubated for 24 h at 37 °C in a 5% CO_2_ environment. Each target chemical, diluted in RPMI-1640 media (Sigma-Aldrich) without FBS, at doses of 150, 50, 16.7, and 5.6 μM, was applied in 20 μL to each of the two cell lines. The positive and negative controls were 50 and 0.3% DMSO (Sigma-Aldrich), respectively. The plates were incubated in an environment containing 5% CO_2_ for 67 h at 37 °C. Next, 20 μL of resazurin (250 μg/mL) from Sigma-Aldrich was added, and the mixture was incubated at 37 °C for 5 h. The microplate was measured using a Tecan Model Infinite M200 spectrophotometer at 530 and 590 nm in wavelengths. Every experiment was run three times.^23,24^

### Inhibition Assays on Zika/Dengue Serine Protease

Before evaluating the compounds for their enzymatic inhibitory effects, the optimal conditions of the bioassays were checked based on previously reported standard methods.^25,26^ The linearity of the progress curves during the measurement time in the absence of an inhibitor was confirmed. Thus, the enzyme was incubated with different concentrations of the substrate and the fluorescence was measured. It was noted at the concentration of 100 μM, the rate of reaction was no longer dependent on substrate concentration as the enzyme was saturated with the substrate.

It is also noteworthy to mention that before starting any assay, the enzyme is diluted to 1.25 × 10^–2^ μM and treated with the substrate and DMSO (as negative control). After incubation, the set is submitted to the plate reader to evaluate the quality and the activity of the enzyme.

A fluorogenic substrate-based assay (substrate: Boc-Gly-Arg-Arg-AMC) was utilized to measure the inhibitory activity of the compounds against both DENV-2 and ZIKV proteases.^25,26^ Stock solutions of the substrate and inhibitors were prepared in DMSO. The assay buffer had a pH of 9.0 and contained 1 mM CHAPS, 50 mM Tris, and 20% glycerol.

In two distinct experiments, each conducted in triplicate, the measurements were carried out using a Tecan Infinite F2000 PRO fluorimeter in flat-bottom 96-well microtiter plates from Greiner Bio-One. A total of 180 μL of buffer, 5 μL of the enzyme solution (1.25 × 10^–2^ μM), 10 μL of the inhibitor in DMSO or pure DMSO as a control, and 5 μL of the substrate solution with a final concentration of 100 μM were added to each well to generate a total volume of 200 μL. Compounds were tested at concentrations of 20 and 100 μM; those with at least 50% inhibition within the screening range were chosen for IC_50_ determination. IC_50_ values were determined with a dilution series between 1000, 50, 20, 5, 2, 0.02, and 0.002 μM.

For a total period of 10 min at 25 °C, the fluorescence was measured every 30 s using 380 nm excitation and 460 nm emission wavelengths. Using GraFit from Erithacus Software Limited, the IC_50_ values were calculated by fitting the residual enzymatic activity to the four-parameter IC_50_ equation.
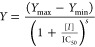
*Y* [Δ*F*/min] represents the rate of substrate hydrolysis, *Y*_max_ is the highest value of the dose–response curve obtained at an inhibitor concentration of [*I*] = 0 μM, *Y*_min_ is the lowest value achieved at high inhibitor concentrations, and *s* is the Hill coefficient under these conditions.^27^ Applying the Cheng-Prussof equation to adjust the IC_50_ -values to zero substrate concentration yielded the *K*_*i*_-values for active-site directed, competitive inhibitors^28^
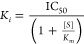
with substrate concentration *S* = 100 μM and Michaelis constant *K*_m_ = 52.9954 μM.

### Binding Mechanisms

Binding kinetics were tested for noncompetitive inhibition of ZIKV by measuring the IC_50_ values at four different substrate concentrations (Boc-Gly-Arg-Arg-AMC; 50, 100, 150, and 200 μM) and the data was plotted as IC_50_ plots and Dixon-plots with inhibitor concentration range of 5, 3, 1, and 0.1 μM for **2f** and 3, 2, and 0.2 μM for **2g**.^29^

### Allosteric Binding

The allosteric binding site of DENV2 protease was evaluated by single cysteine mutagenesis studies. Compounds **2e** and **2f** were tested against the protease with a single cysteine point mutation (T122) and their reaction with *N*-benzylmaleimide (BMI), which blocks the binding of inhibitors to the allosteric pocket. IC_50_ values were determined for the DENV2 wild-type protease, the T122C mutant protease, and the T122C mutant and DENV2 wild-type protease after incubation with BMI at a concentration of 250 nM. It is important to mention that BMI is an irreversible inhibitor of T122C and inhibits 100% of T122C at a concentration of 250 nM.^26^ All assays were performed with concentrations ranging between 500, 300, 100, 10, 1, and 0.1 μM.^26^

### *Z* Factor

The *Z* Factor is a useful parameter for evaluating the quality of an assay since it captures both the dynamic range of the assay signal and the data fluctuation related to the signal measurements. Because the *Z* factor is a straightforward and dimensionless statistical feature for every screening test, it can be utilized in test optimization and validation as well as for comparison and quality assessment. Only when the *Z* factor value was ≥0.5 were all conducted tests considered valid.

### Statistical Analysis

Using GraphPad Prism 8.0 (GraphPad Software, Inc., CA), the IC_50_ and CC_50_ values were determined by nonlinear regression of the dose–response curve and were reported as mean ± standard deviation (SD). Every experiment was run three times (*n* = three).

### Structure–Activity Relationship Based on Molecular Docking

Molecular docking simulations of the active compounds were conducted using targets associated with SARSCoV-2 Main protease (PDB ID: 5RG1,^30^5RG2,^30^5RG3^30^ and 6LU7(31)), DENV-2 NS2B/NS3 protease (PDB ID: 2FOM),^32^ this enzyme was subjected to mutations by replacing the Thr122 residue with cysteine and Zika virus NS2B/NS3 protease (PDB ID: 7VLG).^33^ Molecular docking simulations were carried out to evaluate the possibility of these compounds being related to any of the mechanisms under study based on the assessment of affinity in kJ mol^–1^. Three scoring functions were utilized, which correlated to the MolDock Score, Plants Score, and Rerank Score, respectively. The calculations were done based on the energy score of the MolDock Score and Plants Score algorithms. The enzymes’ three-dimensional structures were sourced from the Protein Data Bank (PDB) at https://www.rcsb.org/pdb/home/home.do.^34,35^ These structures cocorrespond to the structure of SARS-CoV-2: Main Protease in complex with *N*-α-acetyl-*N*-(3-bromoprop-2-yn-1-yl)-l-tyrosinamide (PDB ID: 5RG1), with a resolution of 1.65 Å and X-ray diffraction (XRD) technique. With a resolution of 1.63 Å and an X-ray diffraction technique, the second three-dimensional (3D) structure matched the target Main Protease in complex with NCL-00025058 (PDB: 5RG2).

The third structure, which had a resolution of 1.58 Å and was determined by X-ray diffraction, matched the target Main Protease in association with NCL-00025412 (PDB: 5RG3). The target Mpro in combination with the inhibitor N3 was represented by the fourth structure (PDB: 6LU7). Two protein structures were utilized to research arboviruses: ZIKV NS2B-NS3 with compound MI2201 (PDB: 7VLG), Resolution: 1.77 Å, and Dengue virus NS2B/NS3 (PDB: 2FOM), Resolution: 1.50 Å, and method: X-ray diffraction.

The binding sites of the proteins under investigation were identified and incorporated into the analyses based on bibliographic research. The Protein Data Bank (PDB) library reference articles (http://www.pdb.org), whose references were previously referenced, provided the active-site information that was used to define the active-site region. Since cocrystallized ligands are present in the four enzymes chosen for SARS-CoV-2 and Zika, the active site was determined using a template created by the coordinates of the ligand in contact with the protein. Using the Bite Net Platform - Skoltech I Molecule, 2022 (https://sites.skoltech.ru/imolecule/tools/bitenet) and molecular pocket predictions, the allosteric site for the enzymes related to the DENV-2 protease was determined based on residues reported in the literature.

For Dengue protease (PDB: 2FOM) the coordinates of the allosteric site corresponded to X:-14.73, Y:-10.49 and Z:16.03, with residue Thr122 being observed. For Dengue virus protease, the compound DNTB (5,5′-dithiobis(2-nitrobenzoic acid)) was used as the allosteric control.^36^ The chemicals were first shown using the Marvin Sketch v software. 19.18 (https://chemaxon.com/marvin)^37^ and stored as. sdf files. The Standardizer v program was then used to standardize the chemicals. 21.2.0 ChemAxon (https://chemaxon.com/standardizer), where the aromatic ring’s standardization, salt removal, hydrogen atom addition, and structure conversion to three dimensions were completed. Following this, the compounds under investigation were put through a molecular docking simulation. To do this, the water molecules were eliminated and a “template” was made between the macromolecule under investigation and the cocrystallized ligand with the goal of identifying the enzyme’s active site. The test molecules were then inserted, and the molecular docking simulation was run. Redocking was done in order to determine whether the program was producing the poses correctly before molecular docking. Redocking represents the root-mean-square deviation (RMSD), which is deemed successful if the result is less than 2.0 Å.

The active site was defined by the complex ligand. Subsequently, the compounds were imported in order to analyze the system’s stability using the interactions found with the enzyme’s active site. The energetic value of the MolDock Score—which was calculated using the default parameters of Molegro Virtual Docker v.6.0.1 (MVD) software^38^—and the PLANTS Score algorithms^39−41^ were used as references.

The following parameters were applied while using the MolDock SE (Simplex Evolution) algorithm: 30 runs altogether, with a maximum of 3000 interactions, utilizing a 50-person population, 2000 global minimization steps for every run, and 2000 minimization steps for every flexible residue. Docking energy values were computed using the MolDock Score (GRID), Rerank Score, and PLANTS Score (GRID) scoring tools. The search sphere was set at a radius of 15 A, and a GRID was set at 0.3 A. Internal electrostatic interactions, internal hydrogen bonds, and sp^2^-sp^2^ torsions were assessed in order to analyze the ligand energy.

The software Discovery Studio Visualizer v20.1.0.19295 - BIOVIA (2020) (https://discover.3ds.com/discovery-studio-visualizer-download) was used to display the interactions and acquire the molecular docking figures.^42^ Thr122 was changed to cysteine in order to carry out the mutation, which was done with the Chimera 1.17.3 program (https://www.cgl.ucsf.edu/chimera/).

### Docking Consensus

Three distinct scoring functions were used in a consensus analysis to reduce the amount of false positives. The MolDock, Rerank, and PLANTS scores of the compounds that were the subject of the affinity studies were taken into account when calculating the consensus. First, the value of (*p*), which is the result of dividing the score each compound received by the compound with the lowest energy, was determined for each of the scoring functions that were the subject of the investigation. eq 1
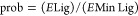
1*E* Lig represents the energy that each ligand in the generated docking simulation obtained. The lowest energy among the ligands under investigation is represented by the *E*Min Lig parameter. The computation of the overall average across all biomarkers under investigation is referred to as the second consensus analysis. In this manner, a general average of the probability found for each biomarker is used to calculate the probability values for each of the substances being studied. Equation 2 states that the total probability (*P*), which is the sum of the probability values obtained for each of the scoring functions in the study divided by the total number of observations, is determined after the probability values for the compounds in each of the scoring functions under study have been obtained.
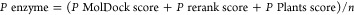
2

### Molecular Dynamics Simulation

The flexibility of interactions between proteins and ligands was estimated by Molecular Dynamics simulations, utilizing the GROMACS 5.0 program (European Union Horizon 2020 Program, Sweden).^43,44^ The GROMOS96 54a7 force field was also utilized to build protein and ligand topologies. The point charge SPC water model, stretched in a cubic box, was used for the MD simulation.^45^ In order to eliminate inadequate connections between complicated molecules and the solvent, the system was neutralized by adding ions (Cl– and Na^+^) and then minimized. Additionally, the system was equilibrated at 1 atm pressure using the Parrinello–Rahman algorithm as the NPT (particle constant pressure and temperature), up to 100 ps, and balanced at 300 K using the 100 ps V-rescale algorithm, which is represented by NVT (constant number of particles, volume, and temperature). MD simulations were run at 10 ns in 5 × 10^6^ stages. The relative mean square deviation (RMSD) values of all Cα atoms with respect to the original structures were computed in order to assess the flexibility of the structure and whether the complex is stable closer to the experimental structure. In order to comprehend the functions performed by residues at the receptor binding site, RMSF values were also examined. The Grace software (Grace Development Team, http://plasma-gate.weizmann.ac.il/Grace/) was used to create RMSD and RMSF graphs, whereas UCSF Chimera was used to view proteins and ligands.^46−48^

## Results and Discussion

The crude extract of twigs and leaves collected from the mango tree was subjected to successive chromatographic columns. Over this purification, two compounds were substantially obtained namely taraxerol (**1**) and methyl gallate (**2**). Their structures (Figure 1) were established by ^1^H- and ^13^C NMR data compared to those reported in the literature.^21,22^ The first enzymatic screening at 200 μM for all compounds against M^pro^ showed inhibitory percentages of 48 and 68% for compounds **1** and **2**, respectively. Based on these findings, compound **1** was submitted to an oxidation reaction (Figure 2) to afford taraxerone (**1a**) and then tested for its inhibitory effect against M^pro^. At the initial concentration of 200 μM, it showed an inhibitory percentage of 84% against M^pro^. However, further experiments were not possible as compound **1a** showed low solubility during the biological testing. Because of the lipophilicity of compounds **1** and **1a**, we focused mostly on developing gallate derivatives. Thus, Molecular docking using three M^pro^ protein structures (Protein Data bank [PDB] entries 5RG1–3 and 6LU7) was applied to a series of alkyl gallates that were planned. This study was performed assuming that the binding of the compounds occurs in the active site. Three scoring functions namely MolDock, PLANTS, and Rerank showed scores that average are displayed in Table 1. Based on the obtained data, part of these compounds showed high probabilities to inhibit SARS-CoV-2 M^pro^. Compounds **2a**–**k** (Table 1) disclosed probabilities higher than Ebselen on 5RG1. Compound 2h (*p* = 0.882) showed the highest probability of interactions with 5RG2 while **2c** (*p* = 0.879), **2g** (*p* = 0.909) and **2k** (*p* = 0.872) showed the greatest probabilities with 5RG3. Compound **2g** displayed the highest probability (*p* = 0.779) compared to all the compounds when using 6LU7.

**Table 1:** Probability (*p*) Values Acquired by Consensus Computations from Moldock, Rerank, and Plants Score Algorithms of the Three PDB M^pro^

	PDB codes			
compounds	5RG1	5RG2	5RG3	6LU7
**2**	0.638	0.7*	0.585	0.524
**2a**	0.709	0.765	0.579	0.564
**2b**	0.684	0.504	0.611	0.723
**2c**	0.77	0.822	0.879*	0.641
**2d**	0.721	0.482	0.641	0.579
**2e**	0.775*	0.627	0.472	0.658
**2f**	0.897*	0.461	0.688	0.728
**2g**	0.962*	0.637	0.909*	0.779*
**2h**	0.882*	0.946*	0.852	0.686
**2i**	0.903*	0.584	0.579	0.749
**2j**	0.835*	0.521	0.752	0.685
**2k**	0.822*	0.817	0.872*	0.733
**2l**	0.583	0.688	0.559	0.491
**2m**	0.59	0.662	0.717	0.547
Ebselen	0.634	0.417	0.533	0.540
PDB ligand	0.740	0.499	0.854	0.666

### The Cytotoxic Action against THP-1 and Vero Cell Lines and the Inhibitory Effects against SARS-CoV-2 M^pro^

Based on the high probability of interaction presented above, a series of 13 gallate esters (compounds **2a**–**2m**) were prepared and identified by using their analytic data. None of these compounds are newly reported. However, this is the first report of these gallates against viral proteases.

Together with GA, these substances were tested in vitro for their cytotoxicity against human leukemia monocytic (THP-1) and monkey kidney epithelial (Vero) cell lines, as well as their inhibitory effects against M^pro^ (Table 2). Ebselen was used as the positive control. Nine of these compounds showed percentages inhibition against M^pro^ greater than 50% at 200 μM. The half-maximal inhibitory concentrations (IC_50_) of those presenting percentages of inhibition over 50% were determined. Four compounds namely **2e**, **2i, 2j**, and **2l** significantly inhibited M^pro^ with IC_50_ values of 2.6 ± 1.0, 4.0 ± 0.9, 0.6 ± 1.2, and 2.8 ± 3.2, respectively. Compound **2e** was more active than the positive control while **2i** was almost as potent as the positive control. Compounds **2e** and **2i** were more active than the natural product (compound **2**). Compounds **2j** and **2l** showed significant inhibition with SD values greater than the means. These results seem to be false positives as solubility problems were observed for both compounds (occurrence of white aggregates) at the end of the experiment.

**Table 2:** Compounds’ IC_50_ Values and Inhibitory Percentage against SARS-CoV-2 M^pro^, as well as Their Cytotoxicity against Vero and THP-1 Cell Lines^a^

			CC_50_ ± SD (μM)		
compounds	effect at 200 μM (%)	IC_50_ ± SD (μM)	THP-1	Vero	obs.
**1**	48.0	NT	NT	NT	
**2**	68.0	4.5 ± 1.8	52.9 ± 19.2	>150	
**1a**	84.0	ND	NT	NT	SP^#^
**2a**	78.0	20.7 ± 8.2	78.5 ± 8.1	>150	
**2b**	NA	NT	NT	NT	
**2c**	80.4	35.8 ± 4.5	38.8 ± 4.4	>150	
**2d**	94.3	12.4 ± 2.4	28.5 ± 3.2	>150	
**2e**	99.5	2.6 ± 1.0	47.0 ± 6.5	>150	
**2f**	97.0	9.5 ± 1.0	72.1 ± 7.3	48.4 ± 13.2	
**2g**	73.3	88.7 ± 11.6	32.1 ± 3.4	82.1 ± 31.5	
**2h**	45.1	NT	NT	NT	
**2i**	80.0	4.0 ± 0.9	98.9 ± 5.0	>150	
**2j**	60.8	0.6 ± 1.2	22.9 ± 3.2	>150	SP^#^
**2k**	41.9	NT	NT	NT	
**2l**	55.5	2.8 ± 3.3	>150	>150	SP^#^
**2m**	44.2	NT	NT	NT	
**GA**	NA	NT	NT	NT	
Ebselen (10 μM)	90	3.4 ± 1.0	NT	NT	

a NA: no activity; NT: not tested; ND: not determined; SD: standard deviation; Obs.: observation; SP^#^: solubility problem observed at the end of the experiment.

A saturated 3-methylbutyl side chain seems to be suitable for bioactivity and the presence of a double bond was prejudicial. Moreover, alcohols containing hydrocarbon chains shorter or longer than 4 carbon atoms lost the inhibitory effects.

The probability of **2e** to inhibit PDB 5RG1M^pro^ was lower than that of Nirmatrelvir and greater than those of Ebselen and PDB ligand. Compound **2i** displayed high interaction probability with PDB 5RG1 and was almost as effective as Ebselen.

SARS-CoV-2 infection causes cell and tissue damage.^49^ Therefore, it sounds important to identify noncytotoxic substances with anti-M^pro^ potential. Thus, cytotoxicity assays revealed that except for compound **2l**, other active compounds (**2**, **2a**, **2c**–**g**, **2i** and **2j**) were moderately cytotoxic to THP-1 cells with CC_50_ values ranging from 22.9 ± 3.2 to 98.9 ± 5.0 μM, with compound **2i** being the less cytotoxic. Only esters **2f** and **2g** were cytotoxic to Vero cells with CC_50_ values of 48.4 ± 13.2 and 82.1 ± 31.5 μM, respectively.

The postpandemic era has a new epidemiological panorama in which coinfections of COVID-19 and other tropical diseases such as Zika and Dengue are reported daily. Thus, the prepared compounds were also evaluated on the ZIKV and DENV-2 proteases, NS2B/NS3.

### Inhibitory Effects against NS2B/NS3 of ZIKV and DENV-2

Compounds **2a**–**2h**, **2j**, and **2k** obtained in a substantial amount were tested against the nonstructural protease of ZIKV (NS2B/NS3) (Table 3). The first screening at a concentration of 100 μM showed inhibition close to 100% for all the compounds. So, the experiments were repeated using 5-fold diluted (20 μM) samples. Significant inhibitions were obtained for compounds **2f**–**2h** (98, 99, and 96%, respectively). Whereas IC_50_ values of these compounds were 2.7 ± 0.20, 1.9 ± 0.07, and 4.8 ± 0.53 μM for **2f**, **2g**, and **2h** respectively. The remaining compounds were moderate to weakly active with IC_50_ values ranging from 5.99 to 92.46 μM.

**Table 3:** Inhibitory Effects of Part of the Obtained Compounds against ZIKV NS2B/NS3^a^

compounds	screening at 100 μM (%)	screening at 20 μM (%)	IC_50_ (μM)
**2a**	98 ± 0.5	51 ± 3.4	19.8 ± 0.5
**2b**	55 ± 2.3	64 ± 2.5	84.9 ± 9.0
**2c**	48 ± 2.2	25 ± 2.6	>100
**2d**	53 ± 1.7	25 ± 2.2	92.5 ± 3.1
**2e**	99 ± 0.2	72 ± 3.1	13.0 ± 0.4
**2f**	99 ± 0.4	98 ± 1.1	2.7 ± 0.3
**2g**	97 ± 1.1	99 ± 0.4	1.9 ± 0.1
**2h**	99 ± 0.2	96 ± 0.4	4.8 ± 0.5
**2i**	NT	NT	NT
**2j**	82 ± 2.7	50 ± 3.8	19.7 ± 2.1
**2k**	100 ± 0.1	86 ± 0.8	6.1 ± 0.6
**2l**	NT	NT	NT
**2m**	NT	NT	NT

a NT: not tested.

Except for compounds **2i**, **2l** and **2m**, others were also evaluated for their inhibitory effects against the DENV-2 serine protease NS2B/NS3 of DENV2 (Table 4). Only compounds **2e** and **2f** showed inhibitory percentages beyond 50% with IC_50_ values of 65.6 ± 11.4 and 59.3 ± 6.9 μM, respectively.

**Table 4:** Inhibitory Effects of Part of the Obtained Compounds against DENV-2 NS2B/NS3^a^

compounds	screening at 100 μM (%)	IC_50_ (μM)
**2a**	48 ± 1	>100
**2b**	21 ± 3	ND
**2c**	18 ± 3	ND
**2d**	29 ± 1	ND
**2e**	52 ± 2	65.6 ± 11.4
**2f**	54 ± 3	59.3 ± 6.9
**2g**	28 ± 2	ND
**2h**	20 ± 1	ND
**2j**	22 ± 1	ND
**2k**	28 ± 4	ND

a ND: not determine.

### Binding Mechanism

**Figure 3:**
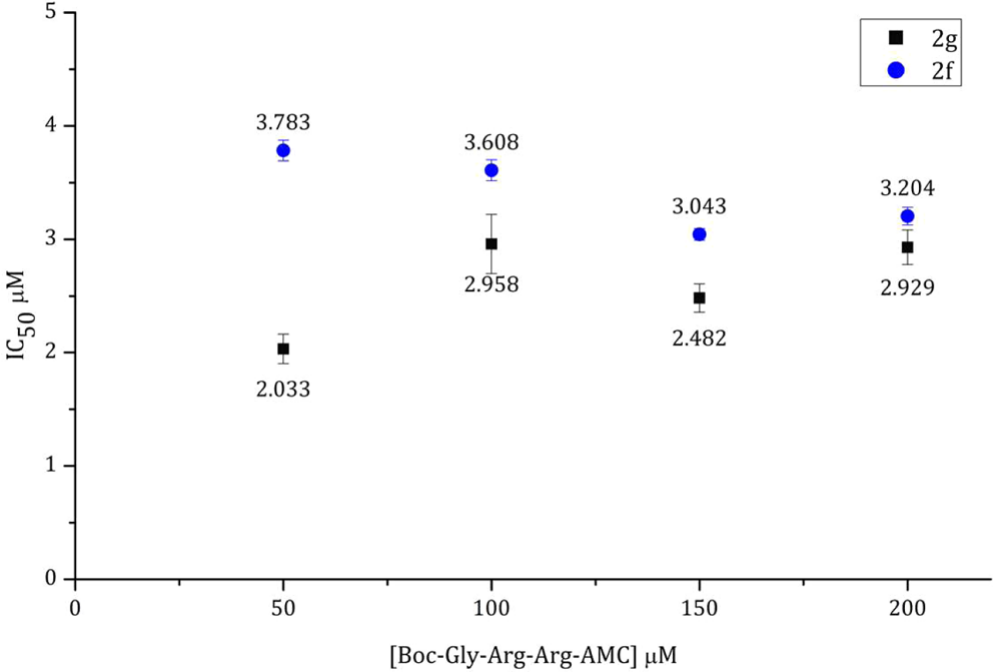
IC_50_ values of compounds **2f** (blue spot) and **2g** (black spots) at concentrations of the substrate.

To identify whether compounds **2f** and **2g** bind to the allosteric site of ZIKV NS2B/NS3, IC_50_ values of both compounds were first determined at different concentrations (200, 150, 100, and 50 μM) of the fluorogenic substrate Boc-Gly-Arg-Arg-AMC (**Sub**). Compound **2f** showed IC_50_ of 3.73, 3.60, 3.04, and 3.20 μM corresponding to the four concentrations of **Sub**, respectively. On the other hand, compound **2g** displayed IC_50_ of 2.03, 2.96, 2.48, and 2.93 μM, respectively (Figure 3).

**Figure 4:**
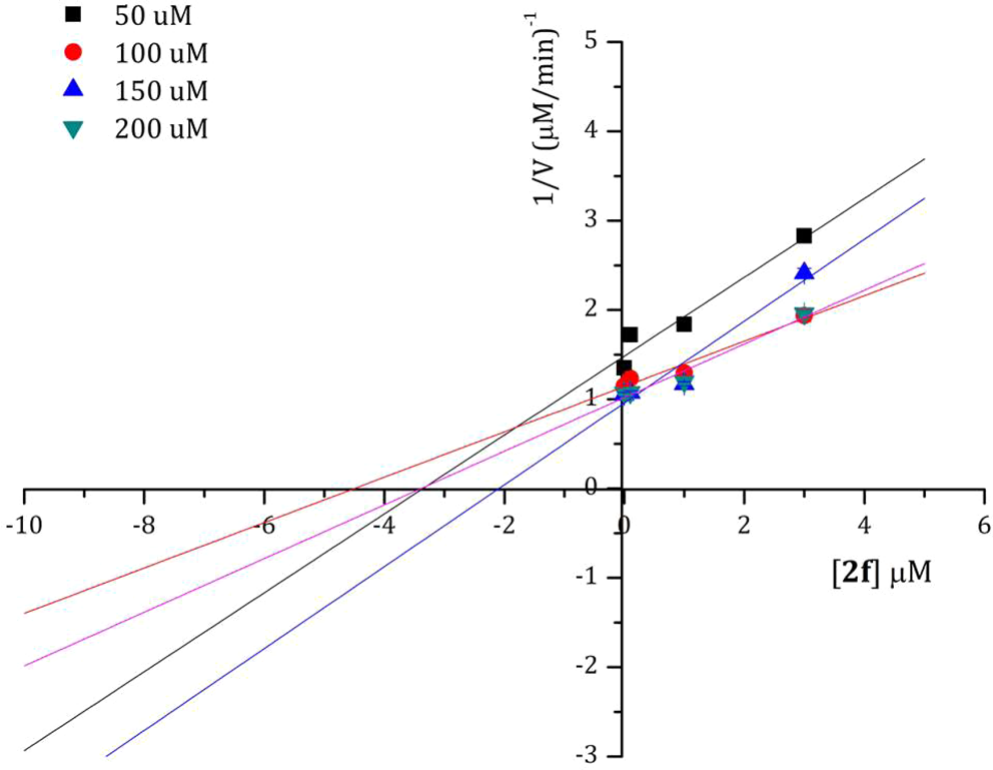
Dixon plot of compound **2f** at concentrations of the substrate.

**Figure 5:**
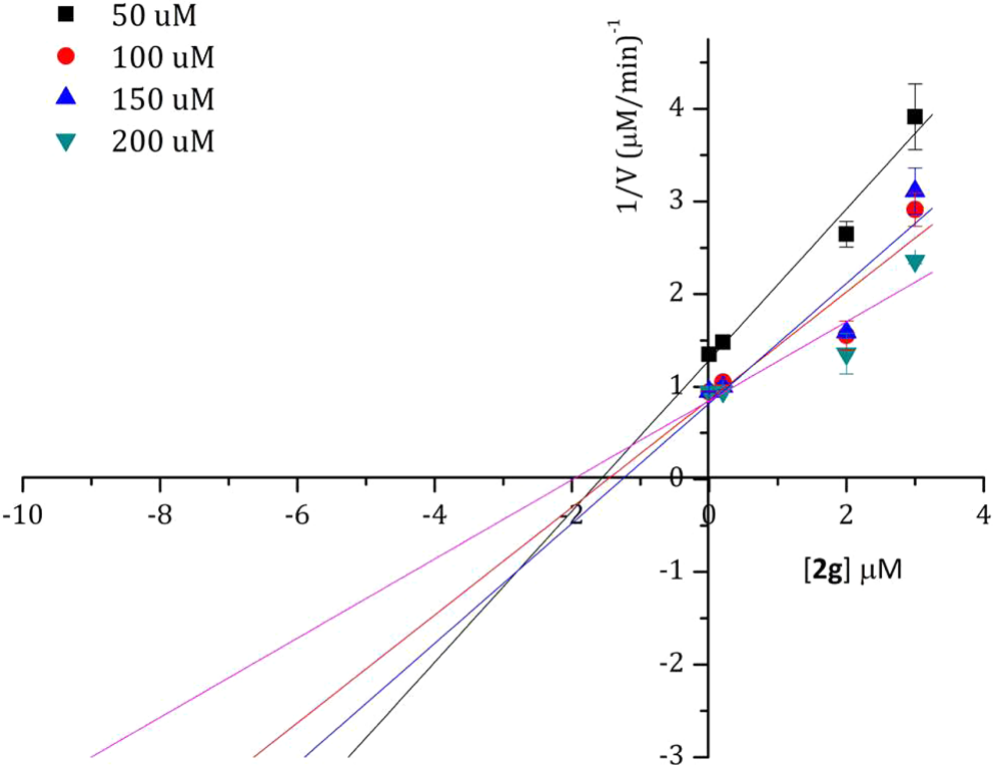
Dixon plot of compound **2g** at concentrations of the substrate.

Almost constant inhibitory effects were observed for each compound suggesting that both compounds inactivate the enzyme in a noncompetitive manner. These mechanisms were also consistent with the Dixon plots (Figures 4 and 5).

ZIKV NS2B/NS3 has several Cys residues, i.e., the construction of a single Cys mutant with the Cys residue located in the allosteric site is not possible. In contrast, DENV-2 NS2B/NS3 does not contain any Cys residues rendering single Cys mutants possible.

Allosteric pockets are usually detected by mutating particular amino acids such as T118, T120, T122, A164, and A166 to cysteine in the DENV protease. This allows us to determine whether an inhibitor binds into this pocket and to investigate which residues in the binding pocket are important for interactions.

However, a previous report revealed a series of compounds containing catechol and pyrogaloyl moiety as inhibitors of NS2B/NS3 through allosteric binding to T122C.^26,50,51^ Since we have this mutant available it sounded appropriate to check the mechanism using the same mutant.

Therefore, to study whether these inhibitors bind into an allosteric site, a mutant of DENV-2 NS2B/NS3 (T122C) was used. Due to the similarity of both proteases (from ZIKV and DENV-2), the studies also give information on the binding of inhibitors to the ZIKV NS2B/NS3 allosteric pocket. *N*-benzylmaleimide (BMI), an irreversible inhibitor of this allosteric site in T122C was used for this assay. The changes in IC_50_ values of compounds **2e** and **2f** were monitored when tested simultaneously with DENV-2 NS2B/NS3, DENV-2 NS2B/NS3+BMI, T122C and T122C + BMI.

**Figure 6:**
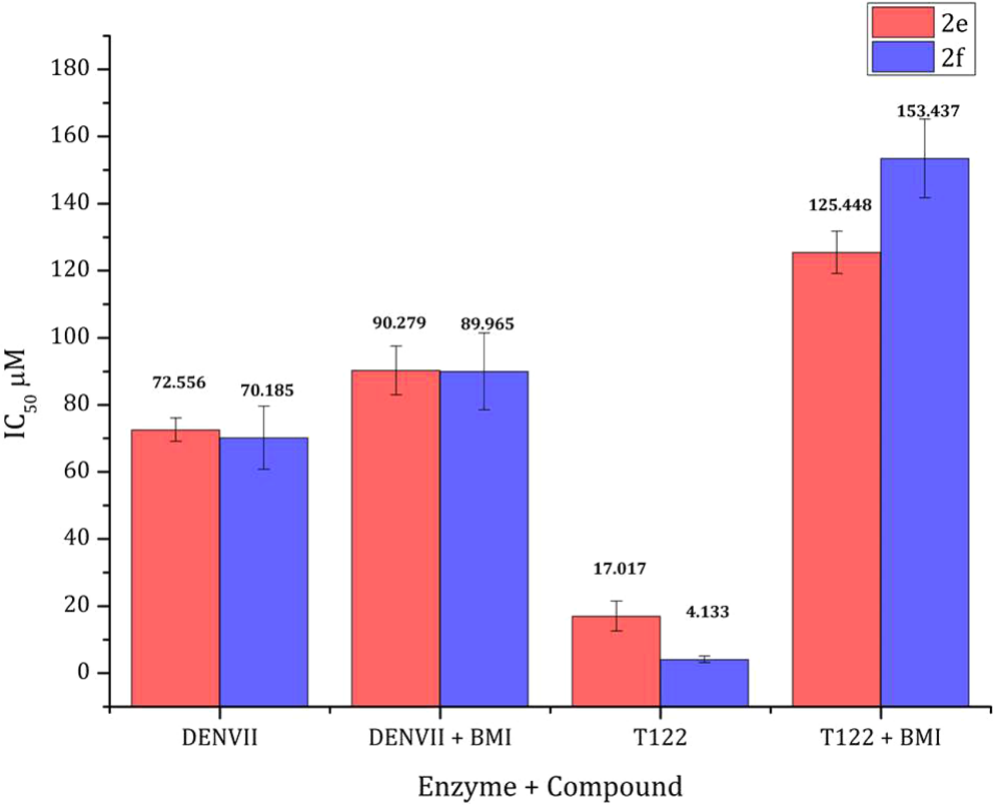
Study of the allosteric binding of compounds **2e** (red) and **2f** (blue).

As depicted in Figure 6, a slight change was observed for compounds **2e** and **2f** IC_50_ values when compared DENV2 NS2B/NS3 to DENV2 NS2B/NS3+BMI assays. These compounds significantly inhibited T122C with IC_50_ values of 17.02 ± 4.43 and 4.33 ± 0.97, respectively. In the case of T112C+BMI assays, inhibitory effects decreased by 7.4- and 37-fold for compounds **2e** and **2f**, respectively. This finding demonstrated that the T122C pocket was susceptible to the allosteric inhibition of the gallate esters.

### Docking Interaction of Compounds **2e**–**2j** and M^pro^

**Figure 7:**
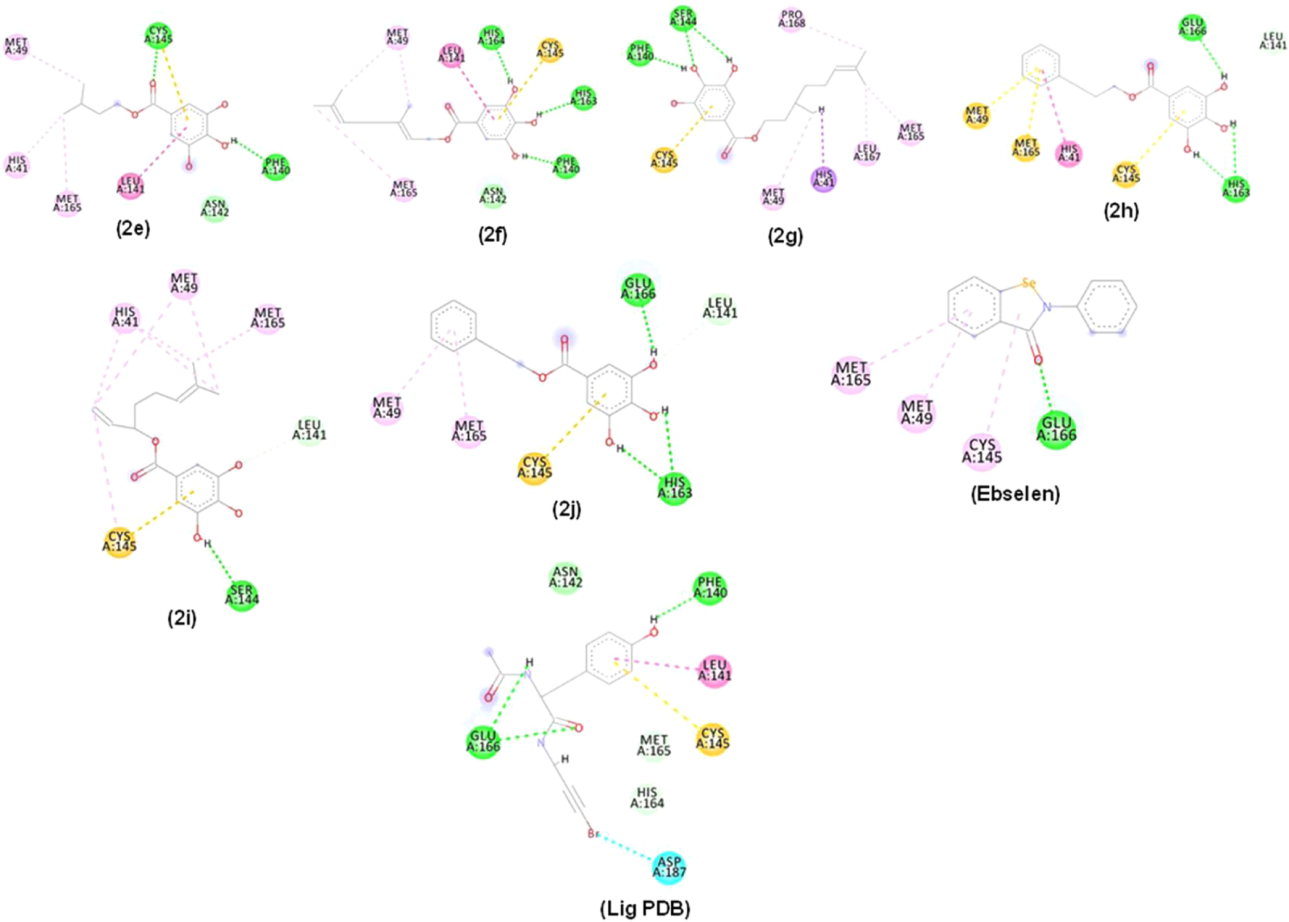
2D and 3D interactions occurred between compounds **2e**–**2j**, (Ebselen), and (lig PDB) with M^pro^ (PDB: 5RG1). Alkyl, π-alkyl, π–π stacked (pink dashed line), π–σ (lilac dashed line), hydrogen bonding (green dashed line), π-sulfur (orange dashed line), halogen (blue dashed line), and unfavorable interactions (red dashed line). Residues: Met (Methionine), Cys (Cysteine), Phe (Phenylalanine), Asn (Asparagine), Leu (Leucine), Met (Methionine), His (Histidine), Ser (Serine), Pro (Proline), Glu (Acid glutamic acid), Asp (Aspartic acid) and Arg (Arginine).

Figure 7 illustrates the interaction of the esters with PDB 5RG1, 5RG2 and 5RG3. Molecular interactions of compounds **2e**–**2j**, Ebselen, and the PDB ligand with M^pro^ PDB ID: 5RG1 active site, displayed hydrogen bond-type interactions (line dashed in green), hydrophobic interactions (dashed lines in pink) and steric or unfavorable interactions (dashed lines in red). It is important to highlight that the 3,4,5-trihydroxybenzoyl group played a fundamental role in the interaction, establishing numerous of them with the enzyme.

Compound **2e** interacted through hydrogen bonds (dashed line in green), with Cys145 (1 interaction), Phe140 (1 interaction), and Asn142 (1 interaction) (Figure 7). These interactions were observed alongside hydrophobic interactions (pink dashed lines). π-sulfur-type interactions were visualized between compound **2e** and Cys145 (1 interaction).

Compound **2f** established hydrogen bond interactions (dashed line in green) between OH groups from the aromatic system with His164 (1 interaction), His163 (1 interaction), Phe140 (1 interaction), and Asn142 (1 interaction). Its hydrophobic interactions (pink dashed lines) and π-sulfur interactions were also observed. Compound **2g** also showed π-sulfur interaction with Cys145 (1 interaction) but its hydrogen bond type interactions (dashed line in green) were between OH groups and Ser144 (1 interaction) and Phe140 (1 interaction). Compound **2h** mostly displayed hydrogen bond interactions with Glu166 (1 interaction) and His163 (2 interactions) alongside the pi-sulfur interaction type (orange dashed line) with Met49 (1 interaction), Met165 (1 interaction) and Cys145 (1 interaction). Compound **2i** interacted by hydrogen bonding with Ser144 (1 interaction) and its Pi-sulfur interaction was visualized with Cys145 residue (1 interaction). Compound **2j** showed hydrogen bonding similar to those of **2j**. Compounds **2f**, **2h**, and **2j** covalently bond to His163 in the subsites S1 while compound **2g** established the binding to Pro168 in S3. Hydrogen bonds to Leu141 by compounds **2h**, **2i** and **2j** are important for the maintenance of the enzyme side chain.^29^

### Molecular Docking Interaction into the Allosteric Site of Compounds 2e and 2f with DENV-2 NS2B/NS3 (PDB ID 2FOM)

**Figure 8:**
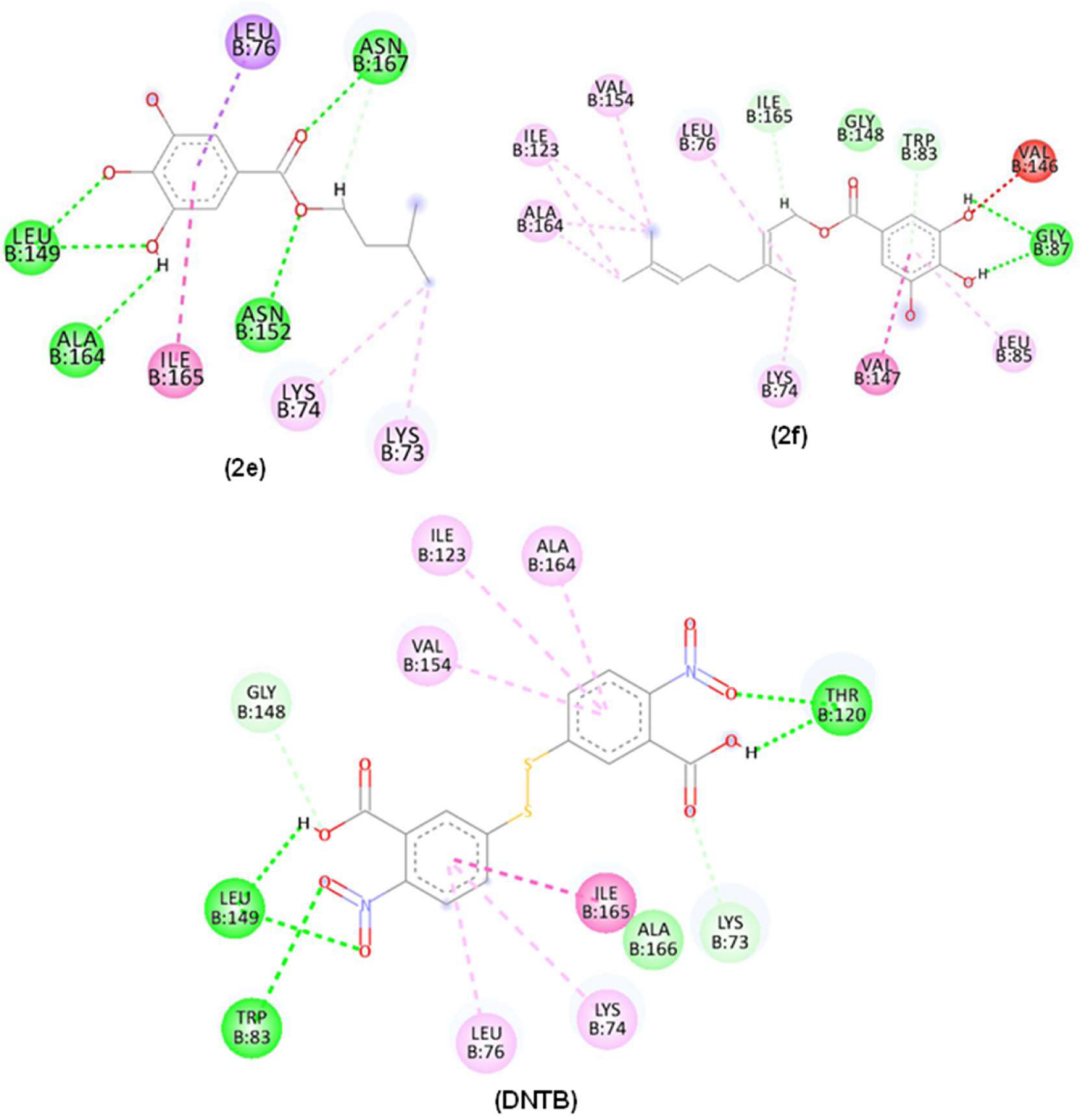
2D and 3D interactions occurred between compounds **2e**, **2f**, and control (DNTB) with the Dengue protease enzyme (PDB: 2FOM). Alky interactions, π-alkyl, π–π stacked (pink dashed line), hydrogen bonding (green dashed line), π–σ (lilac dashed line) and Unfavorable interaction (red dashed line). Residues: Thr (Threonine), Asn (Asparagine), Leu (Leucine), Ala (Alanine), Ile (Isoleucine), Lys (Lysine), Val (Valine), Gly (Glycine) and Trp (Tryptophan).

Interactions of compounds **2e** and **2f** (Figure 8) with DENV-2 protease PDB ID 2FOM were composed of H-bonds (green dashed line) and hydrophobic interactions (pink dashed lines). Compound **2e** OH groups established H-bonds with Leu149 and Ala164 while the carbonyl group was H-bonded to Asn167 and Asn152. The residues of hydrophobic interactions corresponding to Lys74 and Ala164 were visualized in all compounds. As both compounds are structurally related, similar binding modes were expected. In contrast, OH groups in compound **2f** showed H-bonds with Gly87 (Figure 8).

### In Silico Interactions of Compounds 2f and 2g with the ZIKV NS2B/NS3 (PDB: 7VLG) Allosteric Site

**Figure 9:**
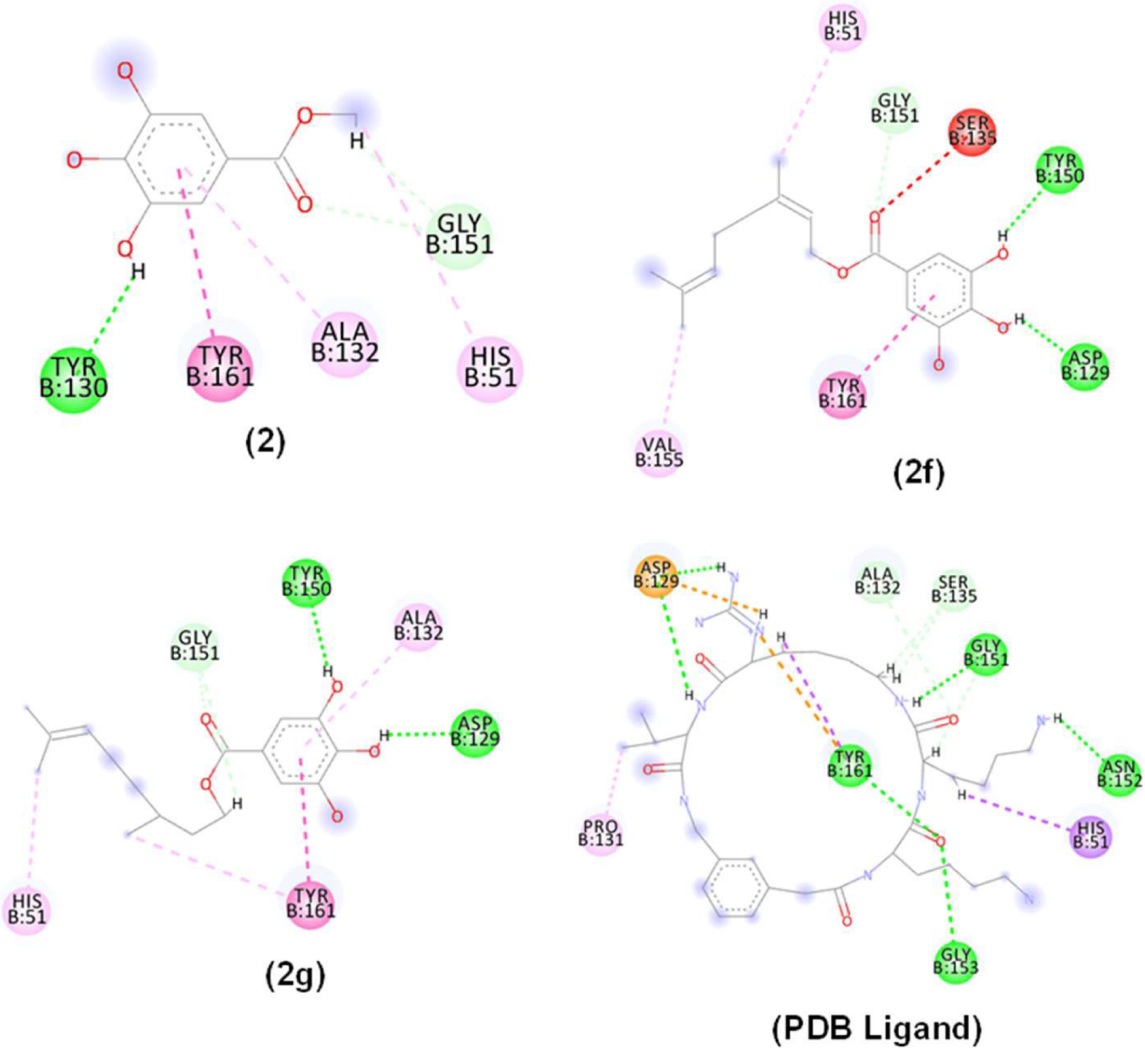
2D and 3D interactions occurred between compounds **2**, **2f**, **2g** and PDB ligand with the Zika virus protease enzyme mutation (PDB: 7LVG). Alky interactions, π-alkyl, π–π stacked (pink dashed line), hydrogen bonding (green dashed line), π–σ (lilac dashed line), π anion interaction (orange dashed line) and Unfavorable interaction (red dashed line). Residues: Tyr (Tyrosine), Ala (Alanine), Gly (Glycine), His (Histidine), Val (Valine), Asp (aspartic acid), Pro (Proline) and Asn (Asparagine).

Molecular docking studies were performed to identify possible bindings of compounds **2f**, **2g**, and the PDB ligand with ZIKV NS2B/NB3 (PDB: 7VLG) (Figure 9).

Interactions of compounds **2f** and **2g** and the Zika virus protease (PDB ID 7VLG) were mainly hydrogen bonds (dashed line in green), hydrophobic interactions (pink dashed line) and also nonfavorable interactions (dashed line in red). The OH functionalities in both compounds **2f** and **2g** acted as H-donors to Tyr150 and Asp129. Hydrophobic interactions were predominant in the side chain of the esters.

### Molecular Dynamics Simulations of Dengue Protease in Open Site (PDB ID 2FOM)

Molecular dynamics simulations were used to assess the flexibility of the enzyme and the stability of interactions in the presence of variables such as solvent, ions, pressure, and temperature, following analysis of potential activity against the DENV-2 protease (PDB 2FOM) in an open site of compounds **2e** and **2f**. The purpose of this analysis is to support the docking results and determine whether the compounds continue to be tightly bound to the enzyme in the presence of various variables.

**Figure 10:**
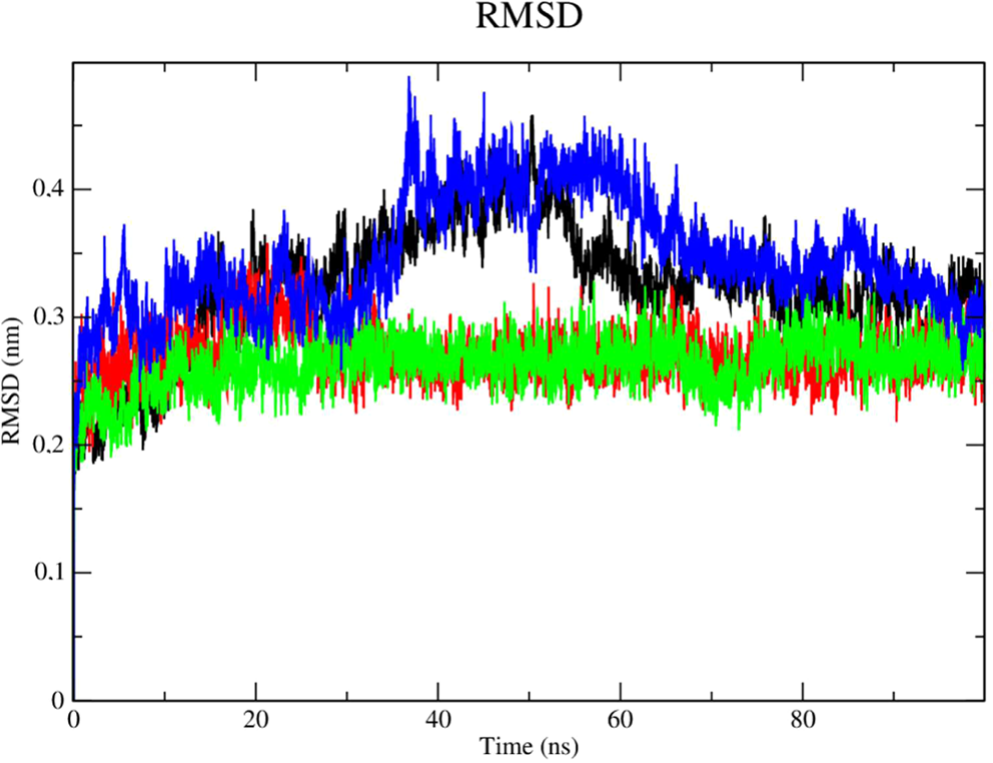
RMSD of Cα atoms. (A) of the Dengue protease (PDB: 2FOM) (black line) and binds to compounds **2e** (red line), **2f** (green line) and DNTB (blue line).

Analysis of the protein’s RMSD metric (Figure 10) showed that the complex referring to the DENV-2 protease (PDB 2FOM - black line) and the control DNTB (blue line) presented greater instability, as it presented fluctuations that culminated in higher RMSD values, which corresponded to 0.47 nm in a time of 50 ns, while compounds **2e** (red line) and **2f** (green line) showed greater stability. Compound **2e** (red line) presented a greater number of contacts at the beginning of the simulation corresponding to an RMSD of 0.35 nm until a period of 30 ns, after this period stabilization of this complex occurred corresponding to values of up to 0.3 nm. Compound **2f** (green line) showed high stability during the entire experiment, with its RMSD values corresponding to 0.3 nm. The stability of the Dengue virus protease (PDB: 2FOM) is essential to maintain the compounds in the active site.

**Figure 11:**
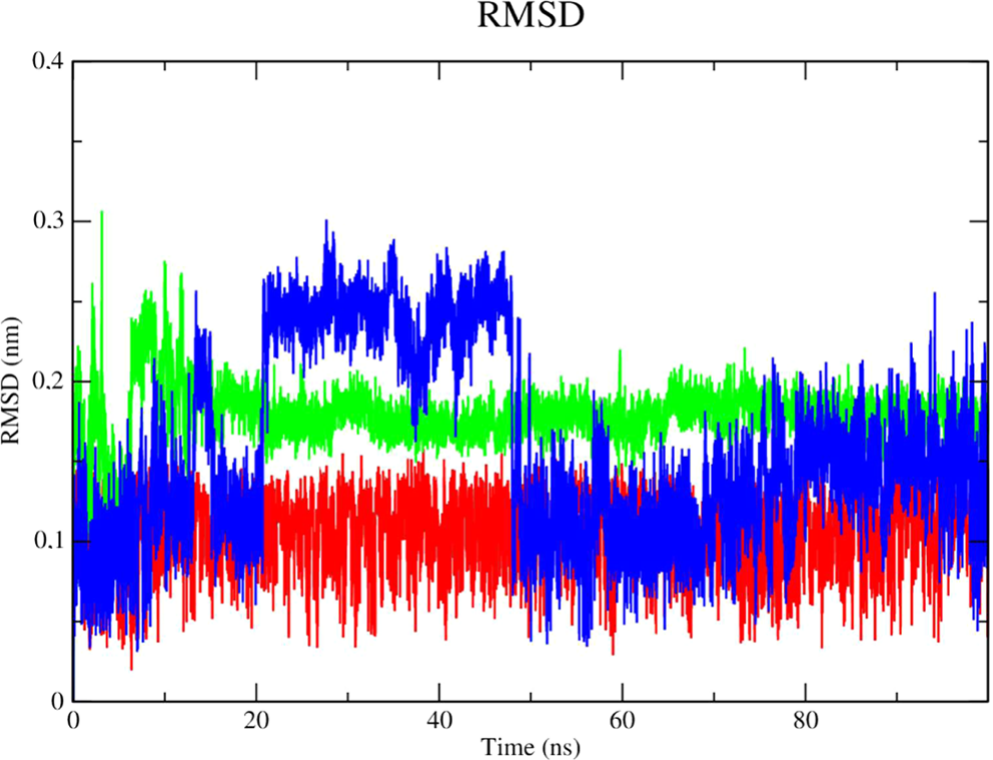
RMSD of the Cα atoms of the compounds. (A) **2e** (red line), **2f** (green line) and the control DNTB (blue line).

Stability analysis of the ligands in the presence of solvents (Figure 11), was performed with compound **2e** (red line). It presented RMSD values lower than the results obtained for compound **2f** (green line) and the control DNTB (blue line), DNTB was more unstable at the beginning of the simulation (time of 15 and 47 ns, respectively) with RMSD values of up to 0.3 nm. Compound **2e** was able to form robust interactions with the protease at the allosteric site despite the presence of solvents, ions, and other elements. The fact that compound **2e** contains a shorter carbon chain than compound **2f** can justify this outcome. Therefore, compound **2e** was set up as a low-degree-of-freedom molecular system in terms of Cartesian atomic coordinates, which makes intricate movement more difficult but also lessens structural overlap.^48^

**Figure 12:**
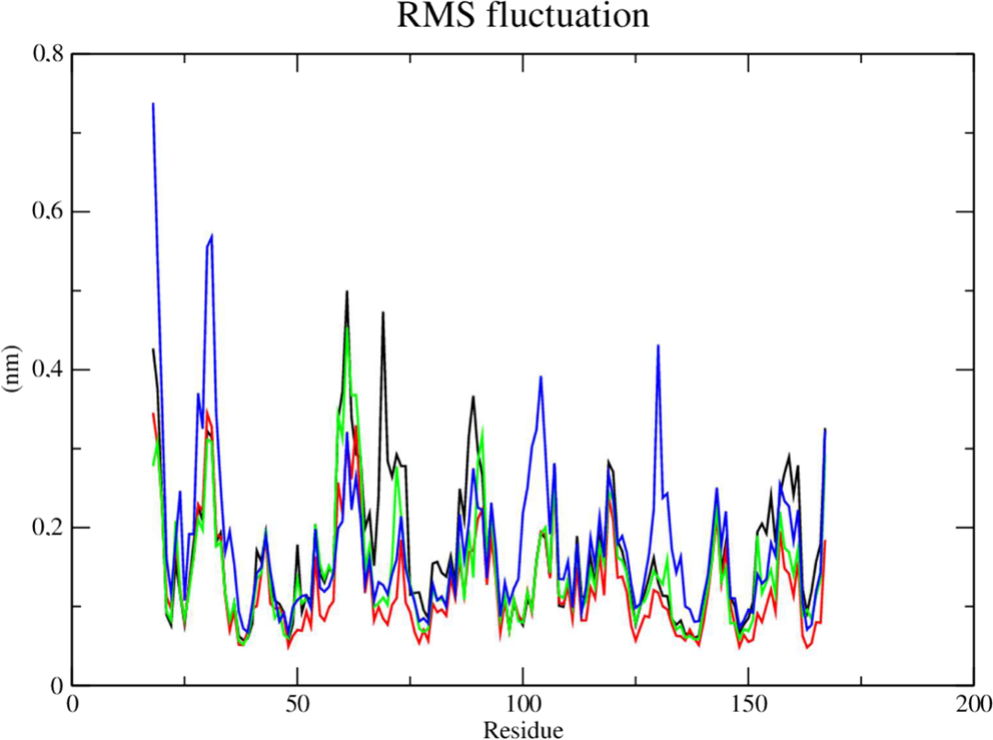
RMSF of atoms. (A) of the Dengue protease enzyme (PDB: 2FOM) (black line) complexed to compounds **2e** (red line), **2f** (green line) and DNTB (blue line).

The root-mean-square fluctuations (RMSF) of each amino acid in the protein were computed in order to comprehend the flexibility of residues and amino acids that contribute to the conformational shift in Dengue protease (PDB: 2FOM). Residuals with high RMSF values represent more flexibility while low RMSF values reflect less flexibility. It was discovered that among the amino acids present in the protein, residues at positions 19, 30, 31, and 61 contribute to the conformational change of the protein complexed with compounds **2e** (red line) and **2f** (green line), taking into account that amino acids with fluctuations above 0.3 nm contribute to the flexibility of the channel structure (Figure 12). Although the aforementioned residues are not part of the protein’s active site, they help compounds **2e** (red line) and **2f** (green line) to stay complexed with the protein.

### Dengue Protease Subjected to Open Site Mutation (PDB ID 2FOM)

**Figure 13:**
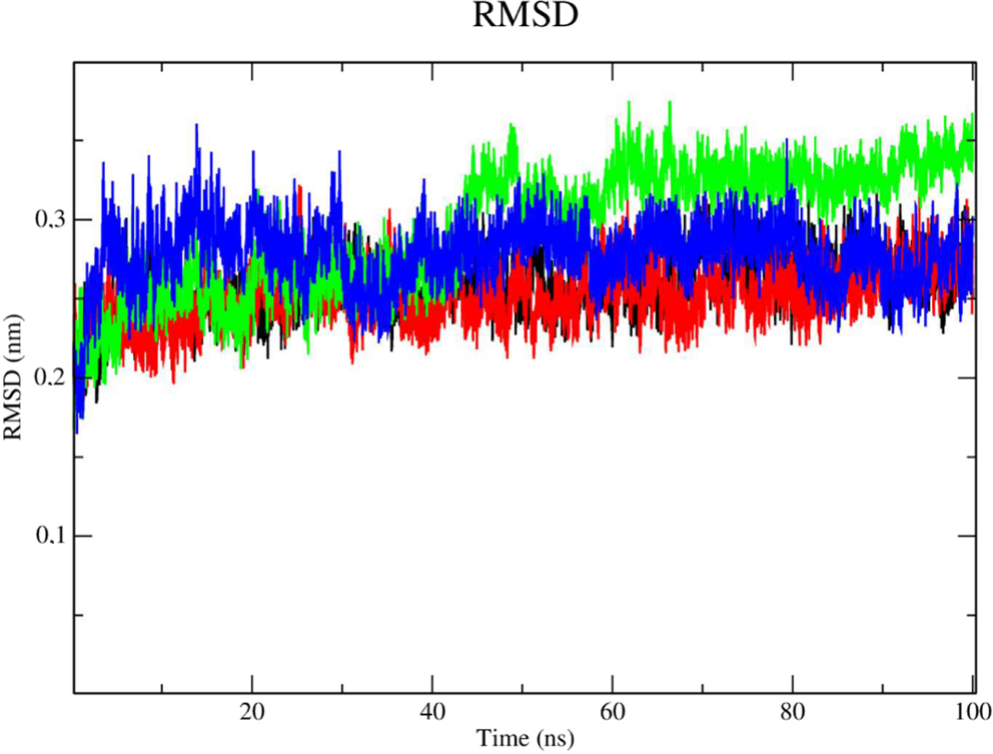
RMSD of Cα atoms. (A) of the Dengue protease in mutant form (PDB: 2FOM) (black line) and bound to compounds **2e** (red line), **2f** (green line) and BMI (blue line).

The mutation in DENV-2 NS2B/NS3 (PDB: 2FOM) confers more stability to the complex (black line – Figure 13) when compared to the nonmutant enzyme. Compound **2e** (red line) showed high stability and no fluctuations in RMSD values but also presented lower RMSD values when compared to compound **2f** (green line) and the control, *N*-benzylmaleimide (blue line). Compound **2f** (green line) showed stability until 40 ns with RMSD values of 0.3 nm. Beyond this period, it showed signs of instability, fluctuating between 0.35 and 0.38 nm in RMSD values. BMI showed high fluctuations during the simulation, mainly during the period from 0 to 40 ns, in which RMSD values were up to 0.37 nm.

**Figure 14:**
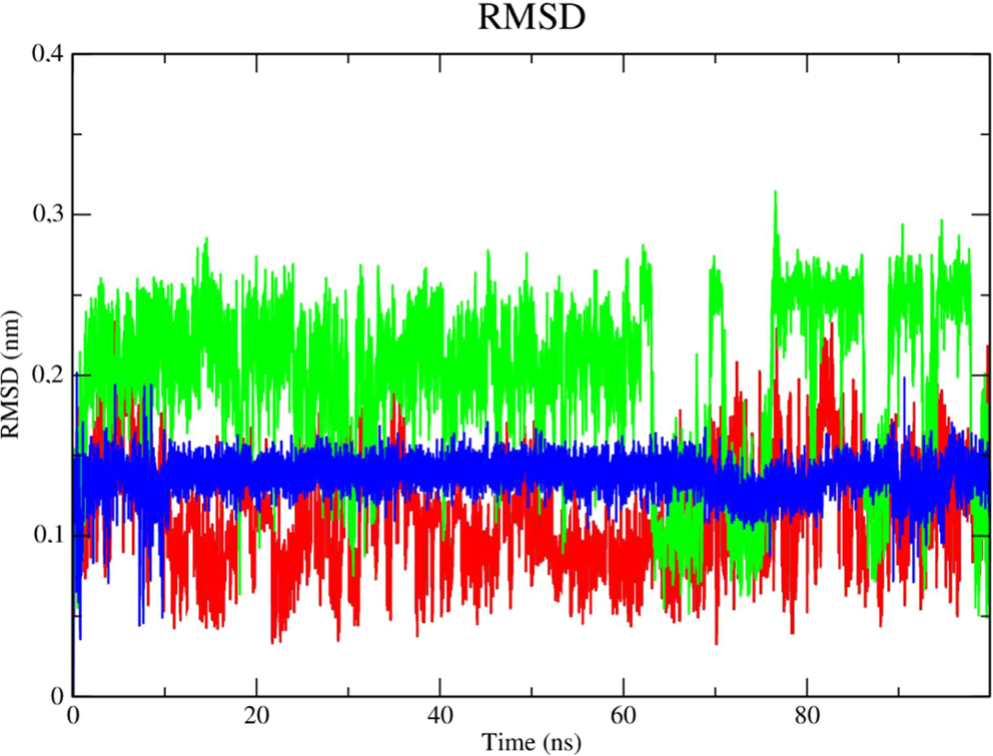
RMSD of the Cα atoms of compounds. (A) **2e** (red line), **2f** (green line) and *N*-benzylmaleimide (blue line).

Evaluation of the stability of the complexes under the influence of solvents, pressure, and temperature, revealed that compound **2f** (green line) presented instability with high RMSD values (Figure 14). According to this result, compound **2e** (red line) and BMI (blue line) have the highest likelihood of staying in the active site even when various conditions including temperature, pressure, solvent, and ions were applied.

**Figure 15:**
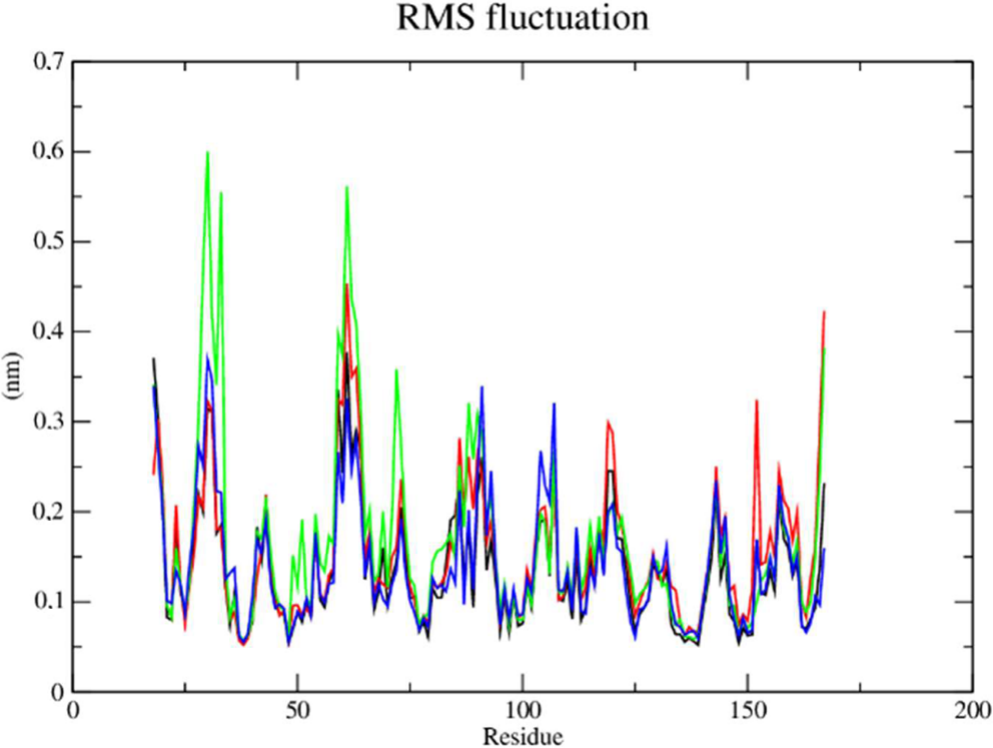
RMSF of atoms. (A) of the Dengue protease in mutant form (PDB: 2FOM) (black line) complexed with compounds **2e** (red line), **2f** (green line) and BMI (blue line).

The conformational shift of the protein complexed with compounds **2e** and **2f** is attributed to amino acid residues located at positions 31 and 61 in the protein (Figure 15). However, these residues are not part of the active site of the protein but enabled compounds **2e** and **2f** to remain attached to the protein.

### Docking Result for Zika Virus Protease

**Figure 16:**
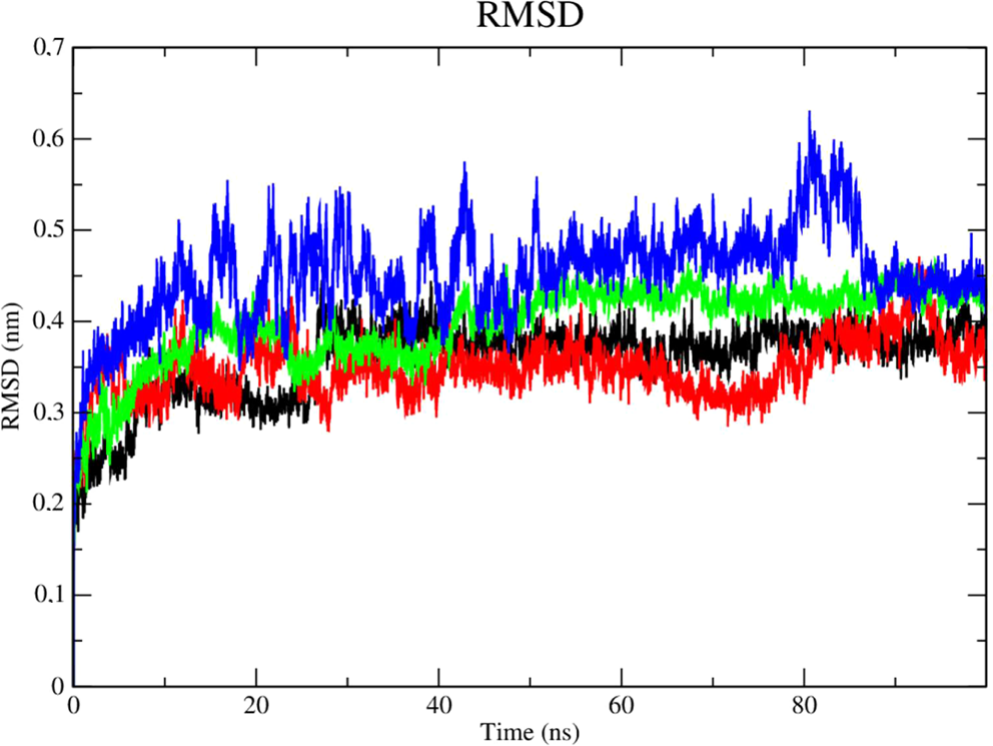
RMSD of Cα atoms. (A) Zika virus protease enzyme in mutant form (PDB: 7VLG) (black line) complexed to compounds **2f** (red line), **2g** (green line) and PDB ligand (blue line).

The complex corresponding to ZIKV NS2B/NS3 (PDB 7LVG - black line) demonstrated higher stability, consistent with low RMSD values (corresponding to 3.0 nm throughout the simulation), according to an analysis of the protein’s RMSD metric (Figure 16). The PDB ligand presented the greatest instability, as it presented higher RMSD values, which corresponded to 0.55 nm in the 20 ns period and 0.6 nm in the 80 ns period. Compound **2f** demonstrated stability from the 50 ns period onward, with RMSD values that corresponded to 0.45 nm until the end of the simulation. The stability of ZIKV protease (PDB: 7LVG) is important to retain molecules attached to the active site.

**Figure 17:**
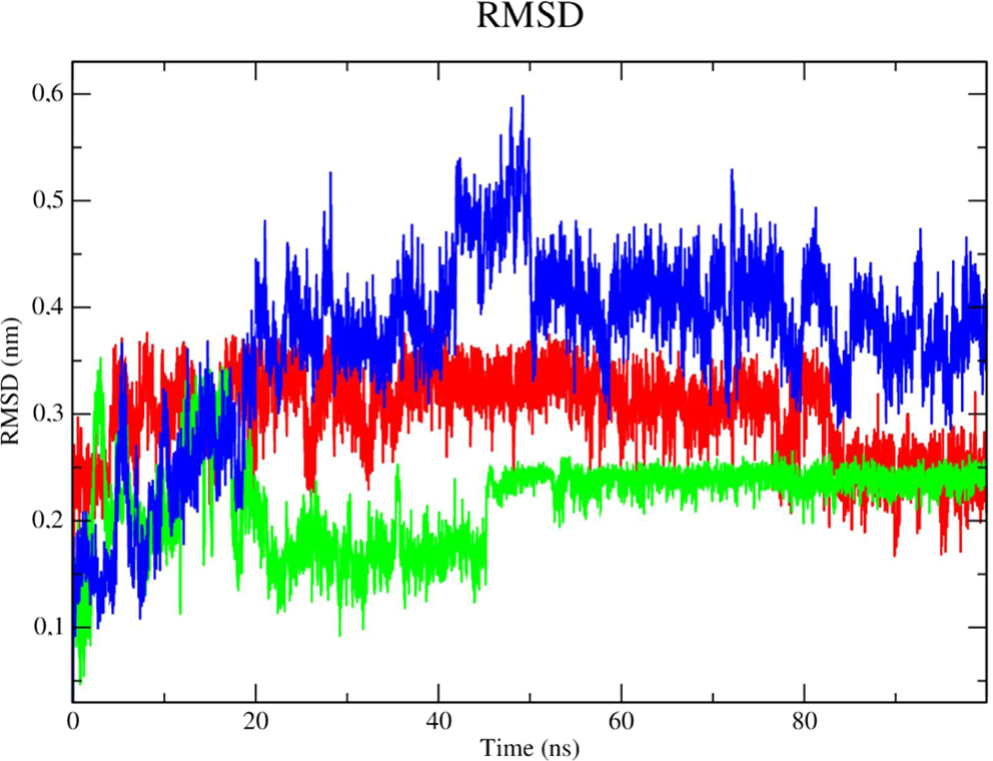
RMSD of the Cα atoms of the compounds. **2f** (red line), **2g** (green line) and PDB ligand (blue line).

The PDB ligand (blue line), as indicated by its high RMSD values, was found to be significantly unstable when the stability of the ligands was examined in the presence of solvents (Figure 17). Given its lower RMSD values, the complex of compound **2g** (green line) and the enzyme was found to be stable in the presence of solvents, ions, and other variables.

**Figure 18:**
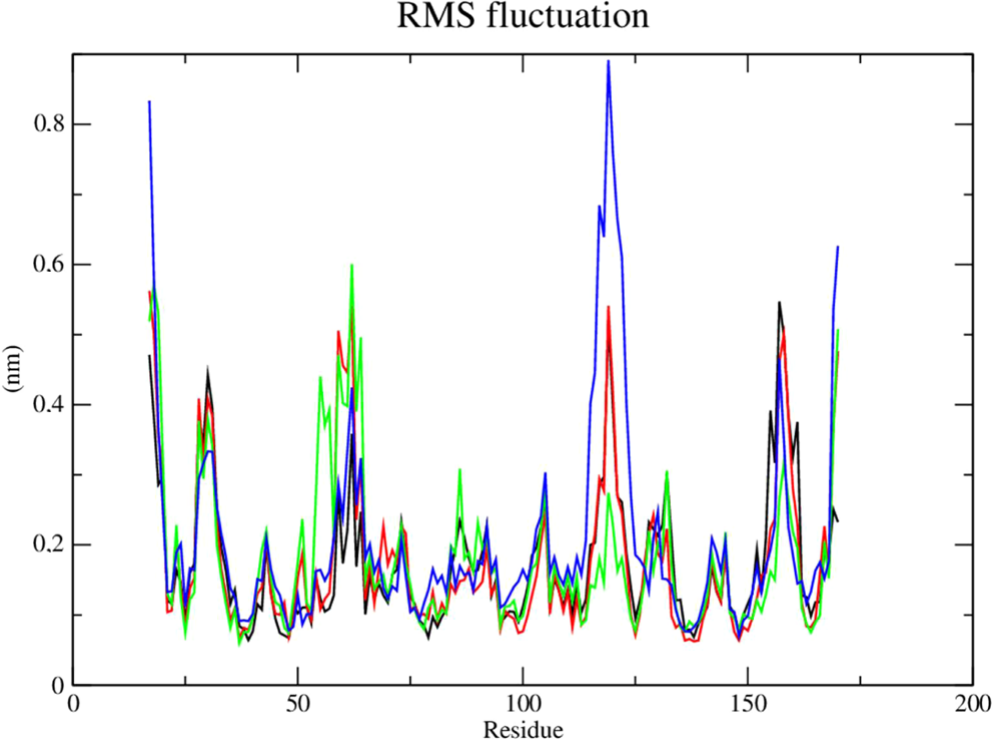
RMSF of atoms. Zika virus protease enzyme (PDB: 7VLG) (black line) complexed to compounds **2f** (red line), **2g** (green line) and allosteric substrate PDB ligand (blue line).

Based on the RMSF metric, it was found that the protein complexed with **2f** and **2g** had a conformational shift that was influenced by amino acid residues at positions 17–18, 30, and 62 (Figure 18). It is crucial to note, nevertheless, that these residues do not form part of the active site and have instead helped to keep both compounds complexed with the enzyme.

## Conclusions

Phytochemical studies of twigs from the mango tree led to the identification of two metabolites namely taraxerol and methyl gallate. As their bioactivity against M^pro^ looked promising, a series of synthetic derivatives was prepared. Because of solubility problems associated with the lipophilicity of the triterpenes, the study was focused on producing gallate esters from gallic acid and alcohols bearing different hydrocarbon chains. The study showed evidence that galloyl moiety is a suitable scaffold to develop substances with potential inhibitory effects against cysteine and serine proteases. Among the compounds which showed in silico, strong probabilities of interactions with these proteases, compounds **2e** and **2i** were the most active against M^pro^ and were more active than the natural product. The biological profiles were different when testing these compounds against NS2B/NS3 from DENV-2 and ZIKV. Compounds **2f** and **2g** were the most active against ZIKV NS2B/NS3 whereas, **2e** and **2f** were the most active against DENV-2 NS2B/NS3. These compounds inhibited the DENV-2 protease by binding to an allosteric pocket and through a noncompetitive mechanism. The same mechanism of action was indirectly found with ZIKV protease. Molecular docking revealed that the galloyl moiety was responsible for most of the hydrogen bond interactions between active compounds with amino acid residues of the evaluated targets.

## Supporting Information Available

NMR (^1^H and ^13^C), IR, and MS data (PDF)
