# Supplementary material for: Inhibitory Effects of Mangifera indica Secondary Metabolites and Their Synthetic Derivatives against SARS-CoV-2 Mpro and NS2B/NS3 (ZIKV and DENV-2)
Source: ACS Omega. 2024 Oct 24;9(44):44624–38. doi: 10.1021/acsomega.4c07148 (PMC11541513; doi:10.1021/acsomega.4c07148)
Supplement: Supplementary file 1 — ao4c07148_si_001.pdf [file ao4c07148_si_001.pdf]

**Inhibitory effects of *Mangifera indica* secondary metabolites and their synthetic derivatives against SARS-CoV-2 $M^{pro}$ and NS2B/NS3 (ZIKV and DENV-2).**

Gabriella B. Souza,[a] Carime L. M. Pontes,[a] Geovanna de O. Costa,[a,b] Natália F. de Sousa,[c] Tiago Tizziani,[a] Luiz Antonio E. Pollo,[d] Bibiana P. Dambrós,[e] Marcus T. Scotti,[c] Mario Steindel,[e] Antonio L. Braga,[a] Tanja Schirmeister,[b] Francisco F. de Assis,[a] and Louis P. Sandjo[a,*]

a Programa de Pósgraduação em Química, Department of Chemistry, CFM, Universidade Federal de Santa Catarina, 88040-900, Florianópolis, SC, Brazil

b Johannes Gutenberg Universität Mainz, Institute of Pharmacy and Biochemistry Staudingerweg 5 Mainz, DE 55128, Germany.

c Chemistry Department, Exact and Nature Sciences Center, Federal University of Paraiba, Campus I, 58.051-900, João Pessoa, PB, Brazil

d Department of Pharmaceutical Sciences, CCS, Universidade Federal de Santa Catarina, 88040-900, Florianópolis, SC, Brazil

e Department of Microbiology, Immunology and Parasitology, Universidade Federal de Santa Catarina, 88040-900 Florianópolis, SC, Brazil

*Correspondence to Professor Dr Louis P. Sandjo, Department of Chemistry, CFM, Universidade Federal de Santa Catarina, Campus Universitário-Trindade, 88040-900, Florianópolis, SC, Brazil, Email: p.l.sandjo@ufsc.br; Tel: +554837213624

Taraxerol **(1)**: White solid; m.p: 280-282 °C; IR (KBr, $cm^{-1}$): $\nu_{Max}$ 3400, 2840, 1600, 1465, 1375; $^{1}H$ NMR ($CDCl_3$; 200 MHz): δ 5.5 (*dd*, *J*=8,0 Hz e 4,0 Hz, 1H), δ 3.24-3.15 (*m* 1H), δ 1.32-1.64 (m), δ 0,82-1,25 (m); $^{13}C$ NMR ($CDCl_3$, 50 MHz): δ 158.2, δ 117.0, δ 79.2, δ 37.7, δ 27.3, δ 37.7, δ 55.7, δ 18.9, δ 35.3, δ 41.5, δ 48.9, δ 37.9 δ 17.6, δ 37.9, δ 36.8, δ 36.8,δ 41.5, δ 49.5, δ 41.5, δ 30.8, δ 33.8, δ 33.3, δ 28.2, δ 17.6, δ 15.6, δ 26.0 δ 30.8, δ 28.2, δ 33.5, δ 21.5. [1].

Methyl gallate **(2)**: Yellow solid; m.p: 197-200 °C; IR (KBr, $cm^{-1}$): $\nu_{Max}$ 3400, 3100, 1675, 1540, 1000; $^{1}H$ NMR ($CD_3OD$; 200 MHz): δ 7.04 (s, 2H), δ 3.81 (s, 3H); $^{13}C$ NMR (CD3OD, 50 MHz): δ 169.0, δ 146.5, δ 139. 7, δ 121.4, δ 110.0, δ 52.2. Calculated for ($C_8H_9O_5^+$) m/z $[M+H]^+$ = 185,0450, found, 185,0453.[7]

**Semisynthetic compound**

Taraxerone **(1a)**: White solid; m.p: 226-230 °C; Yield: 85%**;** IR (KBr, $cm^{-1}$): $\nu_{Max}$ 2970, 2865, 1680, 1465, 1375; $^{1}H$ NMR (300 MHz, $CDCl_3$) δ 5.56 (*m*, 1H), δ 0.83-2.58 (m); $^{13}C$ NMR (75 MHz, $CDCl_3$): δ 217.7, δ 157.7, δ 117.3, δ 37.8, δ 38.5, δ 48.9, δ 20.1, δ 35.6, δ 38.5, δ 48.9, δ 17.6, δ 37.8, δ 38.5, δ 36.8, δ 35.9, δ 48.9, δ 40.7, δ 30.1, δ 34.3, δ 33.2, δ 26.2, δ 14.9, δ 30.1, δ 36.8, δ 33.5, δ 21.6.

**Synthetic compounds**

Isopropyl Gallate **(2a):** Brown oil ; Yield: 70%; IR (KBr, $cm^{-1}$): $\nu_{Max}$ 3495, 2965, 1690, 1650, 1466, 1310; $^{1}H$ NMR (400 MHz, $CD_3OD$) δ 5.11 (hept, *J* = 6.40 Hz, 1H); δ 7.03 (s, 2H); δ 1.32 (d, *J*= 8.0 Hz, 6H); $^{13}C$ NMR (100 MHz, $CD_3OD$) δ 168.0; δ 146.4; δ 139.6; δ 122.1; δ 109.9; δ 69.1; δ 22.1.Calculated for ($C_{10}H_{13}O_5^+$) m/z $[M+H]^+$= 213.0758; found 213.0513.

Tetradecyl Gallate **(2b):** Brown oil; Yield: 65%; IR (KBr, $cm^{-1}$): $\nu_{Max}$ 3400, 2970, 2850, 1700, 1580, 1250; $^{1}H$ NMR (400 MHz, $CD_3OD$): δ 7.04 (s, 2H); δ 4.21 (t, *J*= 8.0 Hz and *J*= 4.0 Hz, 2H), δ 1.28-1.73 (m, 27H), δ 0.90 (t, *J*= 4.0 Hz and *J*= 8.0 Hz, 3H); $^{13}C$ NMR (100 MHz, $CD_3OD$): δ 168.6; δ146.6, δ 109.9, δ 65.7, δ

33.0, δ 30.7; δ 30.4; δ 29.9; δ 26.7, δ 23.7, δ14.4. Calculated for ($C_{21}H_{35}O_5^+$) m/z $[M+H]^+$ = 367. 2479. found, 367.2505.

But-3-enyl Gallate **(2c)**: Brown oil; Yield: 90%; IR (KBr, $cm^{-1}$): $\nu_{Max}$ 3450, 2950, 1690, 1650, 1580, 1300, 1000; $^1H$ NMR (300 MHz, $CD_3OD$): δ 7.05 (s, 2H); δ 5.88 *(m,* 1H); δ 5.12 (*dd*, *J* = 27.0 Hz, *J*= 18.0 Hz, 2H), δ 4.26 (t, *J* = 9.0 Hz, 2H), δ 2.47 (q, *J* = 6.0 Hz, 2H). $^{13}C$ NMR (75 MHz, $CD_3OD$): δ 168.4; δ 146.4; δ 139.7; δ 135.5; δ 121.5; δ 117.5; δ 110.0; δ 64.8; δ 34.3. Calculated for ($C_{11}H_{13}O_5^+$) m/z $[M+H]^+$ = 225.0763, found, 225.0772.

Allyl Gallate **(2d):** Brown oil; Yield: 85%; IR (KBr, $cm^{-1}$): $\nu_{Max}$ 3450, 2960, 1690, 1650, 1500, 1300; 1000. $^1H$ NMR (300 MHz, $CD_3OD$): δ 7.07 (s, 2H); δ 6.03 *m*, 1H); δ 5.30 (*dd*, *J* = 18.0 Hz, *J*= 42.0 Hz, 2H), δ 4.72 (d, *J* = 6.0 Hz, 2H), $^{13}C$ NMR (75 MHz, $CD_3OD$).$^{13}C$ NMR (75 MHz, $CD_3OD$): δ 168.0; δ 146.5; δ 139.8; δ 133.9; δ 121.4; δ 117.9; δ 110.0; δ 66.1.Calculated for ($C_{10}H_{11}O_5^+$) m/z $[M+H]^+$ = 211.0606, found, 211.0623.

Isoamyl Gallate **(2e):** Brown oil; Yied: 85%; IR (KBr, $cm^{-1}$): $\nu_{Max}$ 3450, 2950, 2880; 1690, 1650, 1500, 1000; $^1H$ NMR (300 MHz, $CD_3OD$): δ 7.05 (s, 2H); δ 4.26 (t, *J*= 6.0 Hz, 2H); δ 1.78-1.61 (m, 3H); δ 0.97 (d, *J*=6.0 Hz, 6H); $^{13}C$ NMR (75 MHz, $CD_3OD$): δ 168.5; δ 146.4; δ 139.7; δ 121.6; δ 109.9; δ 64.2; δ 38.6; δ 26.4; δ 22.8. Calculated for ($C_{12}H_{17}O_5^+$) m/z $[M+H]^+$= 241.1076, found m/z $[M+H\text{-Isoamyl acetate}]^+$ 241.1086, 171.0297.

Geranyl Gallate **(2f):** Brown oil; Yield: 70%; IR (KBr, $cm^{-1}$): $\nu_{Max}$ 3450; 2960; 1690; 1640; 1310; 1050; $^1H$ NMR (300 MHz, $CD_3OD$): δ 7.06 (s, 2H), δ 5.42 (*sl*, 1H), δ 5.08 (m, 1H), 4.75 (sl, 2H), δ 2.08 (m, 4H), δ 1.75 (sl, 3H), δ 1.64 (sl, 3H); δ 1,58 (s, 3H); $^{13}C$ NMR (75 MHz, $CD_3OD$): δ 168.0; δ146.4, δ 143.3; δ 139.6, δ 132.6, δ 124.8 δ 121.7; δ 119.9, δ 110.0; δ 62.3; δ 40.5; δ 27.3; δ 25.8; δ 17.7; δ16.5. Calculated for ($C_{17}H_{23}O_5^+$) m/z $[M+H]^+$ = 307.1545, found 307.1544

Citronellyl gallate **(2g):** Brown oil; Yield: 75%; IR (KBr, $cm^{-1}$): $\nu_{Max}$ 3450, 2965, 2860, 1690, 1640, 1310, 1050. $^1H$ NMR (400 MHz, $CD_3OD$): δ 7.06 (s, 2H), δ 5.04 (*t, J*=8.0 Hz, 1H), δ 4.25 (d, *J* = 8.0 Hz, 2H), δ 2.00 (m, 2H), δ 1.73 (m,

2H), δ 1.62 (s, 3H), δ 1.56 (s, 3H), δ 1.49 (m, 1H), δ 1.19 (m, 2H).$^{13}$C NMR (100 MHz, $CD_3OD$): δ 168.4; δ 146.3; δ 139.6, δ 132.0, δ 125.5; δ 121.6; δ 110.0; δ 64.0; δ 37.9; δ 36.5; δ 30.5; δ 26.3; δ 25.8; δ 19.8; δ17.7. Calculated for ($C_{17}H_{25}O_5^+$) m/z $[M+H]^+$ = 309.1702, found 309.1717

3-Phenyl-propyl gallate **(2h):** Brown oil; Yield: 80%; IR (KBr, $cm^{-1}$): $\nu_{Max}$ 3450, 3003, 2965, 1700, 1650, 1500, 1050; $^1$H NMR (400 MHz, $CD_3OD$): δ 7.23-7.09 (m, 7H); δ 4.16 (*t, J*= 8.0 Hz, 2H); δ 2.69 (*t, J*=8.0 Hz, 2H), δ 1.96 (q, *J*= 8.0 Hz, 2H); $^{13}$C NMR (100 MHz, $CD_3OD$): δ 168.4; δ 146.3; δ 142.2, δ 139.6; δ 129.3; δ 126.8, δ 121.5; δ 110.0, δ 64.8; δ 33.0; δ 31.4. Calculated for ($C_{16}H_{17}O_5^+$) m/z $[M+H]^+$ = 289.1076 found 289.1080

Linalyl gallate **(2i):** Brown oil; Yield: 73%; IR (KBr, $cm^{-1}$): $\nu_{Max}$ 3450, 2973, 2850, 1690, 1640, 1310, 1050; $^1$H NMR (400 MHz, $CD_3OD$): δ 7.03 (s, 2H), δ 6.05 (*dd,* J=8.0 Hz, *J*=16.0 Hz 1H), δ 5.27 (*d, J*= 16.0 Hz, 1H), δ 5.17-5.06 (m, 2H), δ 2.05 (*sl*, 2H), δ 1.88-1.82 (m, 2H), δ 1.63 (s, 3H), δ 1.57 (s, 3H), δ 1.29 (s, 3H); $^{13}$C NMR (100 MHz, $CD_3OD$): δ 167.3; δ 146.4; δ 143.5; δ 139.5; δ 132.7; δ 124.9; δ 122.9; δ 113.4; δ 109.9; δ 84.2; δ 40.7; δ 25.8; δ 24.6; δ 23.5; δ 17.6. Calculated for ($C_{17}H_{23}O_5^+$) m/z $[M+H]^+$ = 307.1545, found 307.1544.

2-Phenyl-ethyl gallate **(2j):** Brown oil; Yield: 78%; IR (KBr, $cm^{-1}$): $\nu_{Max}$ 3450, 3000, 2965, 1700, 1655, 1465, 1300, 1000; $^1$H NMR (400 MHz, $CD_3OD$): δ 7.04 (s, 2H), δ 7.28-7.16 (m, 5H), δ 4.40 (t, *J*= 4.0 Hz, 2H), δ 3.00 (t, *J*= 4.0 Hz, 2H). $^{13}$C NMR (100 MHz, $CD_3OD$): δ 168.4; δ 146.4, δ 139.7, δ 139.4, δ129.9, δ 129.4, δ 127.4, δ 121.5, δ 110.0, δ 66.4; δ 36.1. Calculated for ($C_{15}H_{15}O_5^+$) m/z $[M+H]^+$ = 275.0919 found 275.0934.

Hex-3-enyl gallate **(2k):** Brown oil; Yield: 65%; IR (KBr, $cm^{-1}$): $\nu_{Max}$ 3450, 3000, 2960, 1700, 1675, 1300, 1000. $^1$H NMR (400 MHz, $CD_3OD$): δ 7.04 (s, 2H), δ 4.22 (t, *J*= 4.0 Hz, 2H), δ 2.48 (*dd, J*= 12.0 Hz*, J*= 8.0 Hz, 2H), δ 5.55-5.37 (m, 2H), δ 2.10 (m, 2H), δ 0.96 (*t, J*=8.0 Hz, 3H). $^{13}$ C NMR (100 MHz, $CD_3OD$): δ 168.5; δ 146.4; δ 135.4; δ 125.1; δ 121.6; δ 110.0; δ 65.2; δ 27.8; δ 21.5; δ 14.6. Calculated for ($C_{13}H_{17}O_5^+$) m/z $[M+H]^+$ = 253.1076, found 253.1067.

Benzoic acid, 3,4,5-trimethoxy-methyl ester **(2l):** Yellow solid; Yield: 20%, m.p: 83 °C; IR (KBr, $cm^{-1}$): $\nu_{Max}$ 3030, 2965, 1740, 1590, 1500, 1450, 1300, 1000; $^1$H

NMR ($CDCl_3$; 200 MHz): δ 7.29 (s, 2H), δ 3.90 (s, 12H); $^{13}C$ NMR ($CDCl_3$, 50 MHz): δ 166.8, δ 153.0; δ 142.3, δ125.2, δ 106.9, δ 61.0, δ 56.3, δ 52.3. Calculated for ($C_{11}H_{15}O_5^+$) m/z $[M+H]^+$ = 227,0914 found, 227,0919.

4-hydroxy-3,5-dimethoxy-methyl ester **(2m)**: Yellow solid; yield: 18%, m.p: 108 °C; IR (KBr, $cm^{-1}$): $\nu_{Max}$ 3490, 3000, 2960, 1700, 1580, 1500, 1450, 1200, 1050; $^1H$ NMR ($CDCl_3$; 200 MHz): δ 7.21 (s, 2H), δ 3.96 (s, 6H); δ 3.87 (s, 3H); $^{13}C$ NMR ($CDCl_3$, 50 MHz): δ 167.0, δ 148.9, δ 138.8, δ125.9, δ 109.8, δ 61.1, δ 52.4. Calculated for ($C_{10}H_{13}O_5^+$) m/z $[M+H]^+$ = 213,0763 found 213,0758.

## IR, NMR ($^1$H and $^{13}$C) and MS spectrum of target compounds

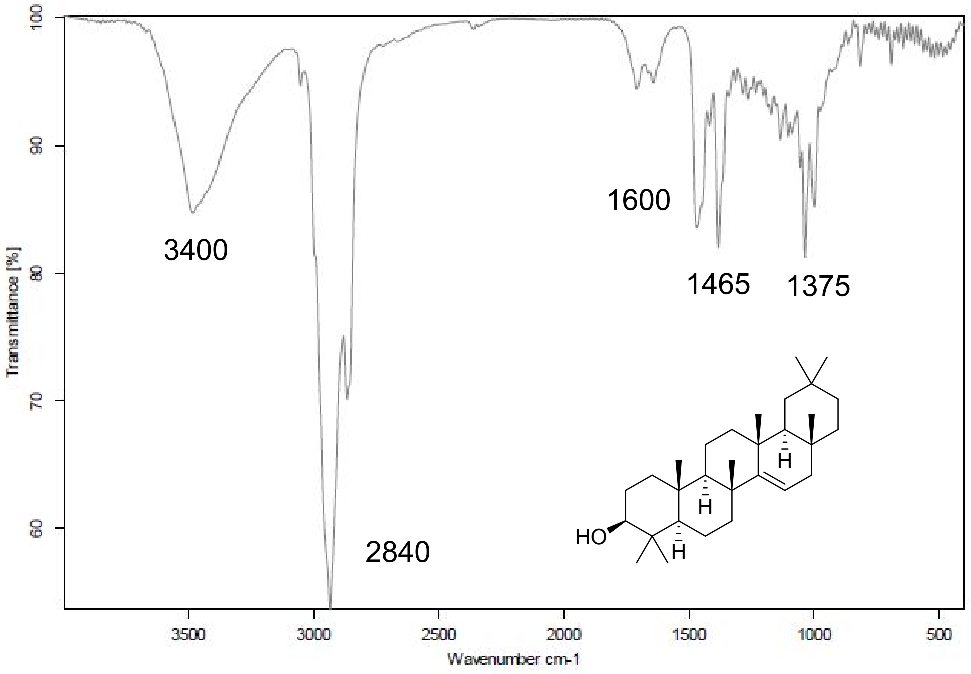


Figure S1: IR spectrum of the compound **Taraxerol**

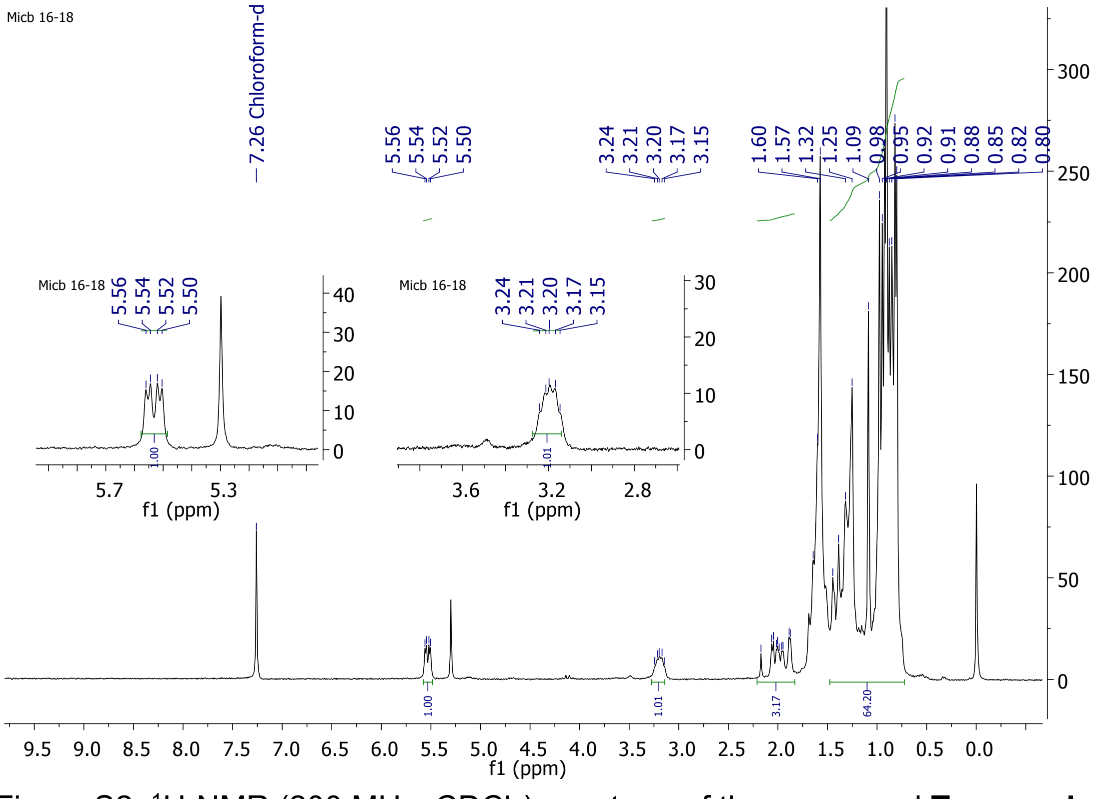


Figure S2: $^1$H-NMR (200 MHz, $CDCl_3$) spectrum of the compound **Taraxerol**

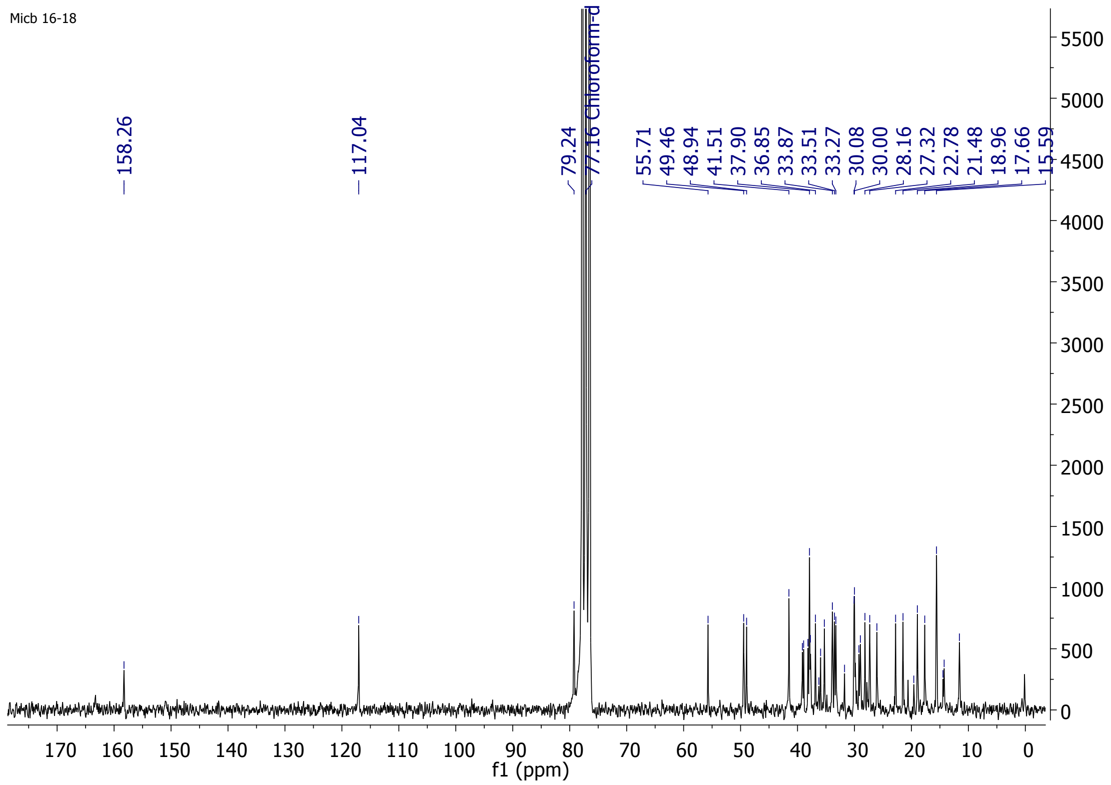


Figure S3: $^{13}$C-NMR (50 MHz, $CDCl_3$) spectrum of the compound **Taraxerol**

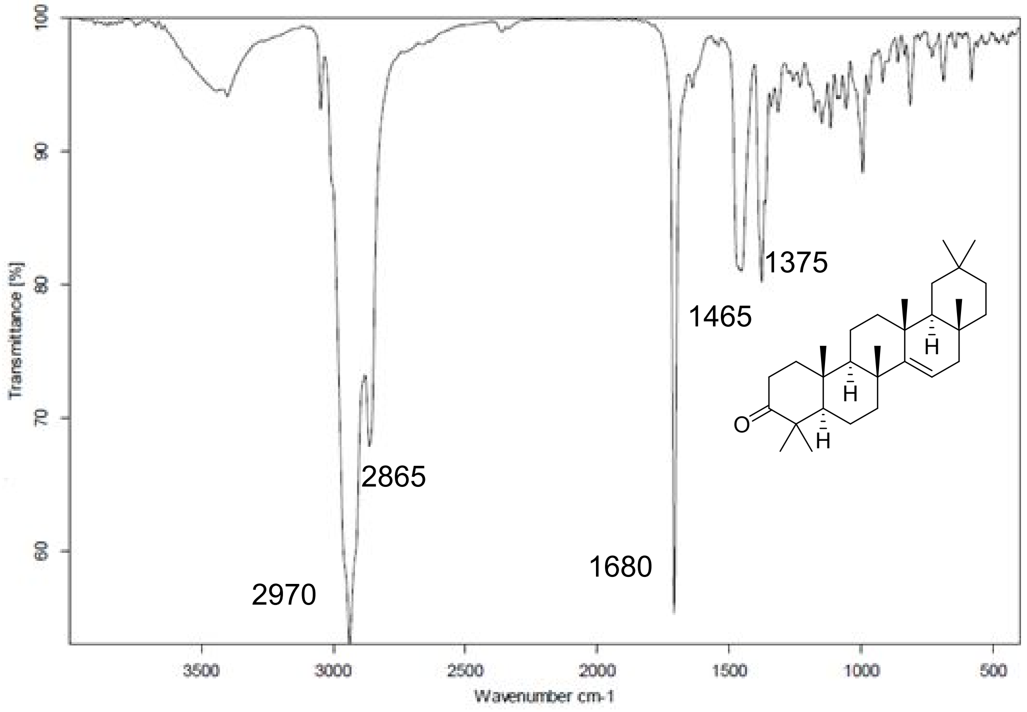


Figure S4: IR spectrum of the compound **Taraxerone**

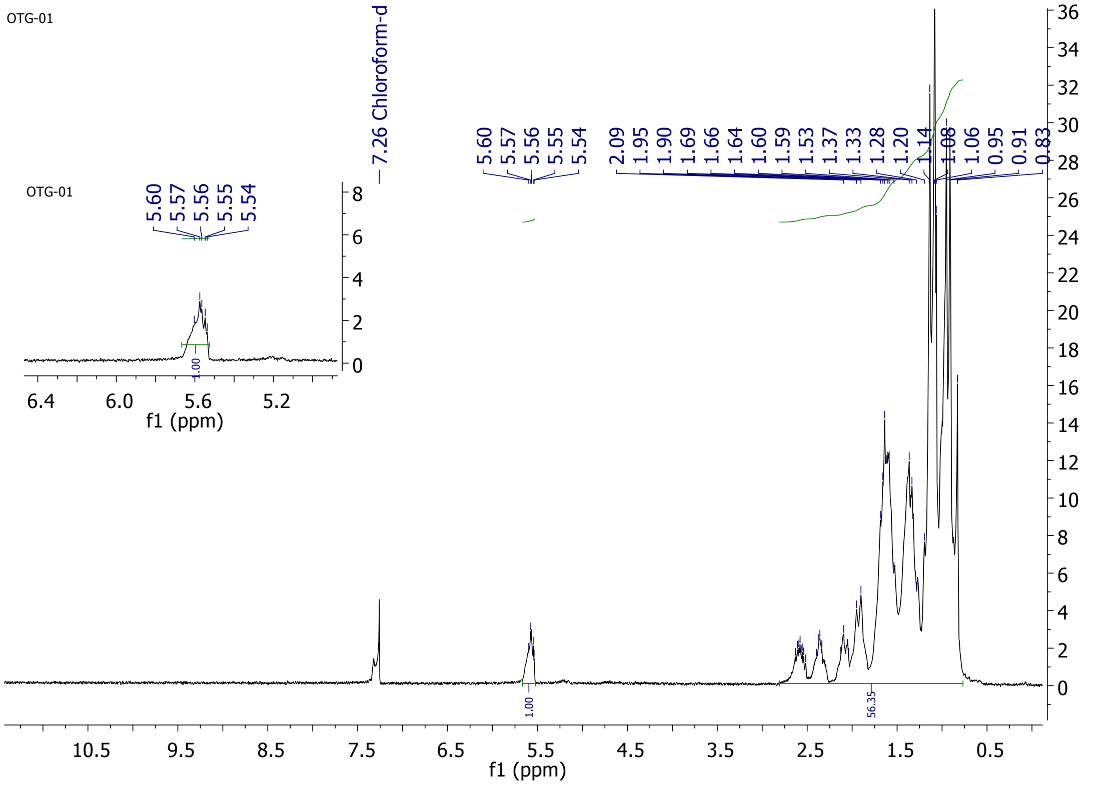


Figure S5: $^{1}H$-NMR (300 MHz, $CDCl_3$) spectrum of the compound **Taraxerone**

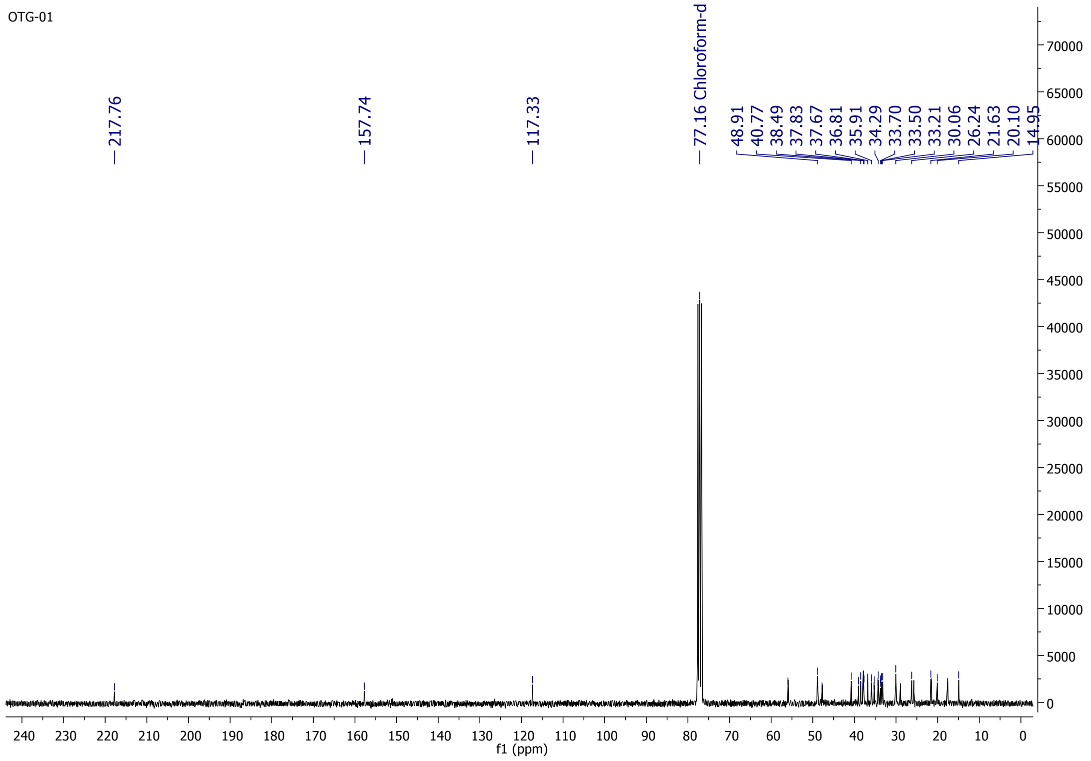


Figure S6: $^{13}C$-NMR (75 MHz, $CDCl_3$) spectrum of the compound **Taraxerone**

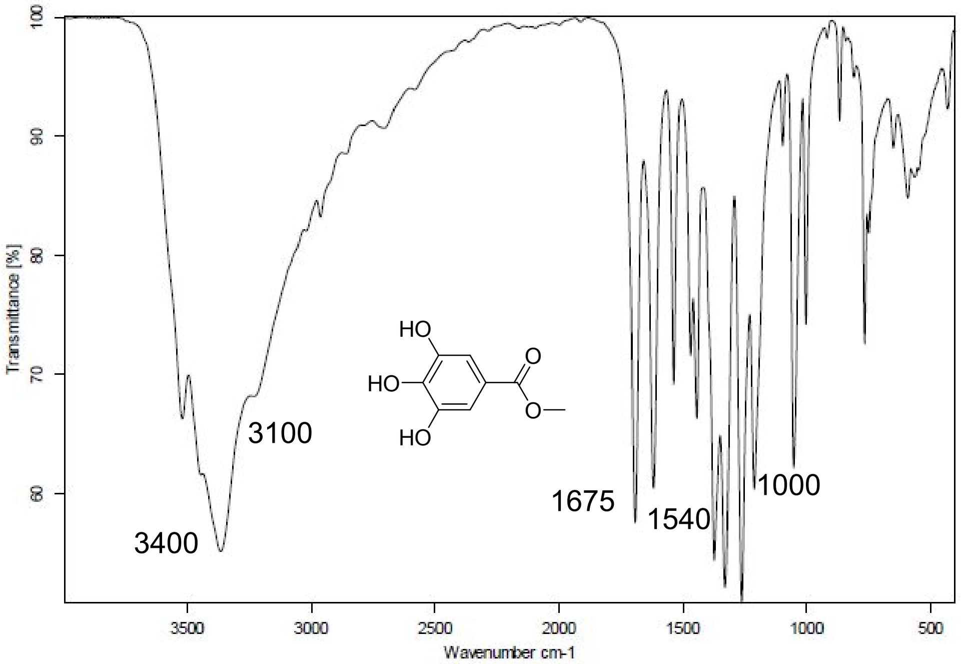


Figure S7: IR spectrum of the compound **Methyl Gallate**

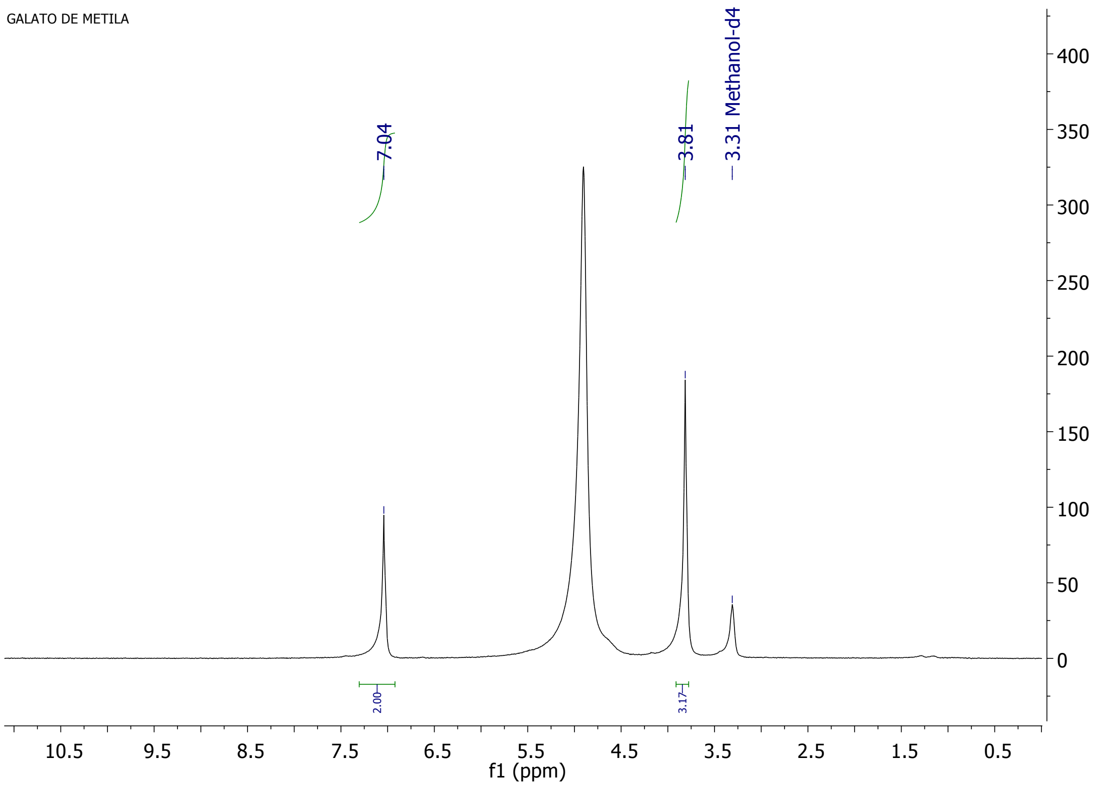


Figure S8: $^{1}$H-NMR (200 MHz, $CD_3OD$) spectrum of the compound **Methyl Gallate**

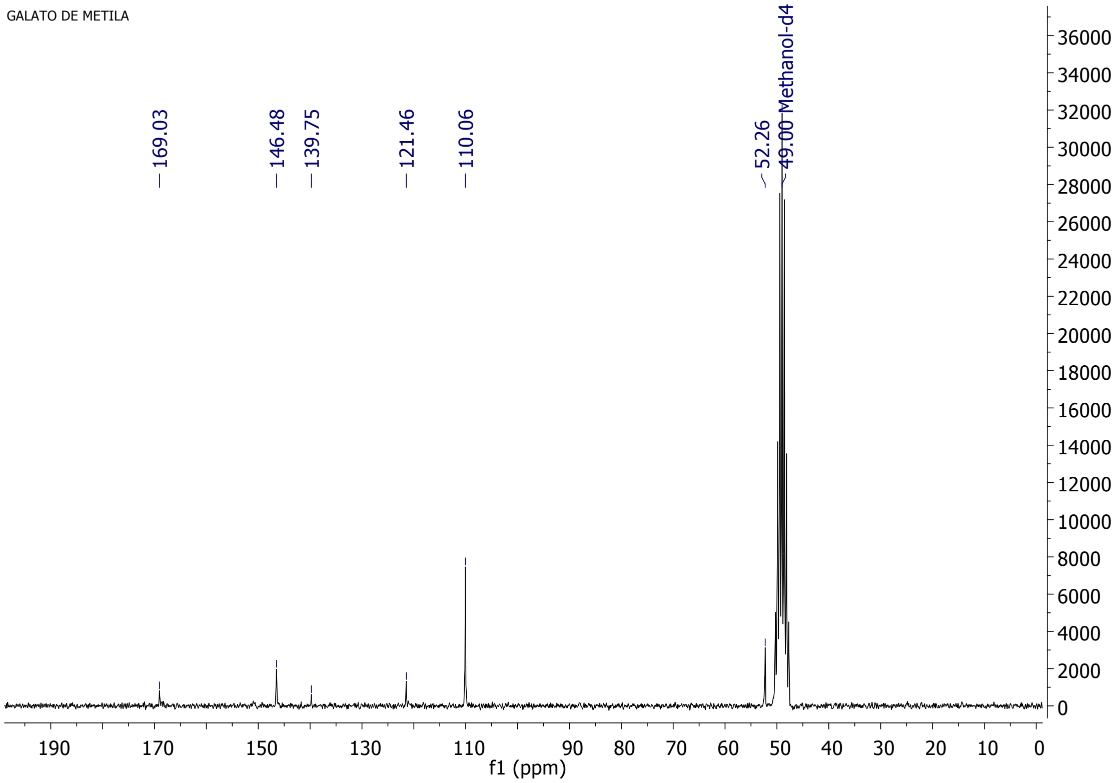


Figure S9: $^{13}$C-NMR (50 MHz, $CD_3OD$) spectrum of the compound **Methyl Gallate**

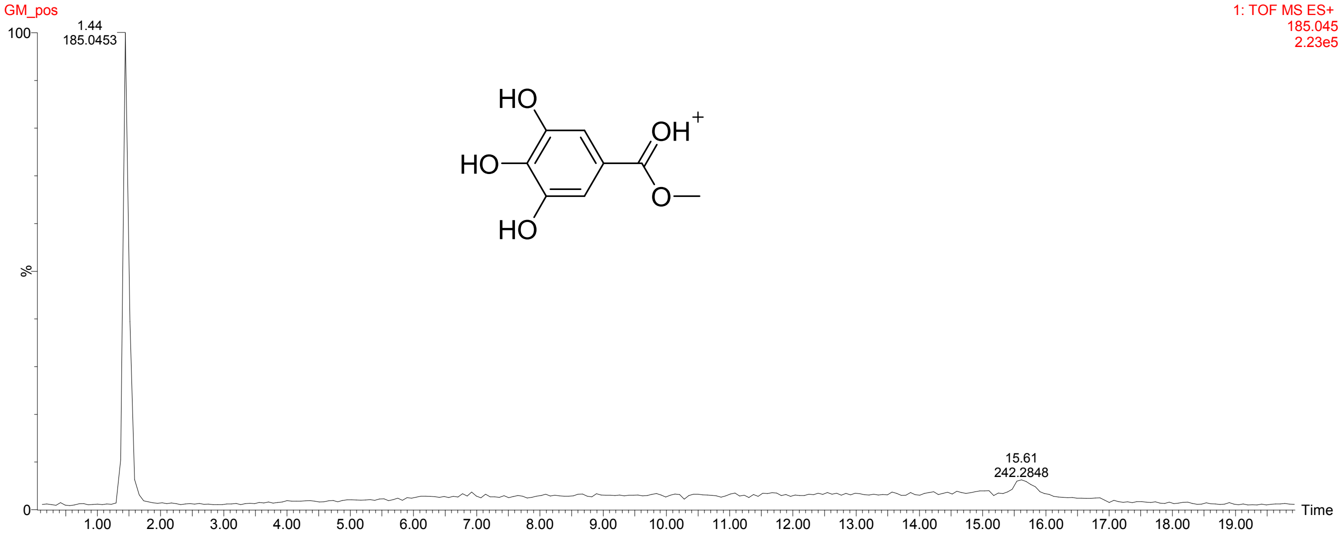


Figure S10: UPLC-ESI-MS of the compound **Methyl Gallate**

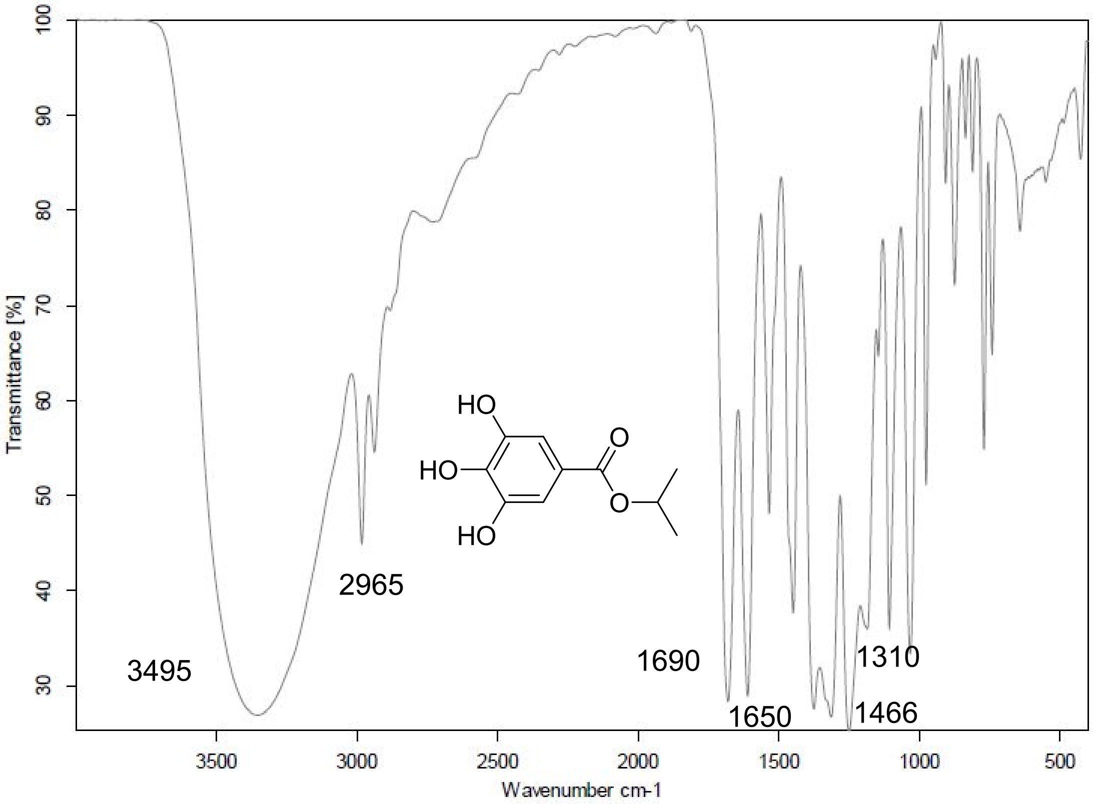

Figure S11: IR spectrum of the compound **Isopropyl Gallate**

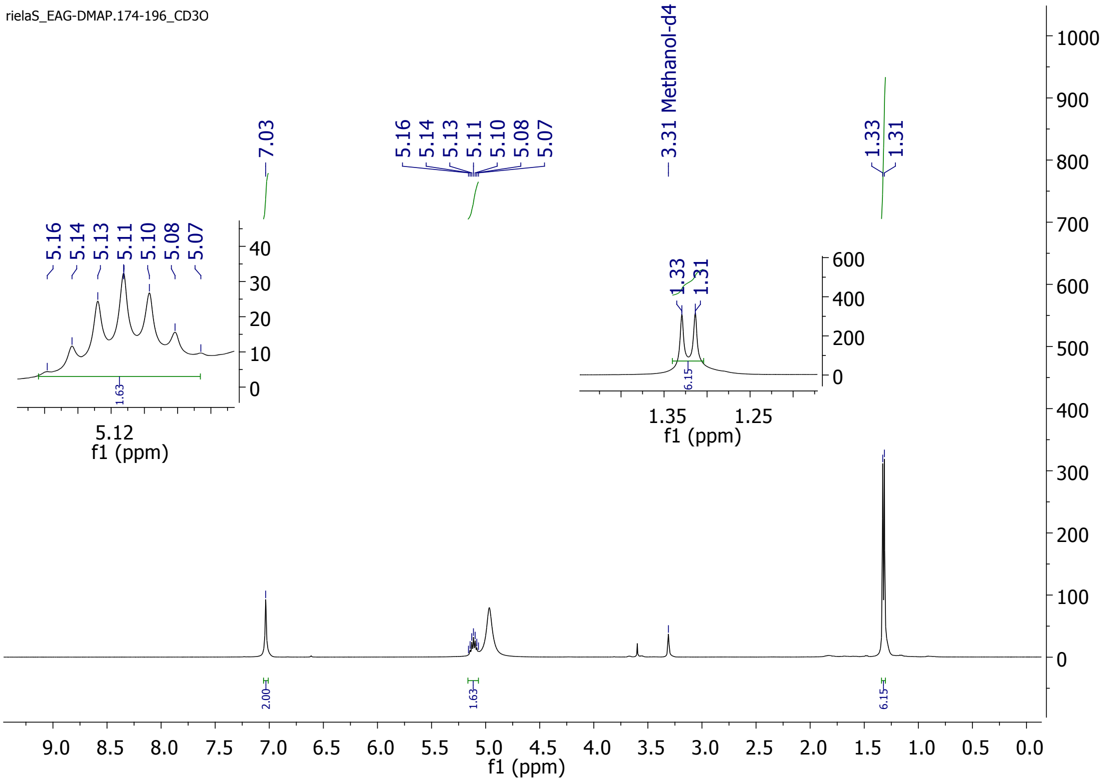

Figure S12: $^{1}$H-NMR (400 MHz, $CD_3OD$) spectrum of the compound **Isopropyl Gallate**

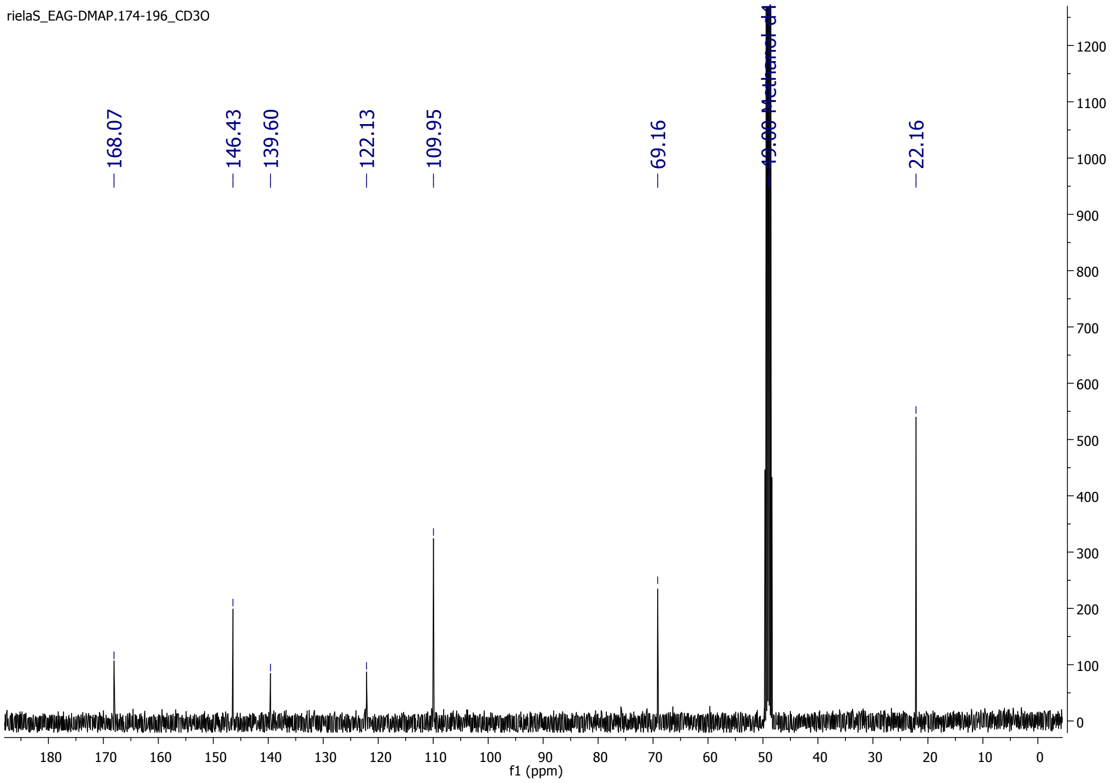


Figure S13: $^{13}$C-NMR (100 MHz, $CD_3OD$) spectrum of the compound **Isopropyl Gallate**

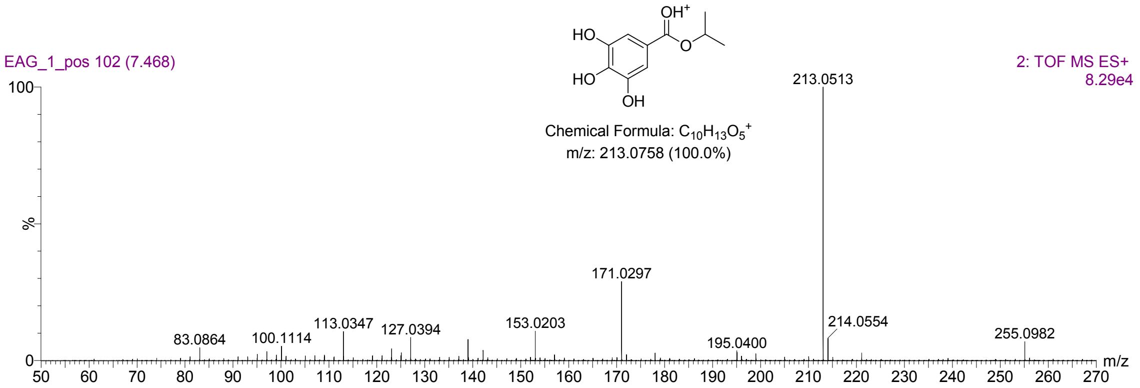


Figure S14: UPLC-ESI-MS of the compound **Isopropyl Gallate**

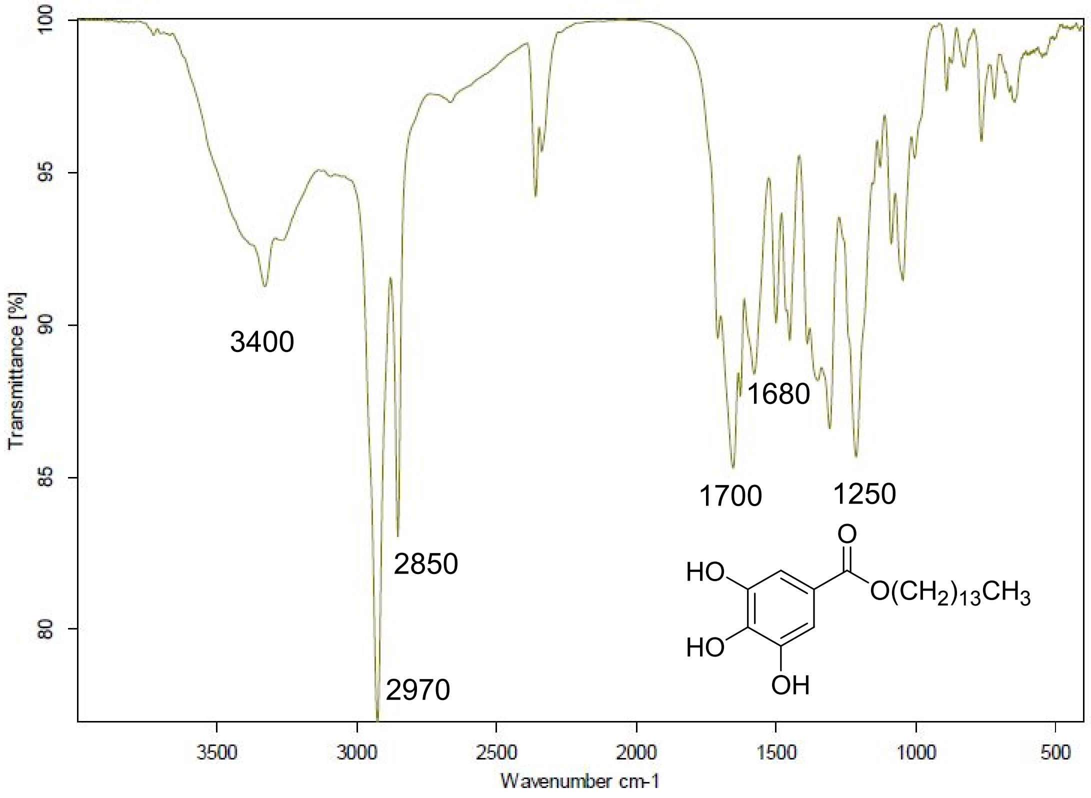


Figure S15: IR spectrum of the compound **Tetradecyl Gallate**

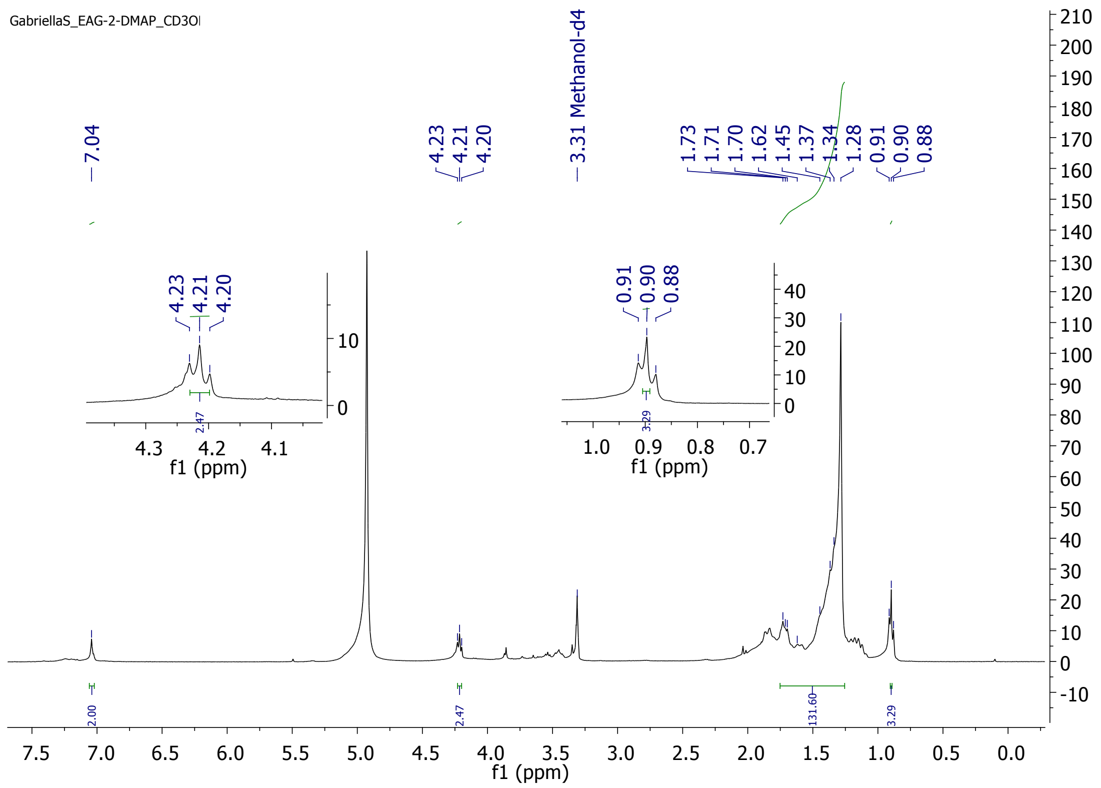


Figure S16: $^{1}$H-NMR (400 MHz, $CD_3OD$) spectrum of the compound **Tetradecyl Gallate**

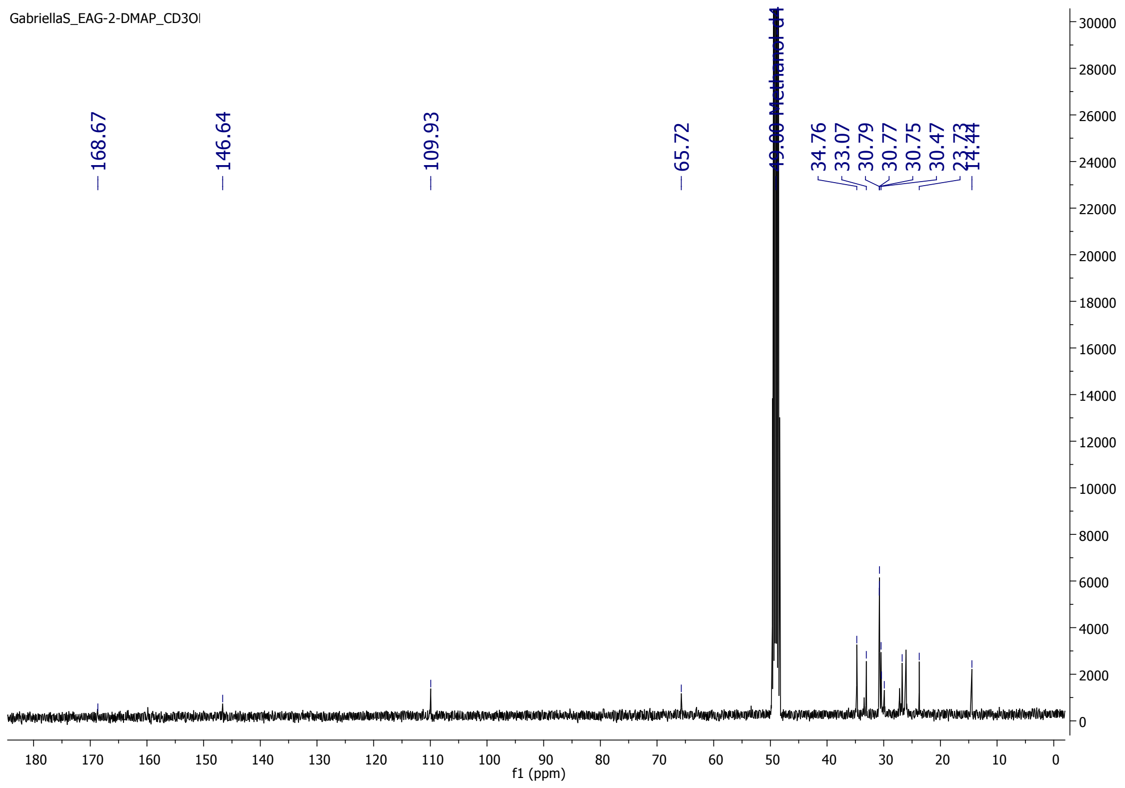


Figure S17: $^{13}$C-NMR (100 MHz, $CD_3OD$) spectrum of the compound **Tetradecyl Gallate**

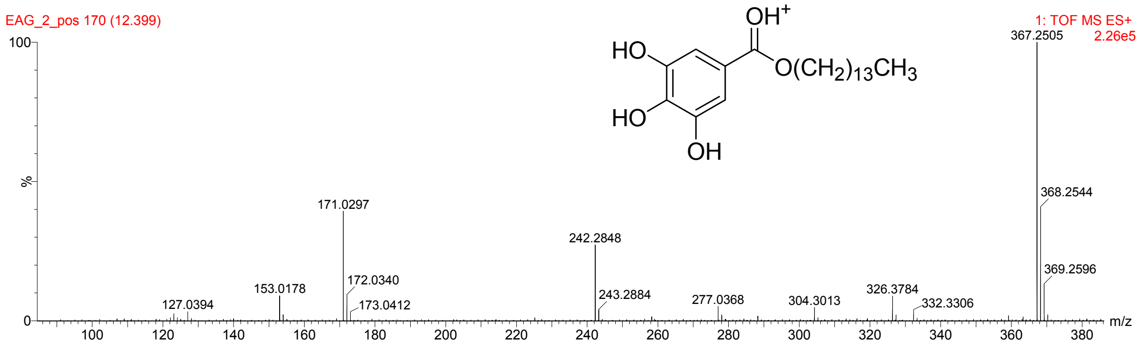


Figure S18: UPLC-ESI-MS of the compound **Tetradecyl Gallate**

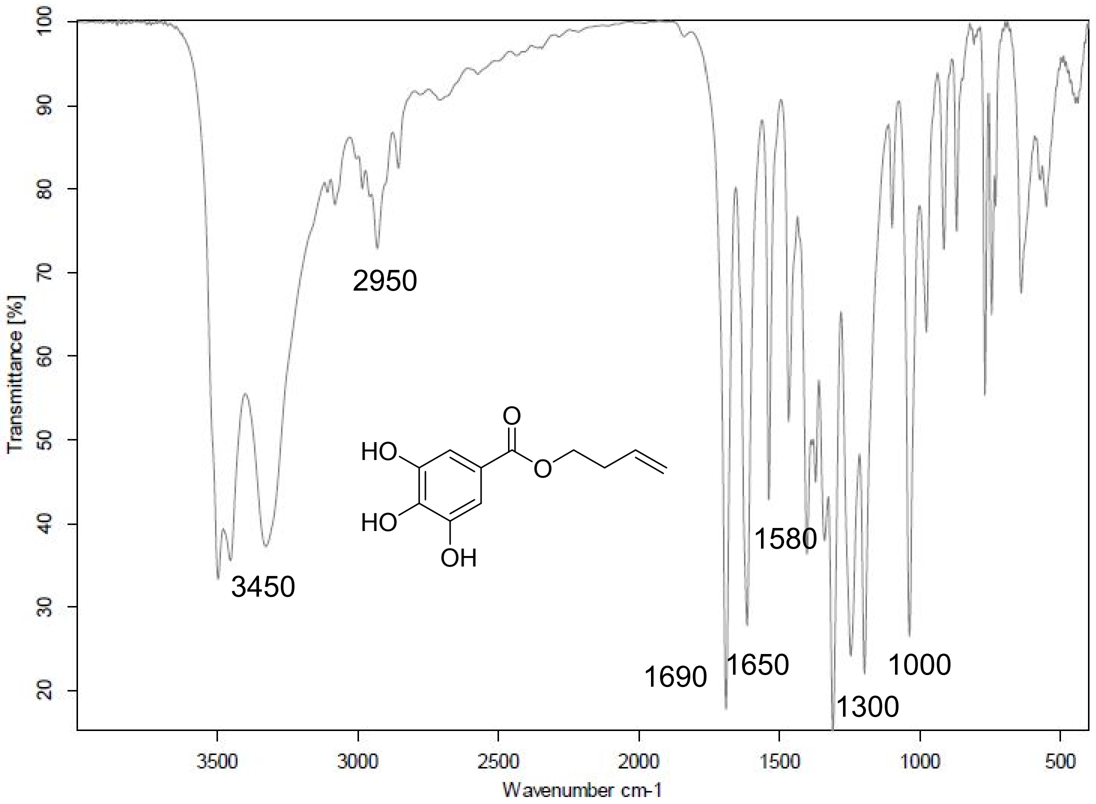


Figure S19: IR spectrum of the compound **But-3-enyl Gallate**

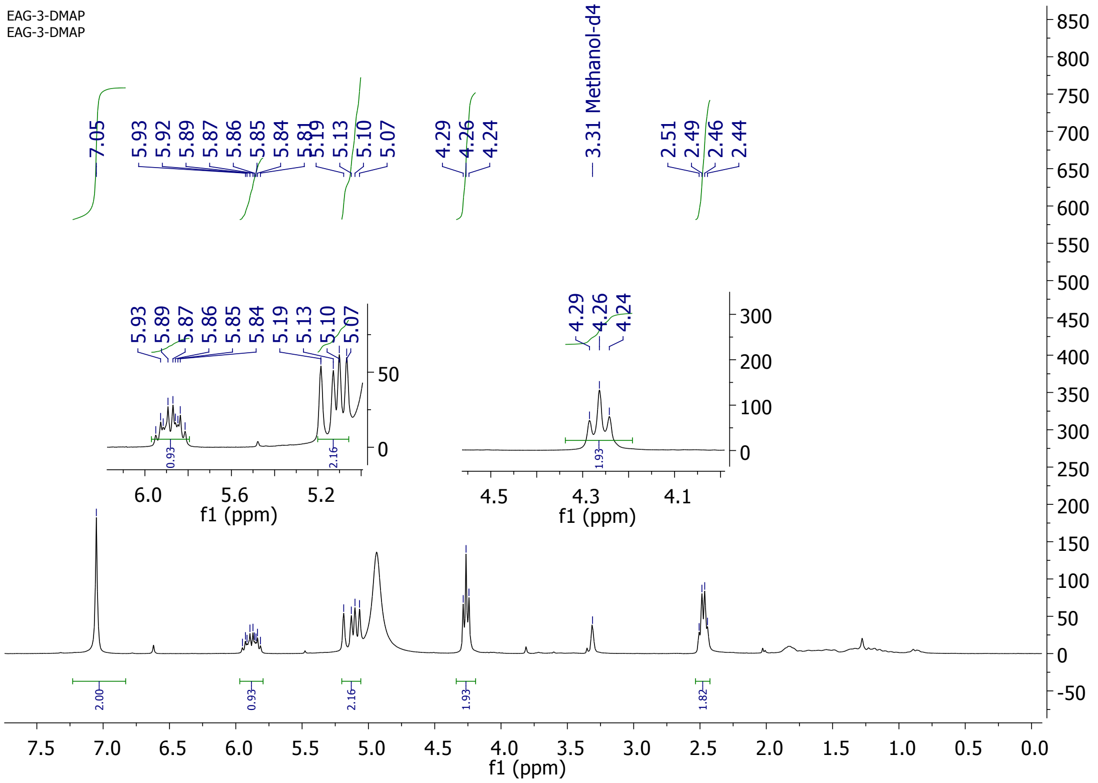


Figure S20: $^{1}$H-NMR (300 MHz, $CD_3OD$) spectrum of the compound **But-3-enyl Gallate**

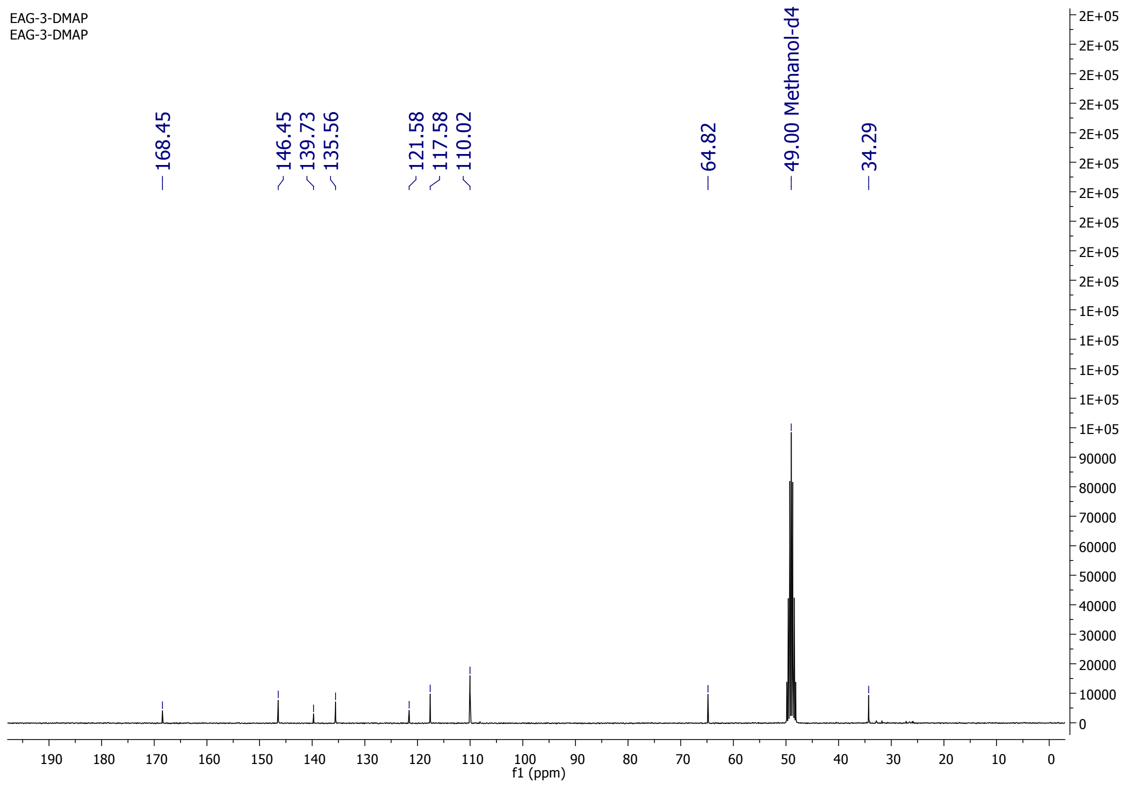


Figure S21: $^{13}C$-NMR (75 MHz, $CD_3OD$) spectrum of the compound **But-3-enyl Gallate**

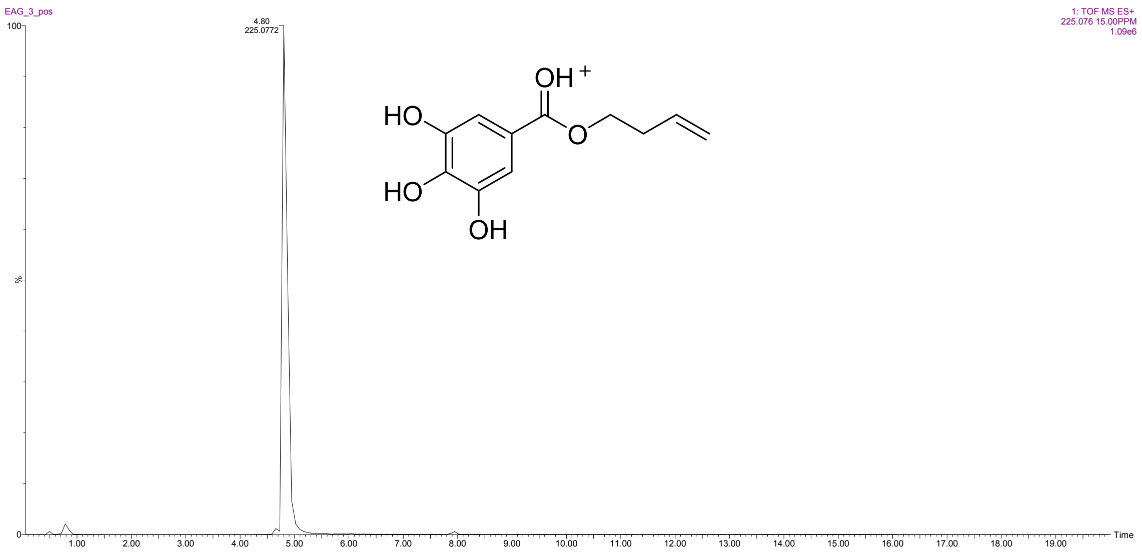


Figure S22: UPLC-ESI-MS of the compound **But-3-enyl Gallate**

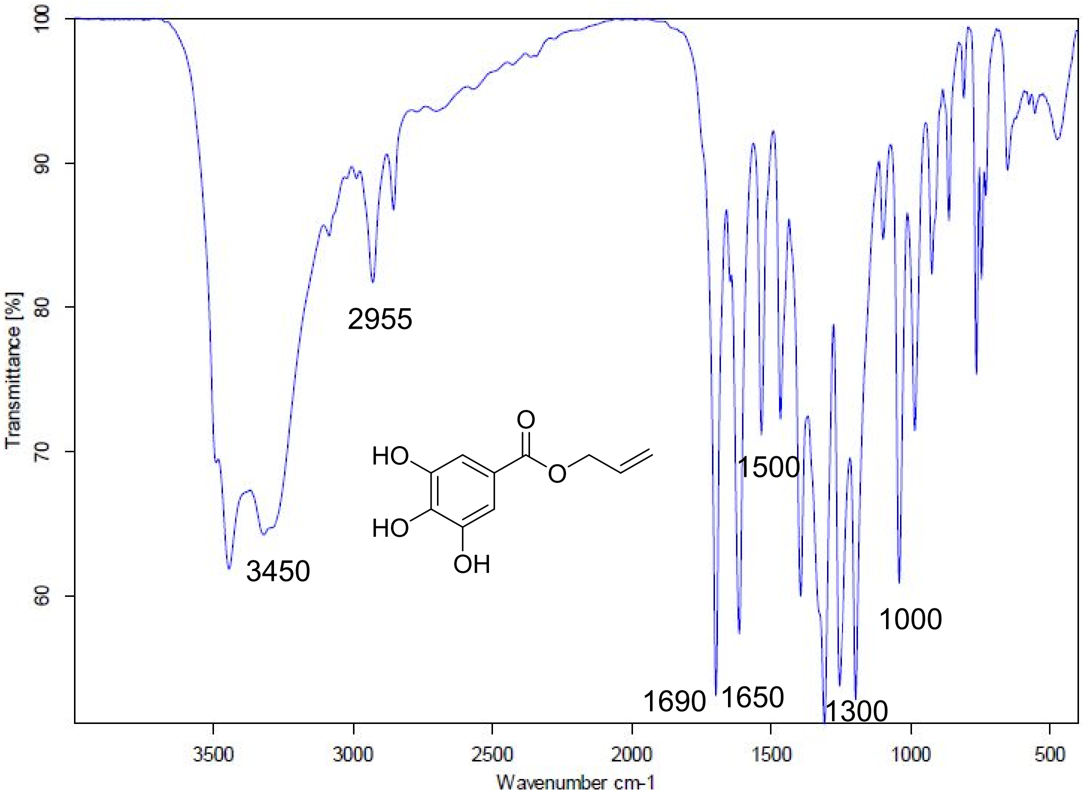


Figure S23: IR spectrum of the compound **Allyl Gallate**

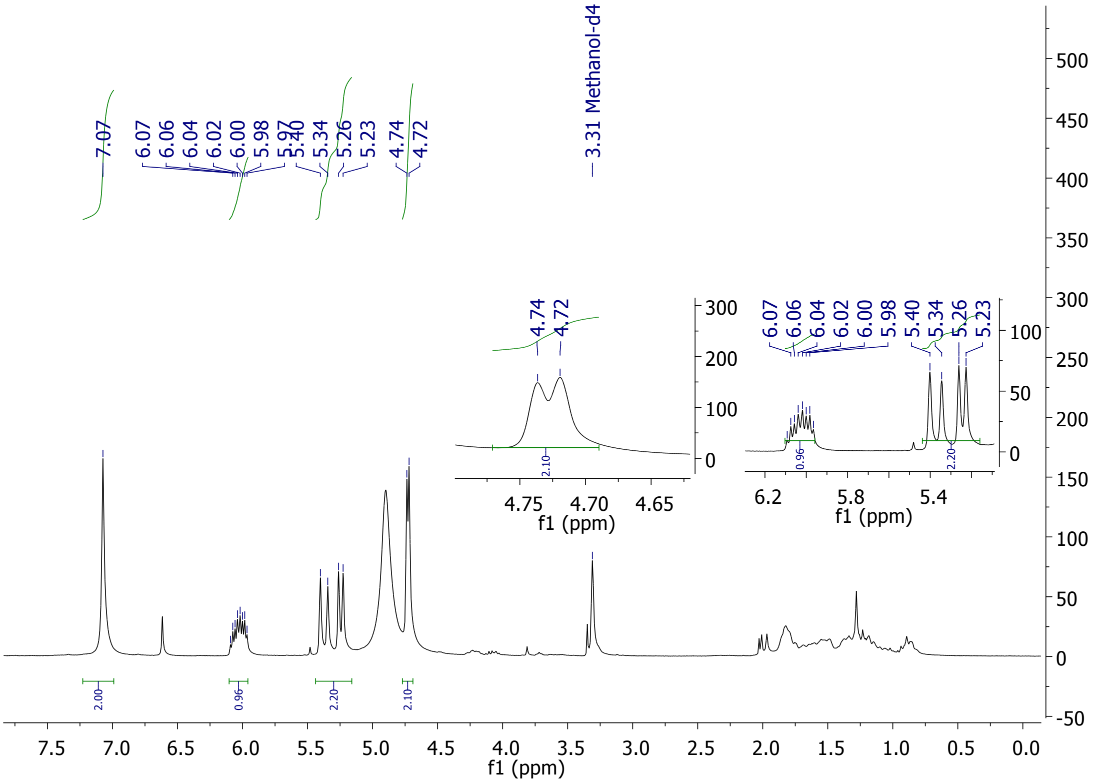


Figure S24: $^{1}$H-NMR (300 MHz, $CD_3OD$) spectrum of the compound **Allyl Gallate**

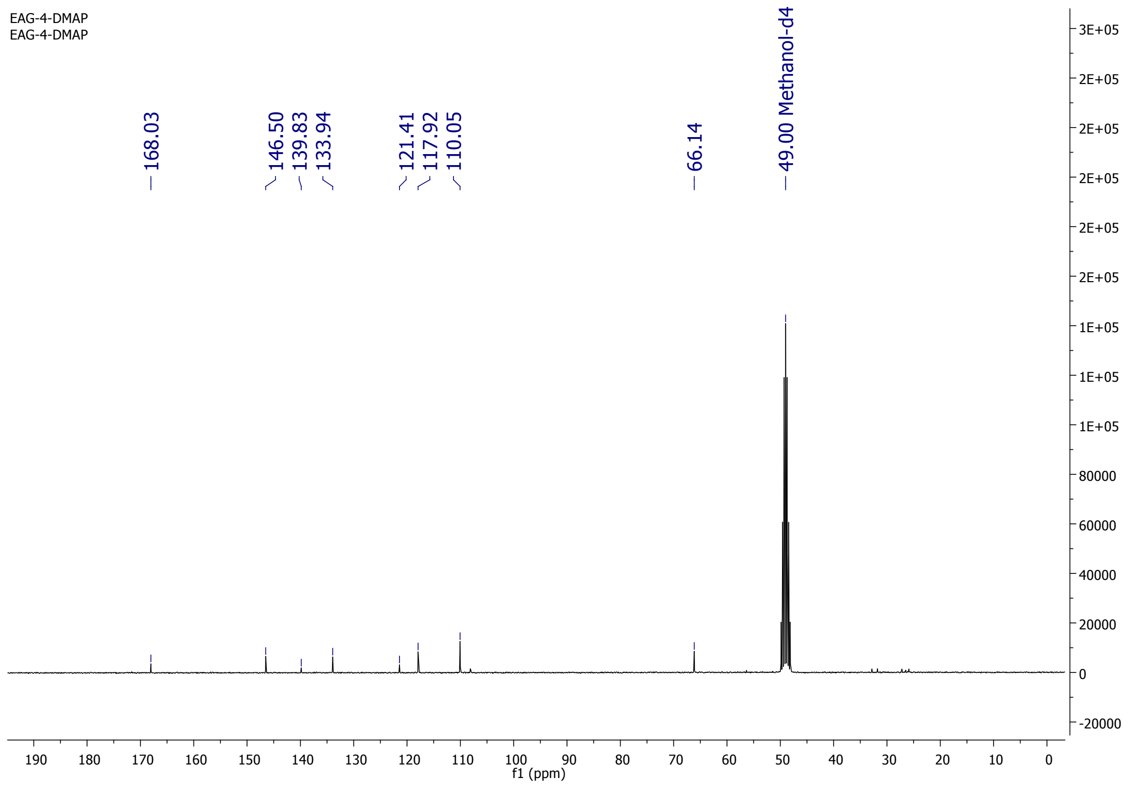


Figure S25: $^{13}$C-NMR (75 MHz, $CD_3OD$) spectrum of the compound **Allyl Gallate**

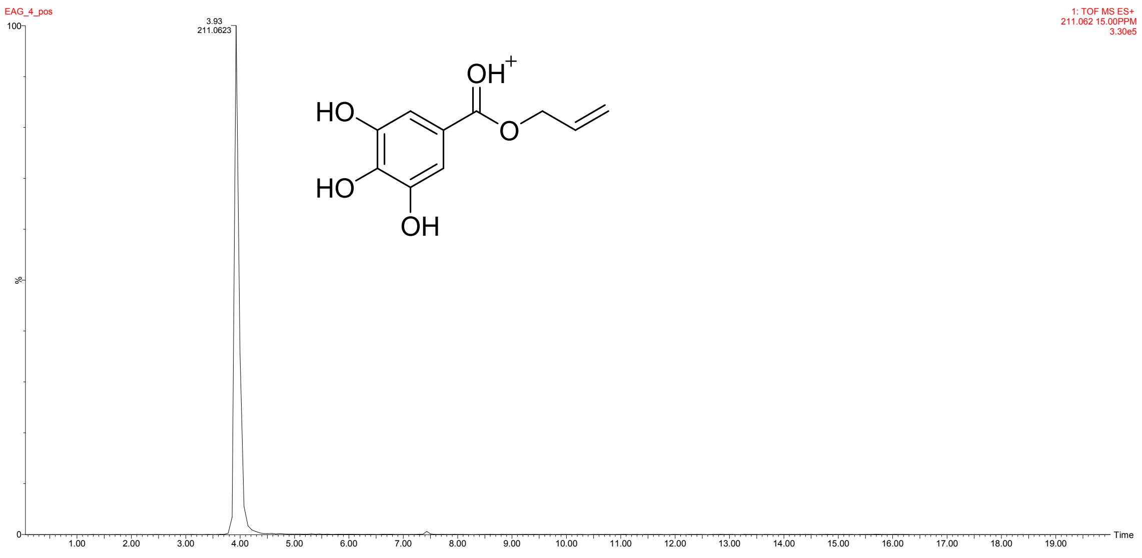


Figure S26: UPLC-ESI-MS of the compound **Allyl Gallate**

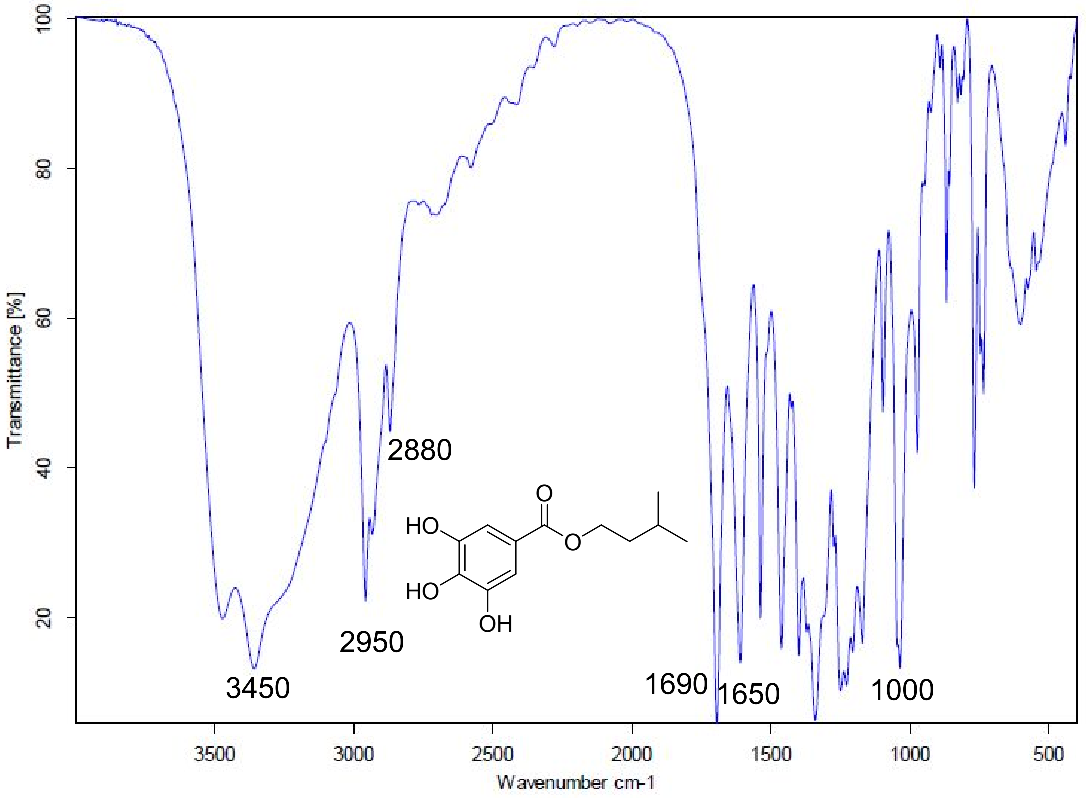


Figure S27: IR spectrum of the compound **Isoamyl Gallate**

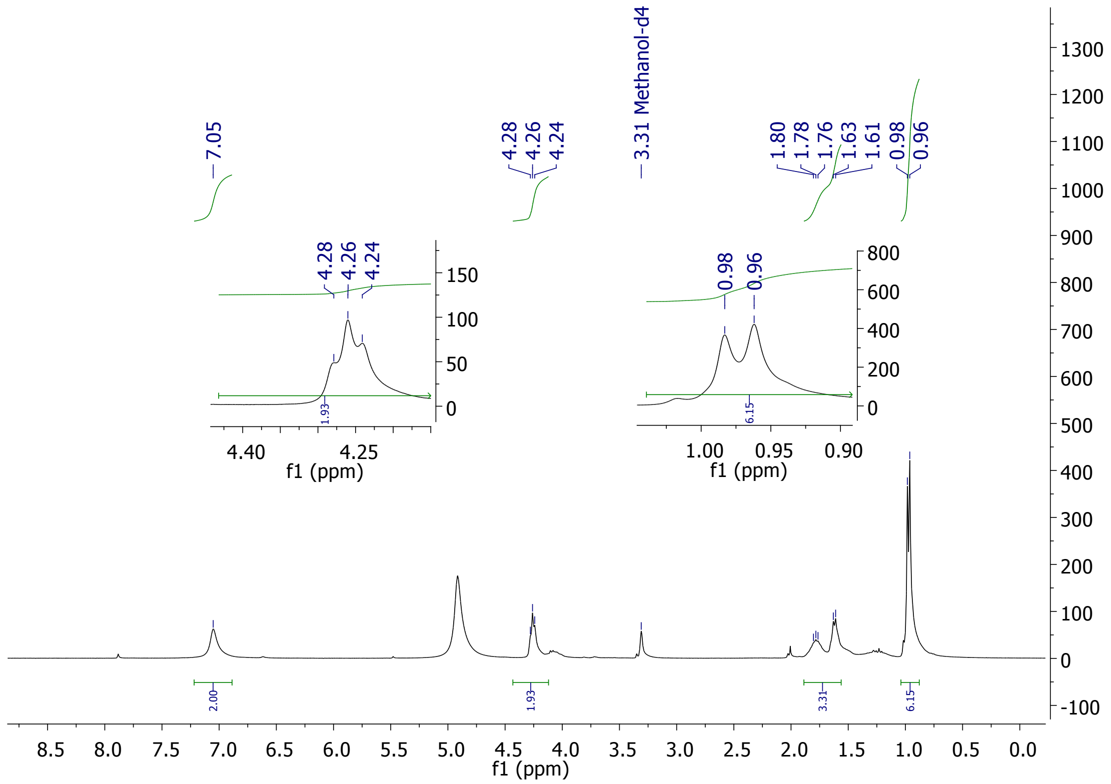


Figure S28: $^{1}$H-NMR (300 MHz, $CD_3OD$) spectrum of the compound **Isoamyl Gallate**

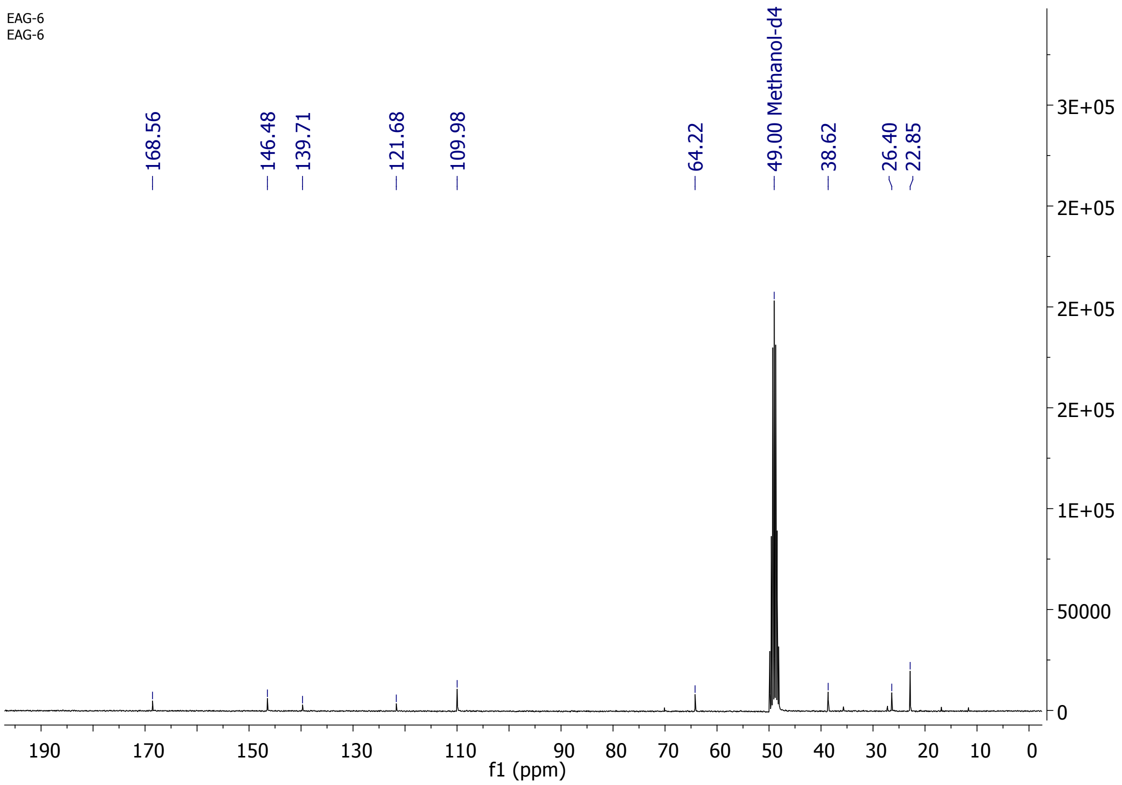


Figure S29: $^{13}$C-NMR (75 MHz, $CD_3OD$) spectrum of the compound **Isoamyl Gallate**

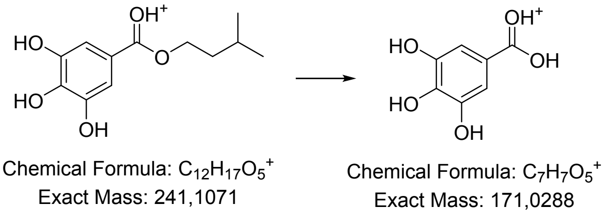


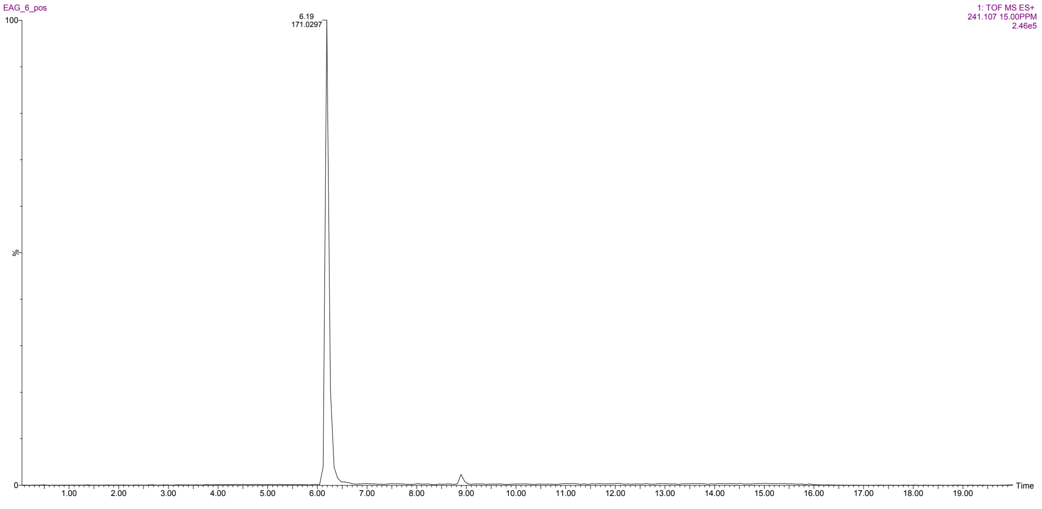


Figure S30: UPLC-ESI-MS of the compound **Isoamyl Gallate**

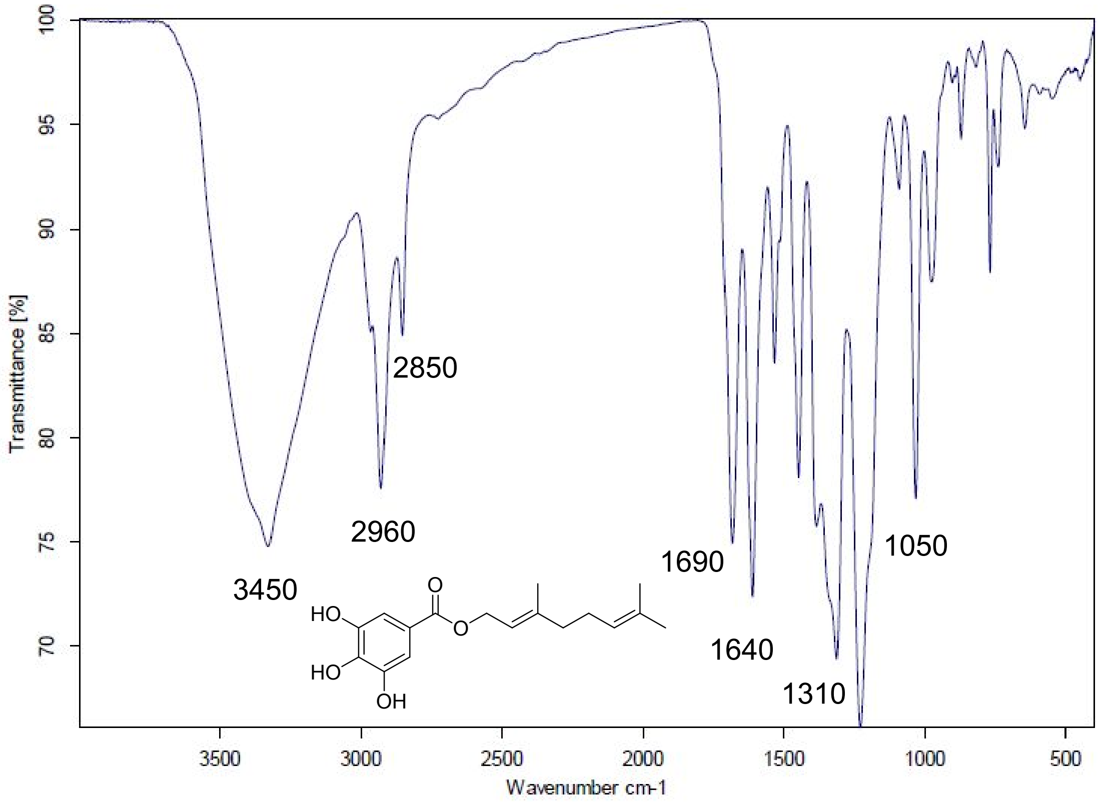


Figure S31: IR spectrum of the compound **Geranyl Gallate**

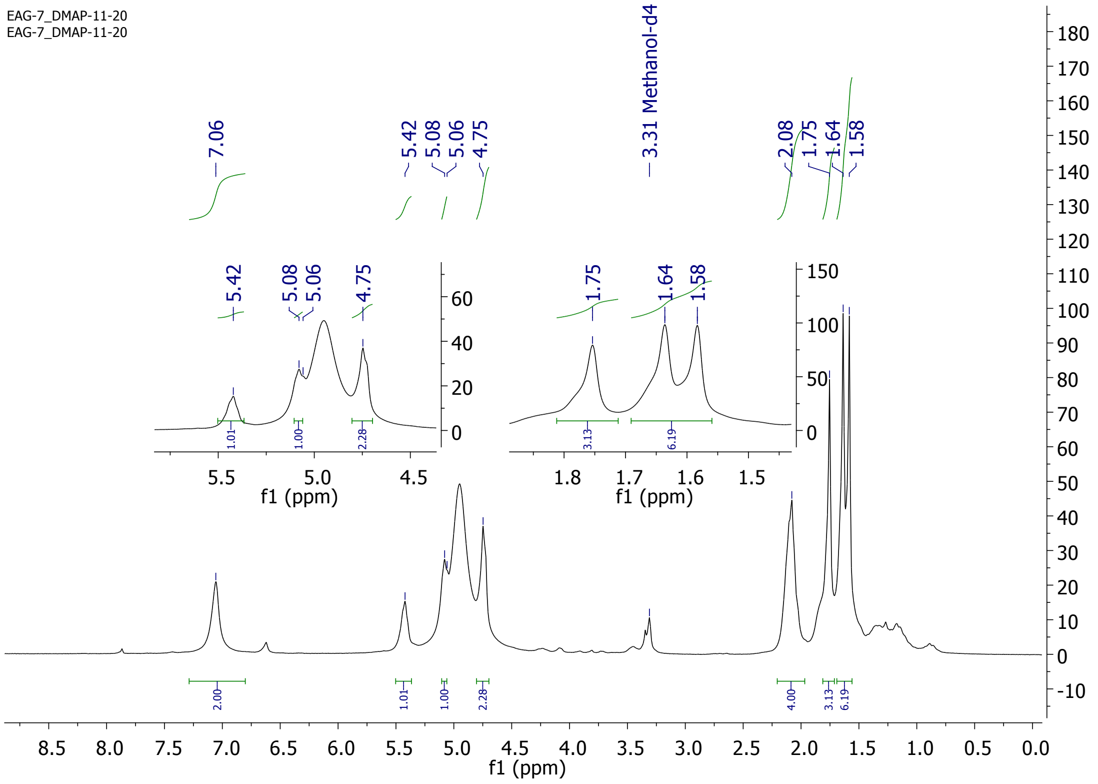


Figure S32: $^{1}$H-NMR (300 MHz, $CD_3OD$) spectrum of the compound **Geranyl Gallate**

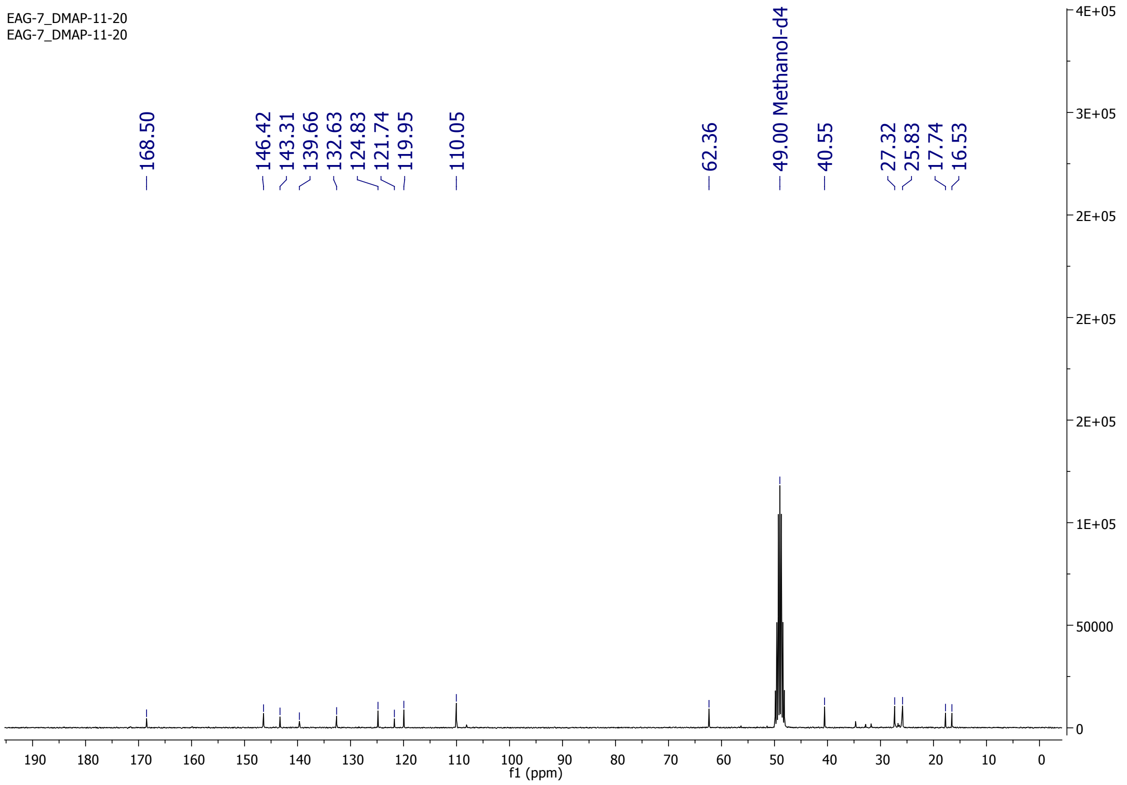


Figure S33: $^{13}C$-NMR (75 MHz, $CD_3OD$) spectrum of the compound **Geranyl Gallate**

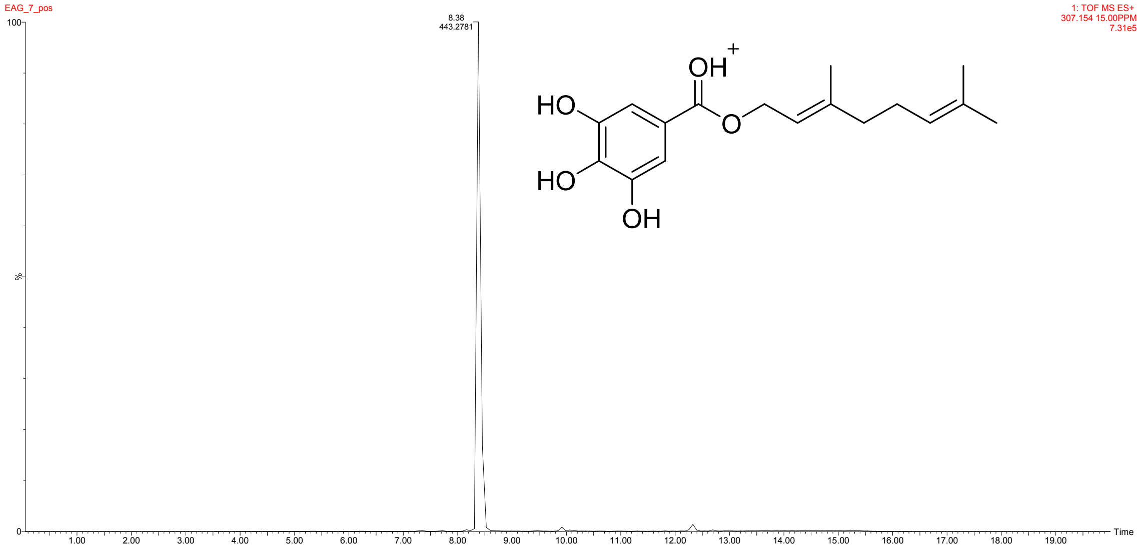


Figure S34: UPLC-ESI-MS of the compound **Geranyl Gallate**

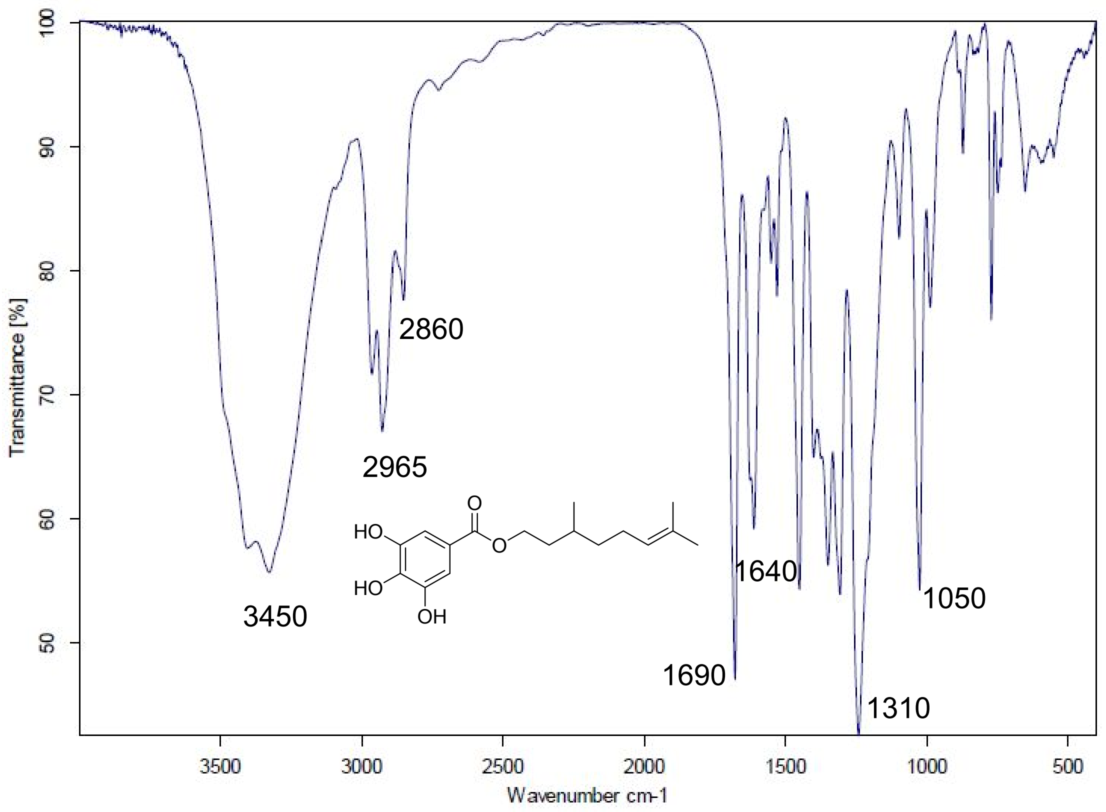


Figure S35: IR spectrum of the compound **Citronellyl gallate**

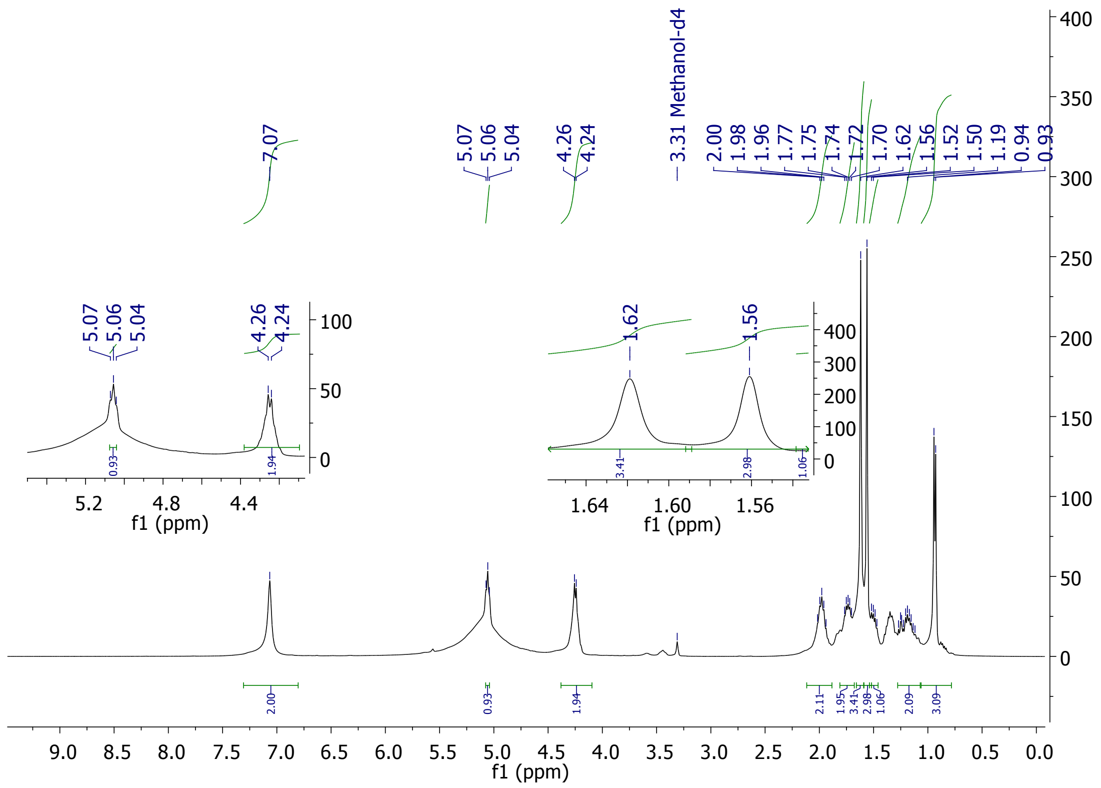


Figure S36: $^{1}$H-NMR (400 MHz, $CD_3OD$) spectrum of the compound **Citronellyl gallate**

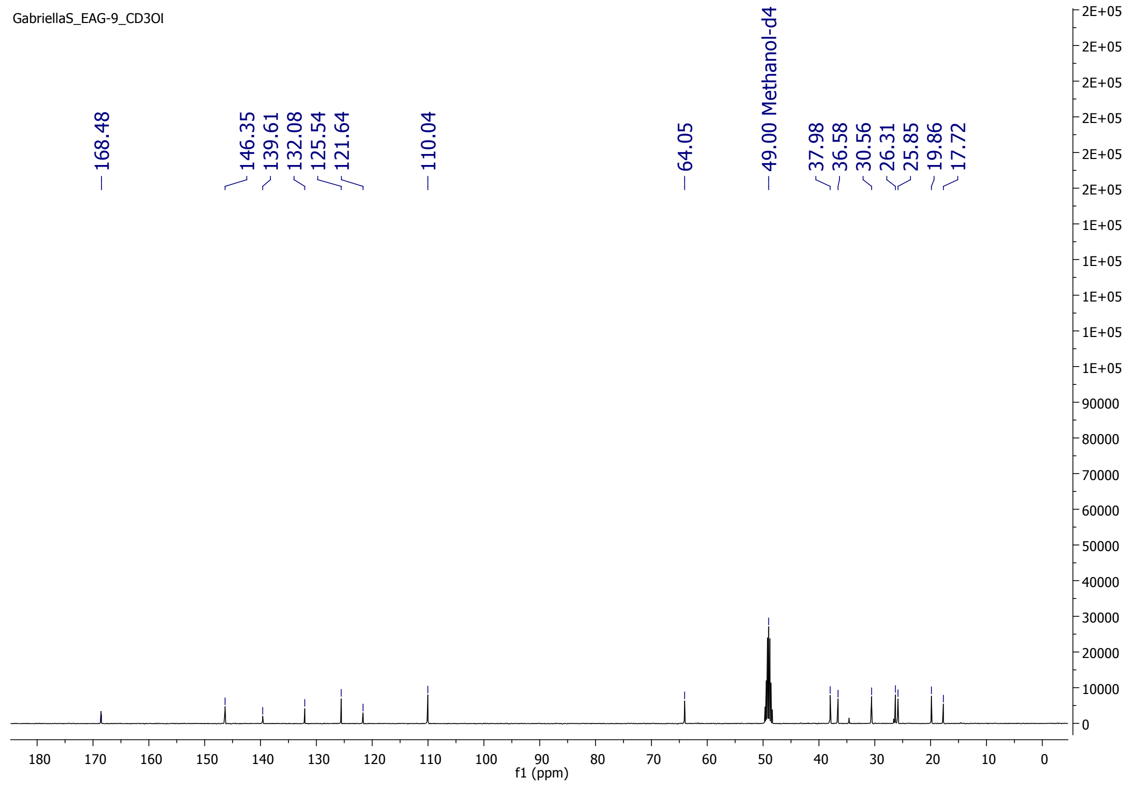


Figure S37: $^{13}$C-NMR (100 MHz, $CD_3OD$) spectrum of the compound **Citronellyl gallate**

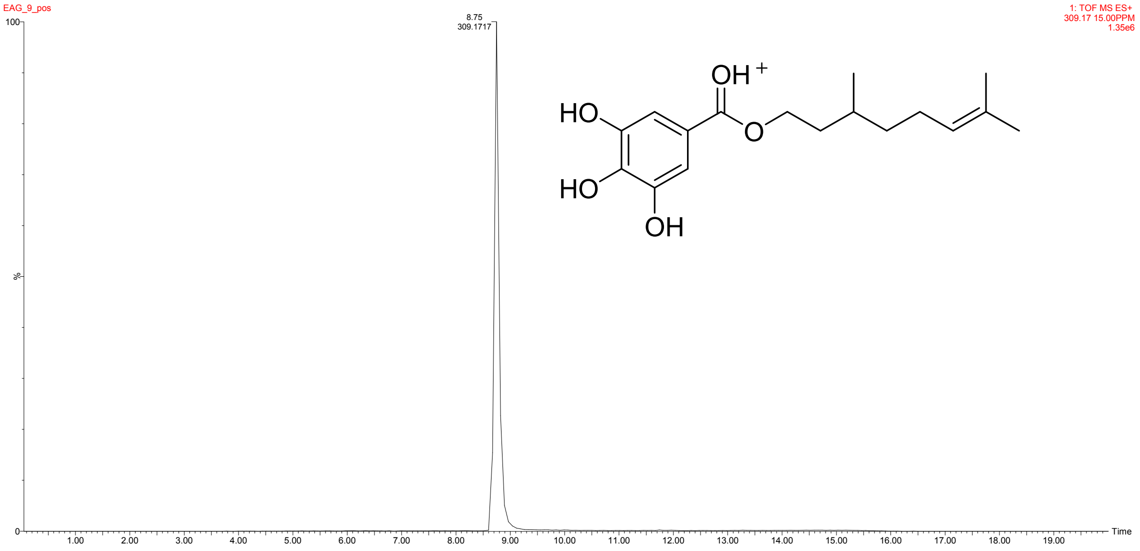


Figure S38: UPLC-ESI-MS of the compound **Citronellyl gallate**

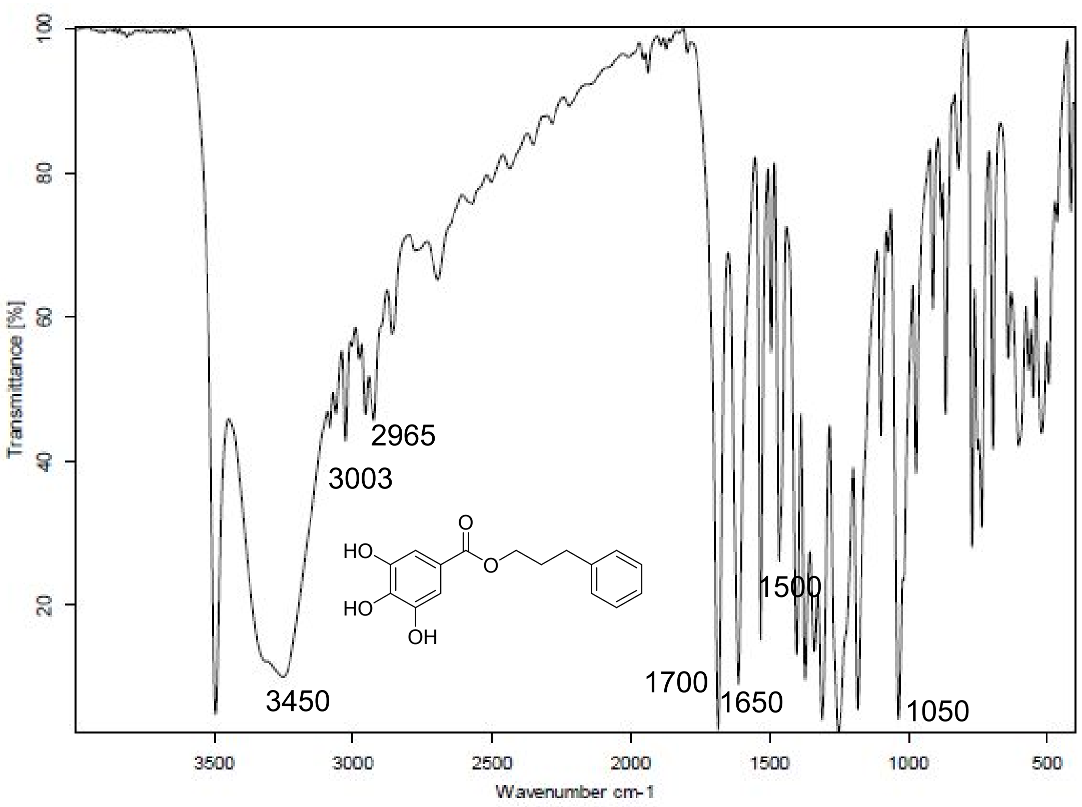


Figure S39: IR spectrum of the compound **3-Phenyl propyl gallate**

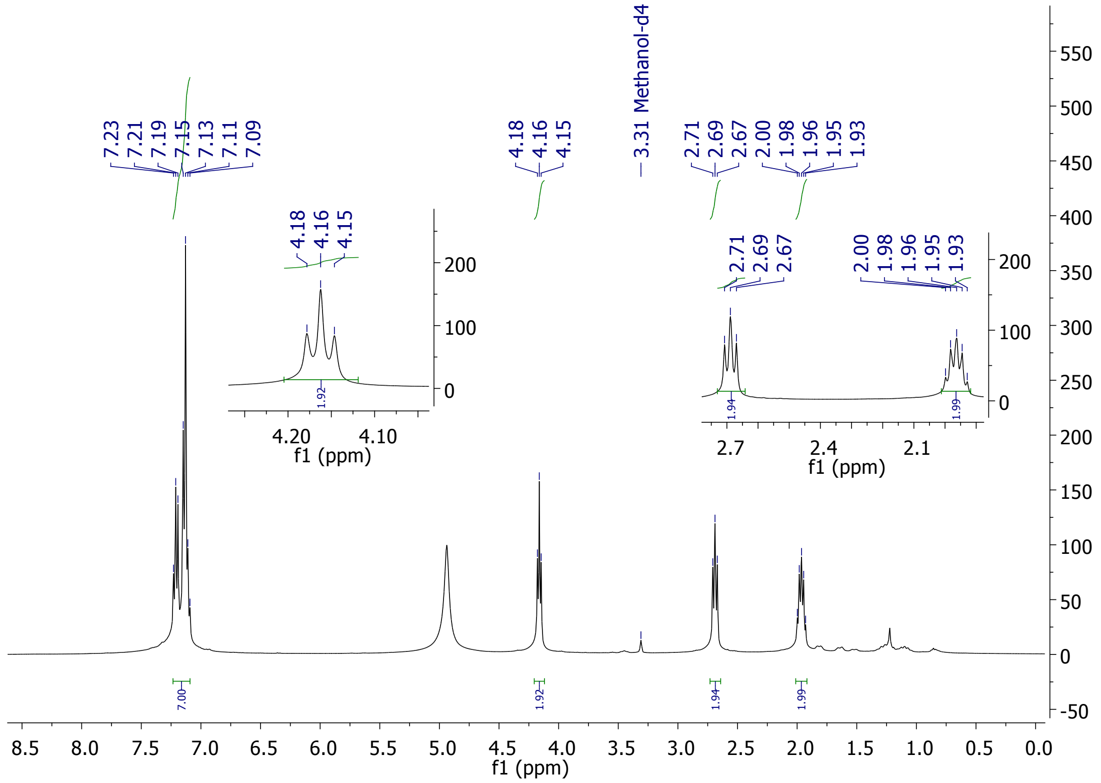


Figure S40: $^{1}$H-NMR (400 MHz, $CD_3OD$) spectrum of the compound **3-Phenyl propyl gallate.**

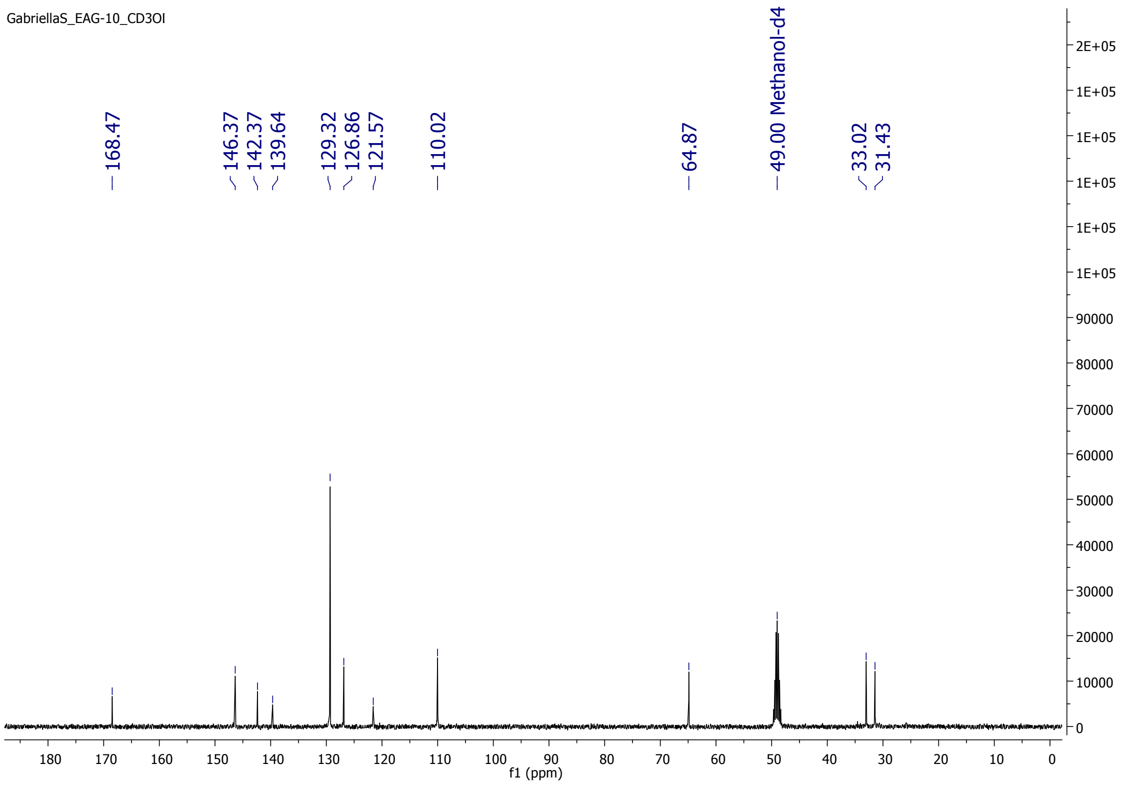


Figure S41: $^{13}$C-NMR (100 MHz, $CD_3OD$) spectrum of the compound **3-Phenyl propyl gallate.**

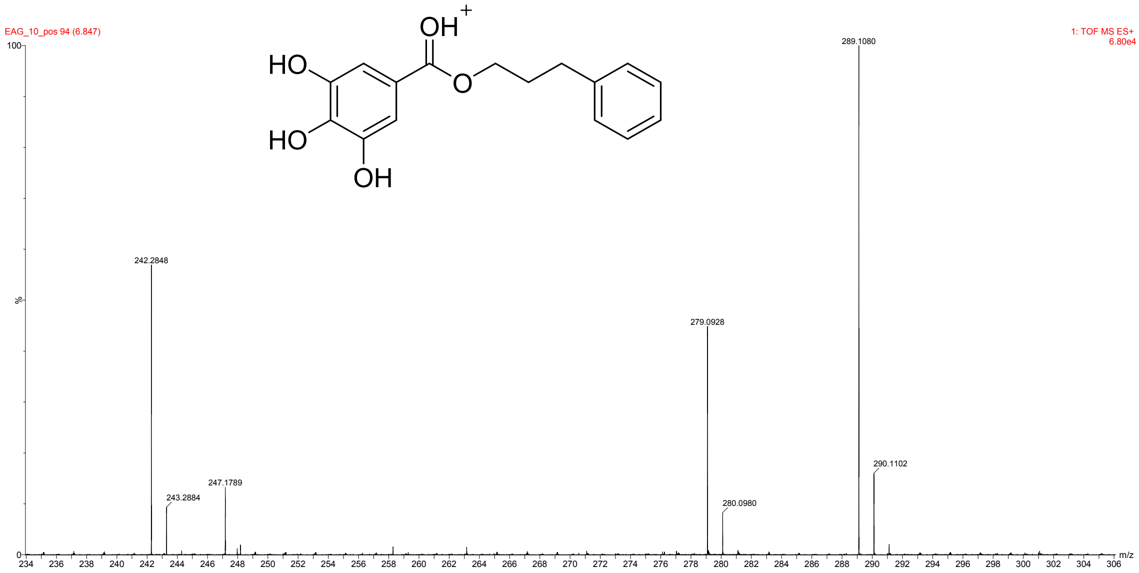


Figure S42: UPLC-ESI-MS of the compound **3-Phenyl propyl gallate.**

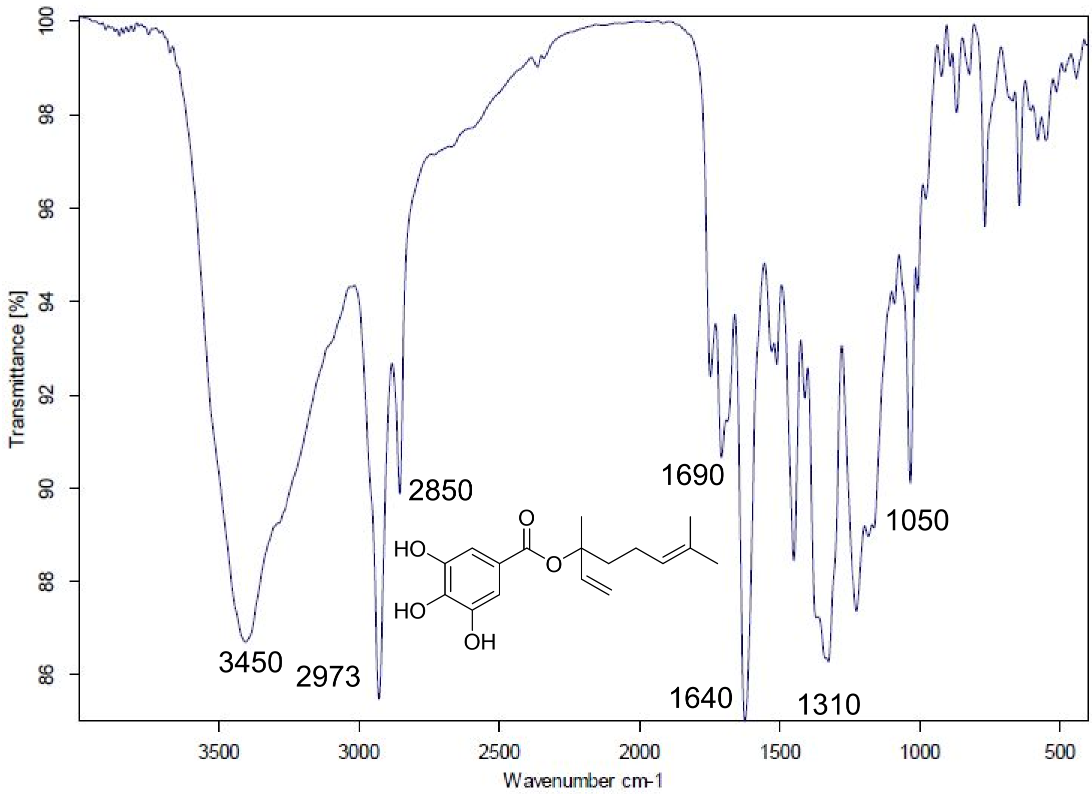


Figure S43: IR spectrum of the compound **Linalyl gallate**

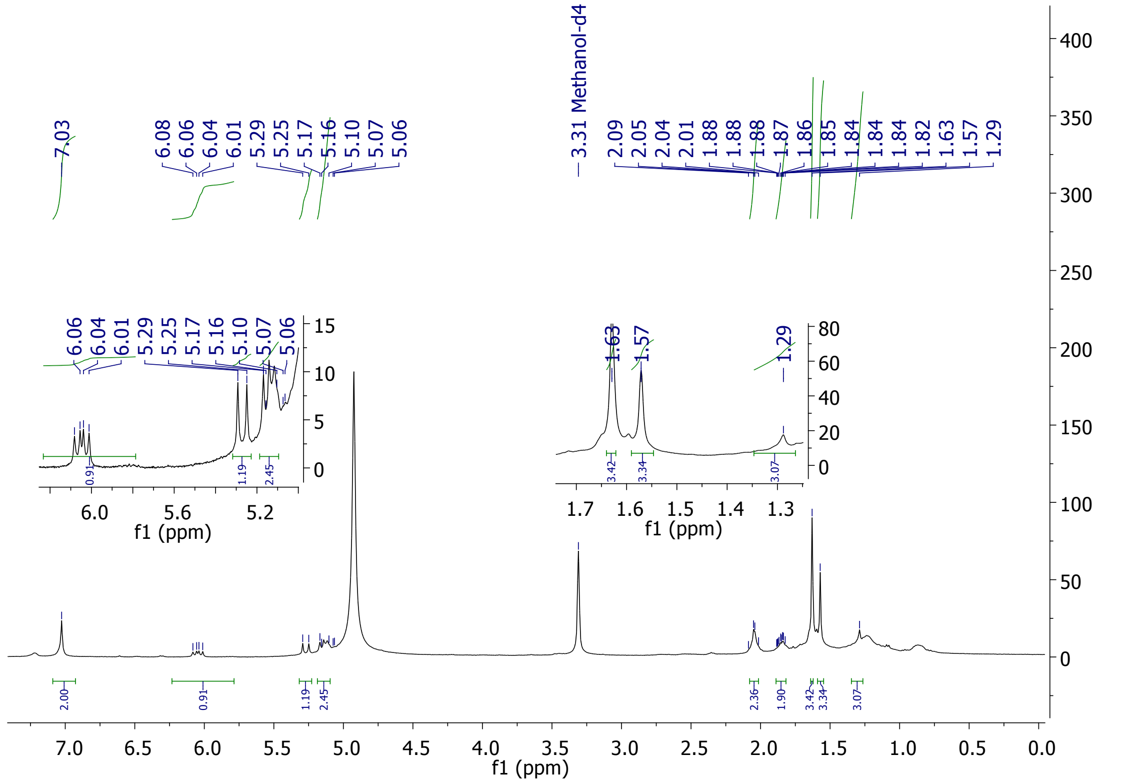


Figure S44: $^{1}$H-NMR (400 MHz, $CD_3OD$) spectrum of the compound **Linalyl gallate**

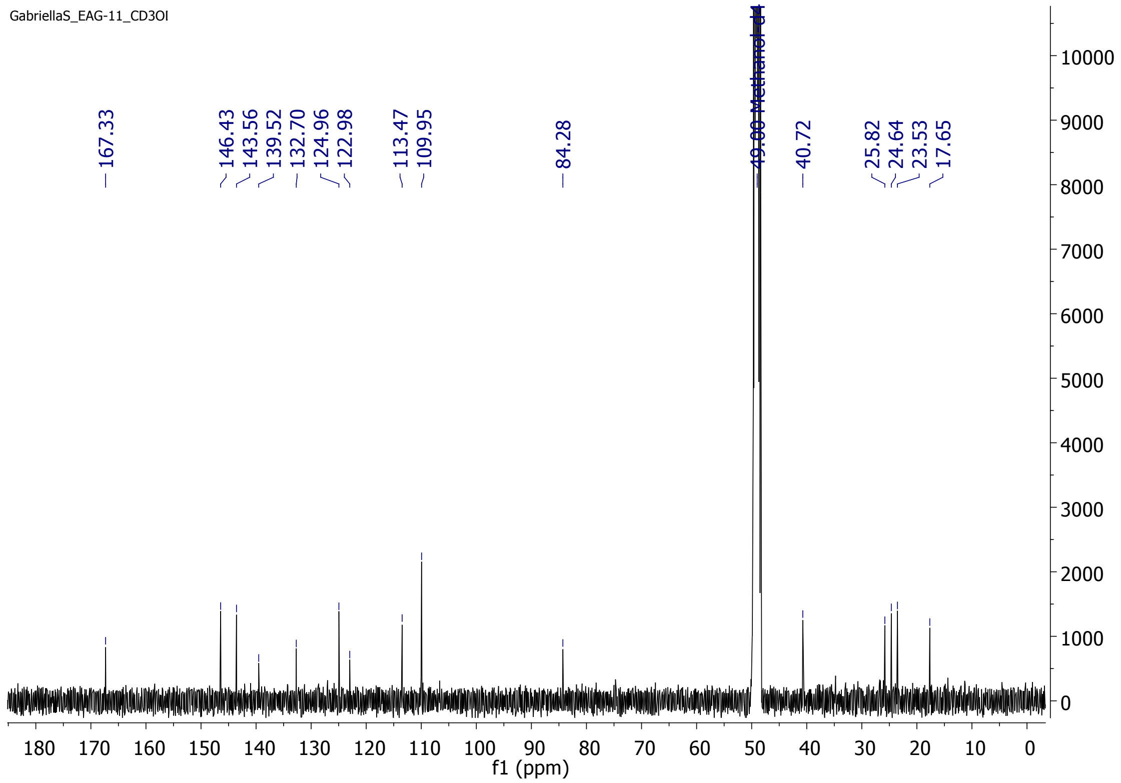


Figure S45: $^{13}C$-NMR (100 MHz, $CD_3OD$) spectrum of the compound **Linalyl gallate**

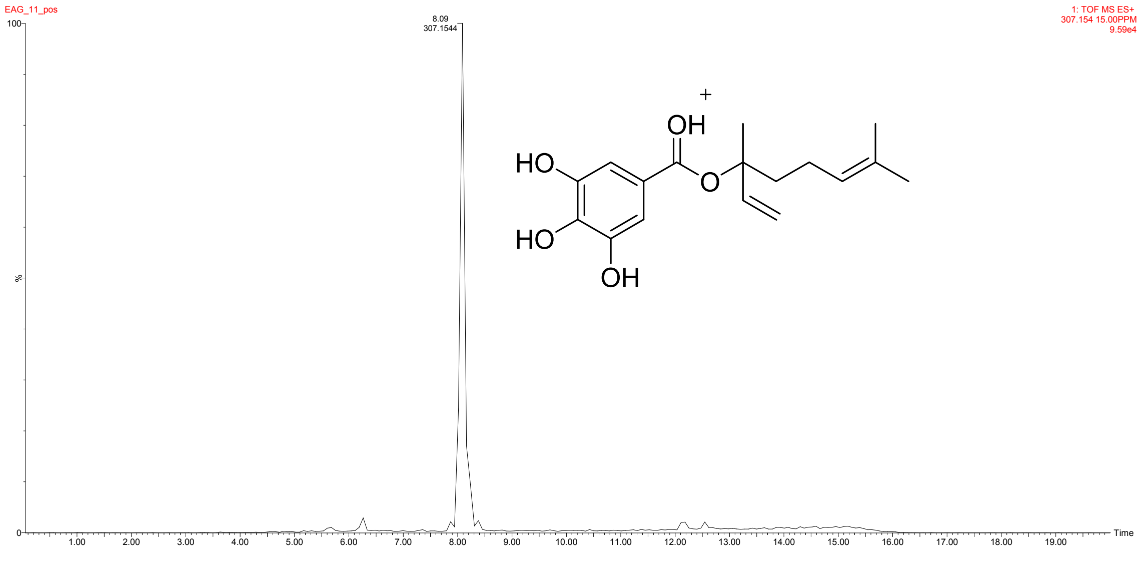


Figure S46: UPLC-ESI-MS of the compound **Linalyl gallate**

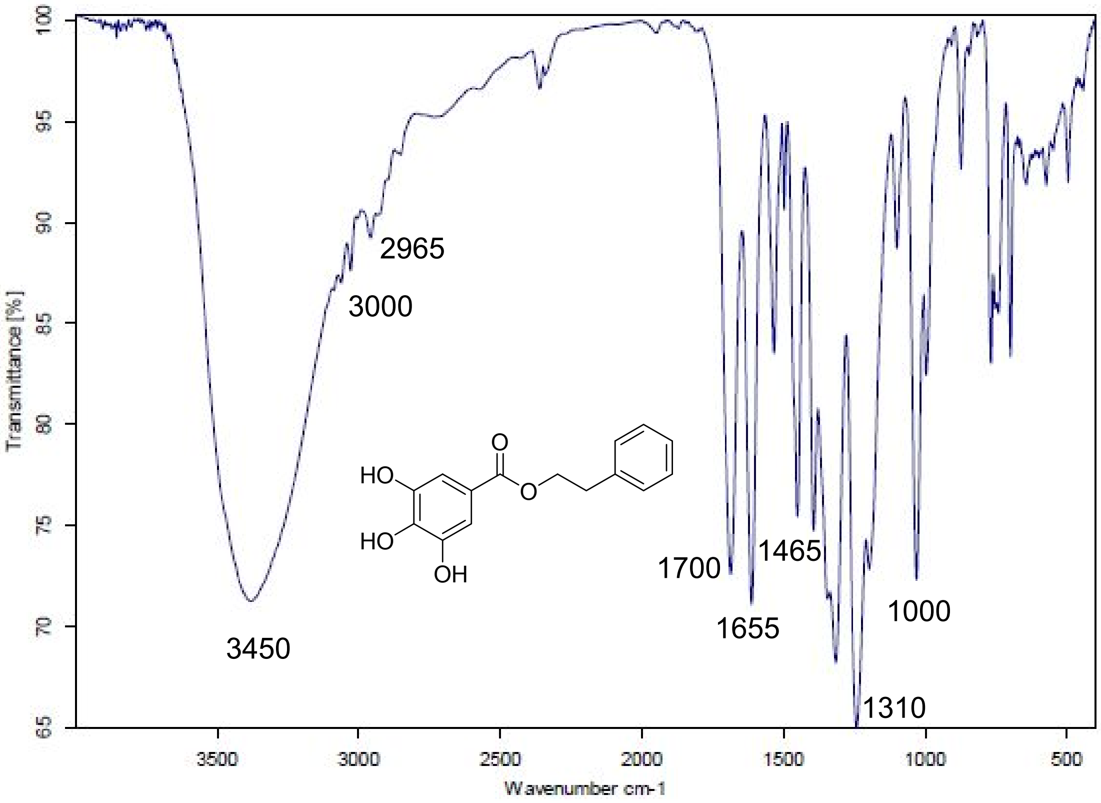


Figure S47: IR spectrum of the compound **2-Phenyl ethyl gallate**

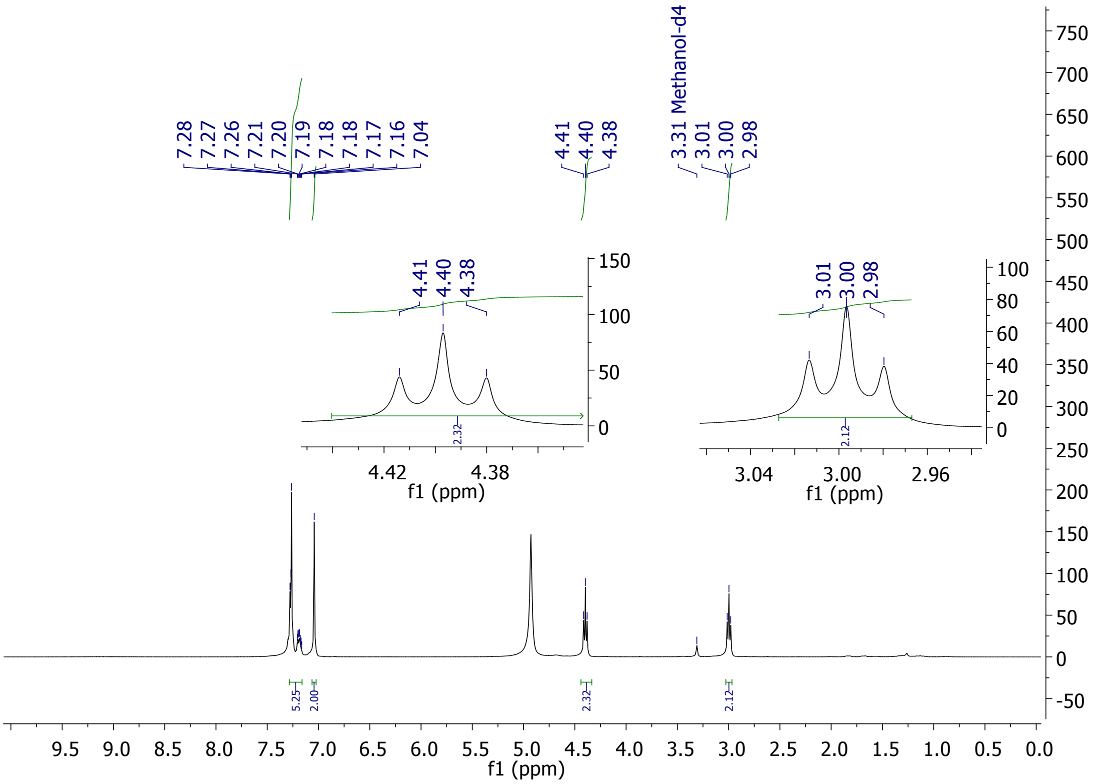


Figure S48: $^{1}$H-NMR (400 MHz, $CD_3OD$) spectrum of the compound **2-Phenyl ethyl gallate**

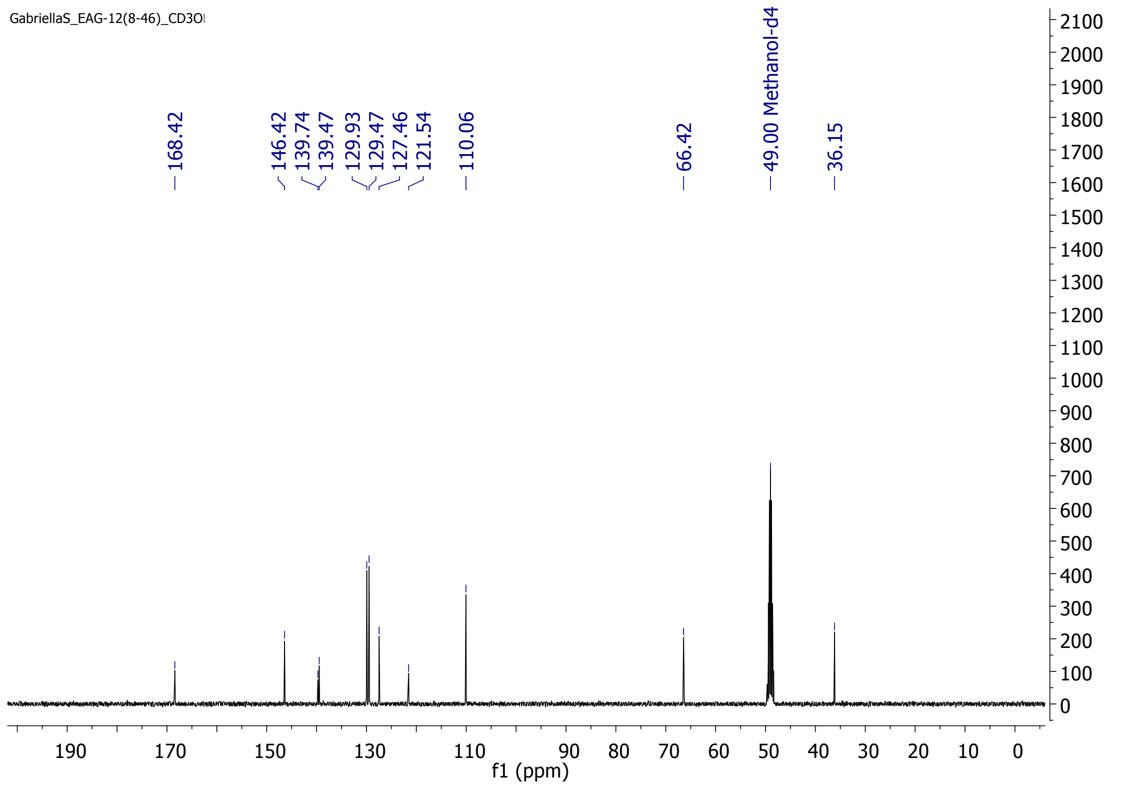


Figure S49: $^{13}$C-NMR (100 MHz, $CD_3OD$) spectrum of the compound **2-Phenyl ethyl gallate**

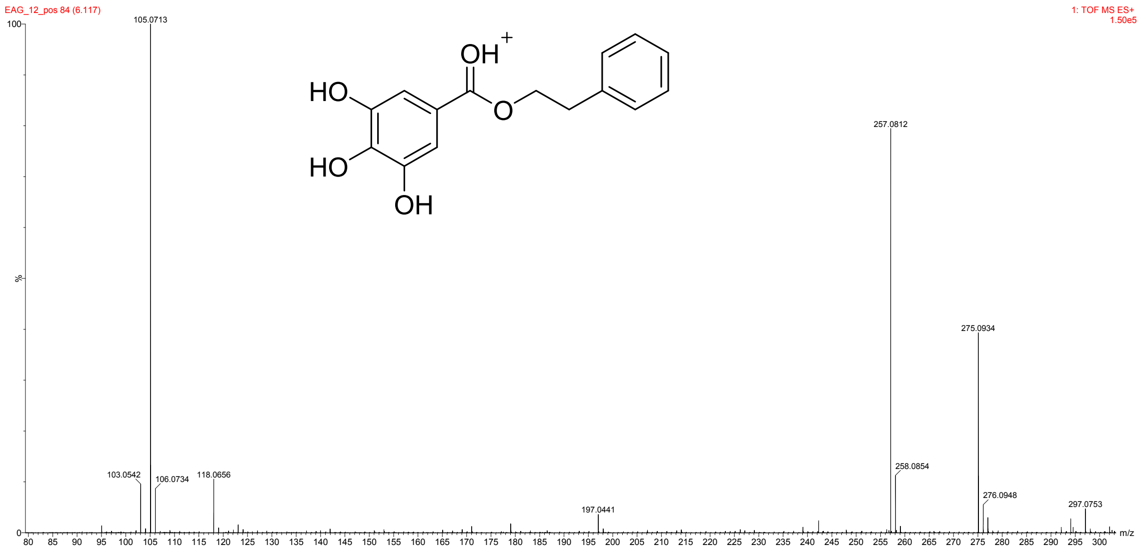


Figure S50: UPLC-ESI-MS of the compound **2-Phenyl ethyl gallate**

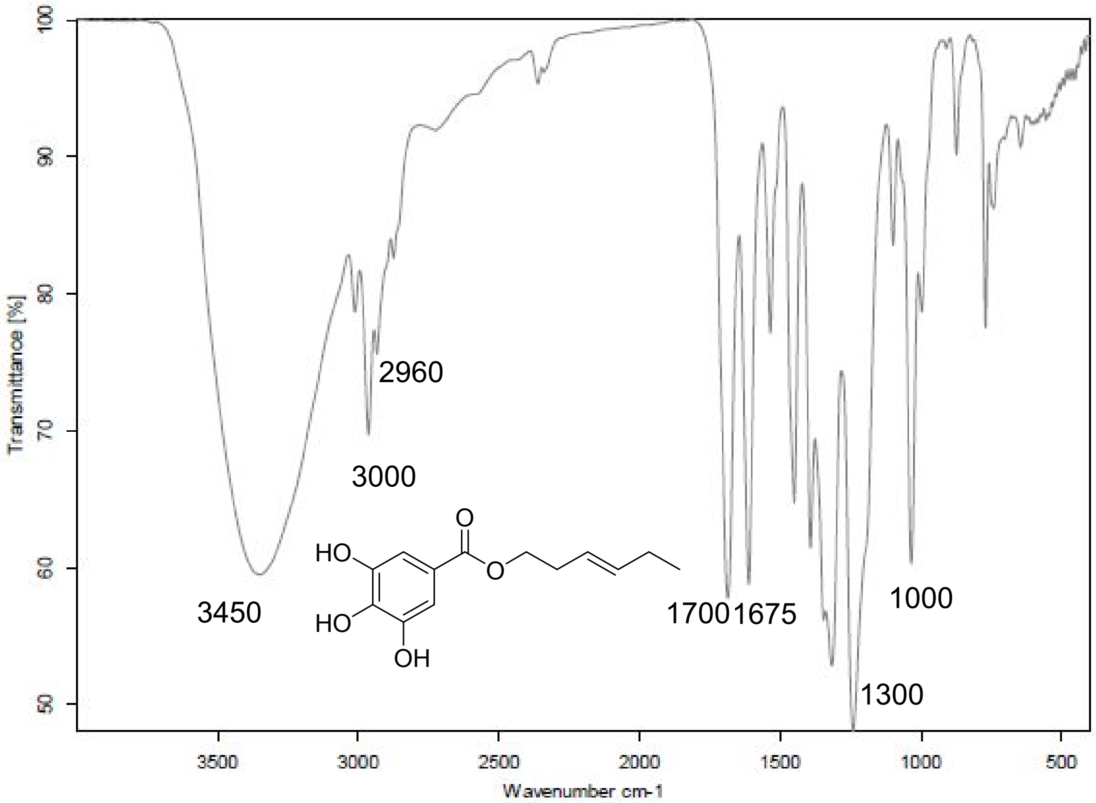


Figure S51: IR spectrum of the compound **Hex-3-enyl gallate**

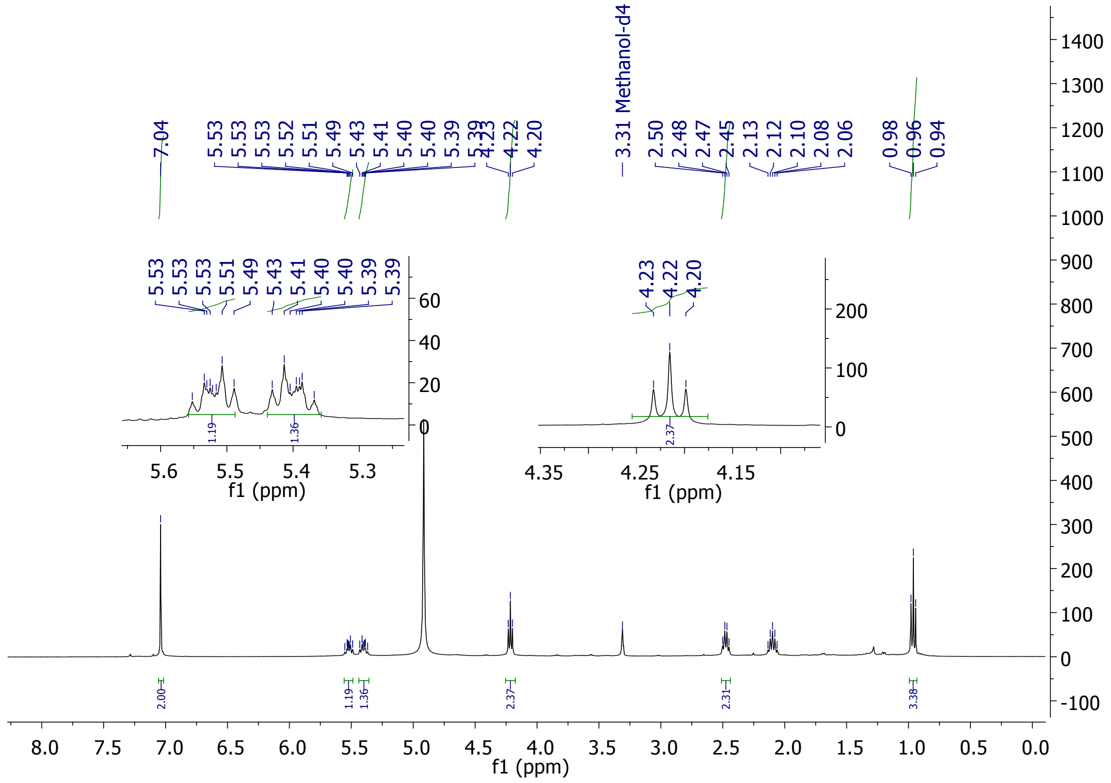


Figure S52: $^{1}$H-NMR (400 MHz, $CD_3OD$) spectrum of the compound **Hex-3-enyl gallate**

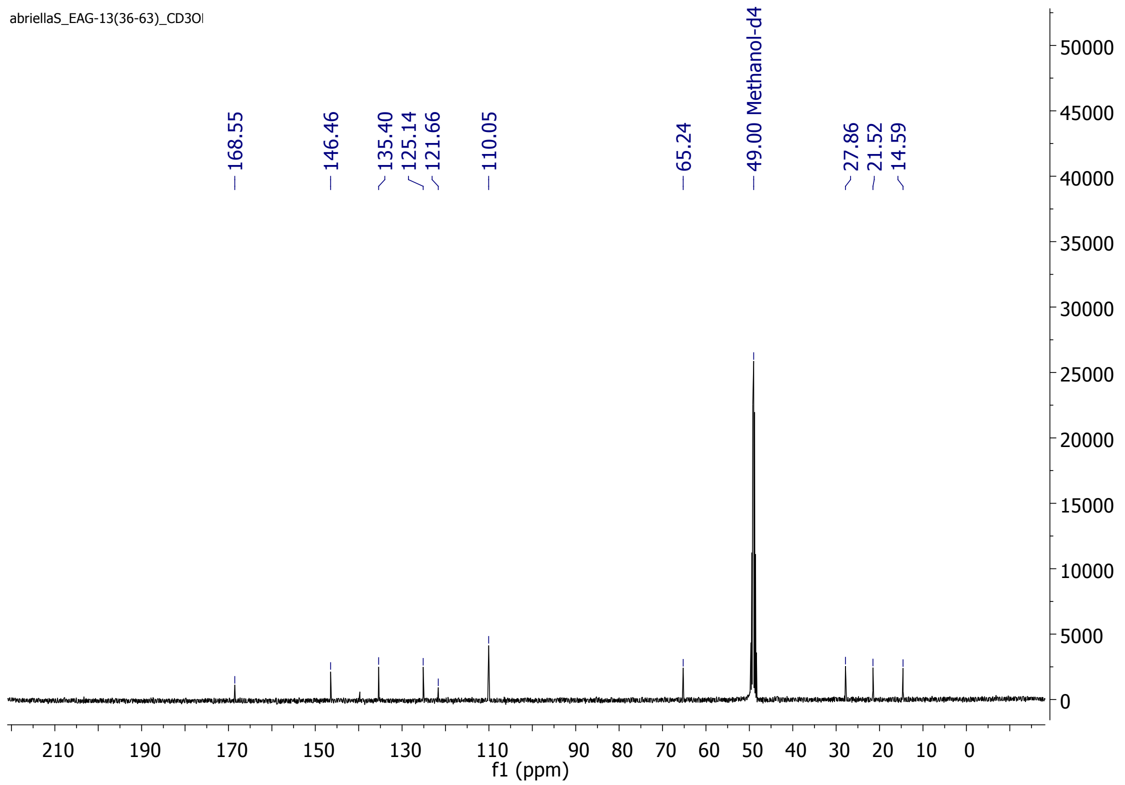


Figure S53: $^{13}C$-NMR (100 MHz, $CD_3OD$) spectrum of the compound **Hex-3-enyl gallate**

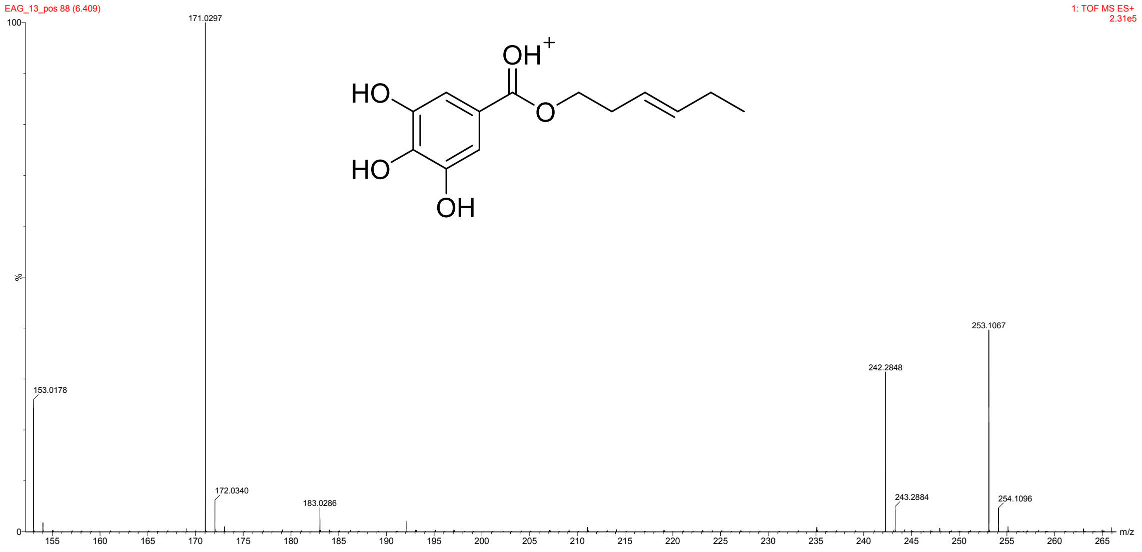


Figure S54: UPLC-ESI-MS of the compound **Hex-3-enyl gallate**

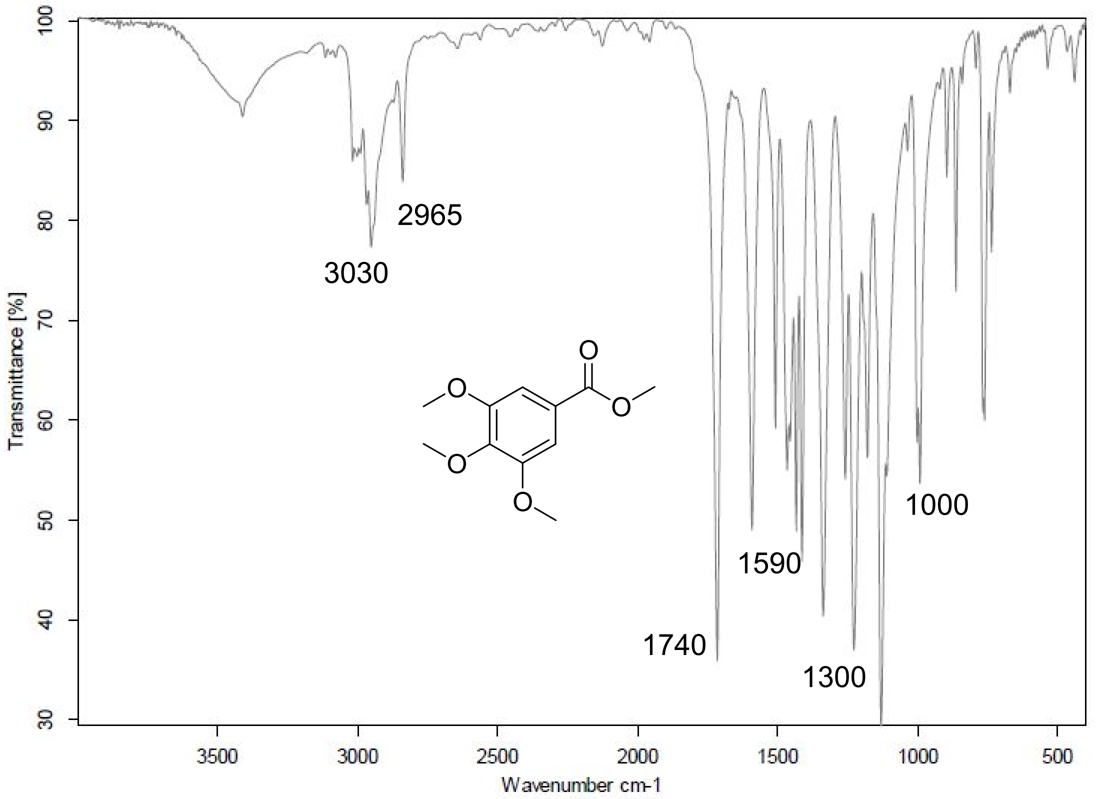


Figure S55: IR spectrum of the compound **Benzoic acid, 3,4,5-trimethoxy-methyl ester**

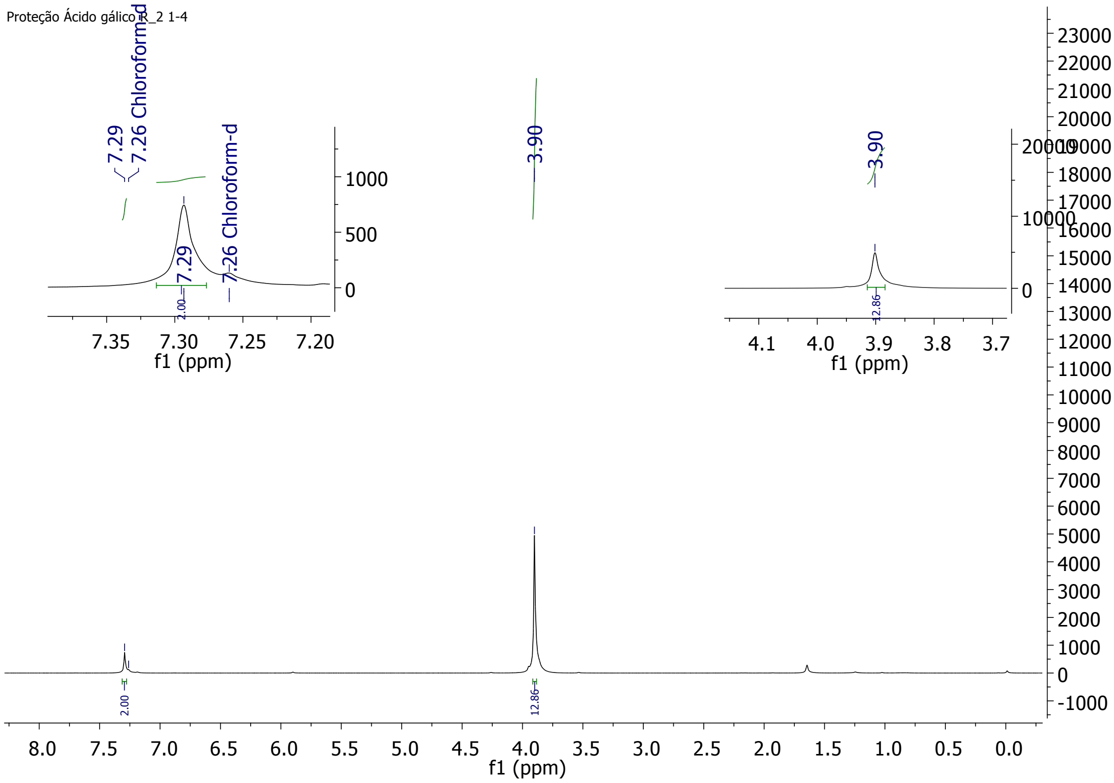


Figure S56: $^1$H-NMR (200 MHz, $CDCl_3$) spectrum of the compound compound **Benzoic acid, 3,4,5-trimethoxy-methyl ester**

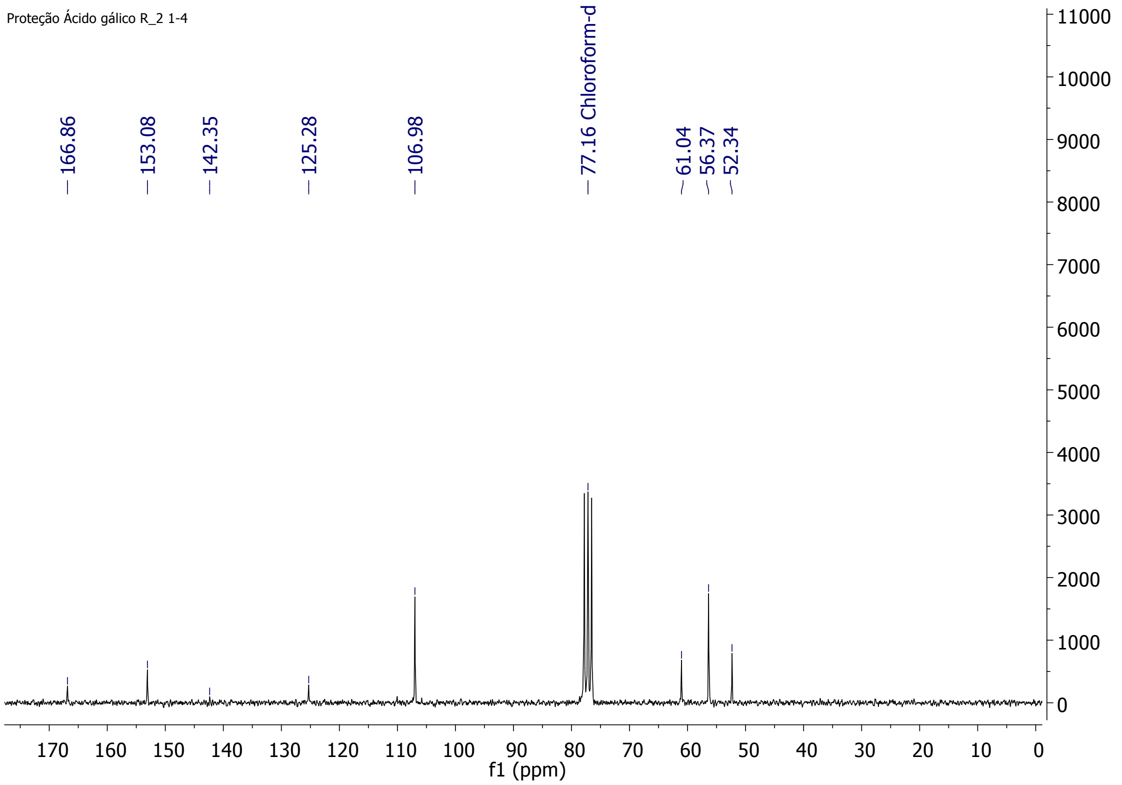


Figure S57: $^{1}$H-NMR (50 MHz, $CDCl_3$) spectrum of the compound compound **Benzoic acid, 3,4,5-trimethoxy-methyl ester.**

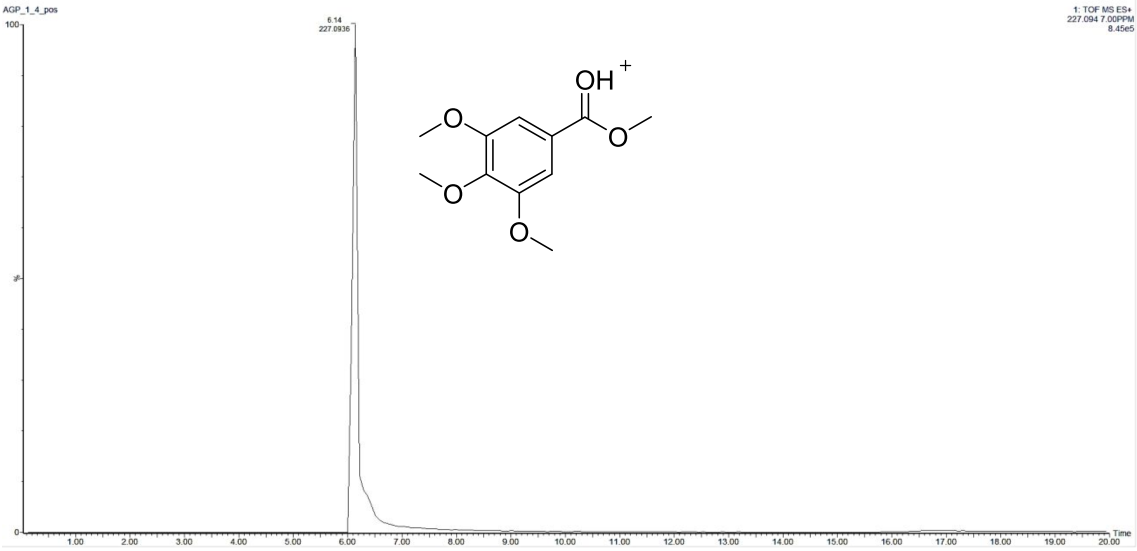


Figure S58: UPLC-ESI-MS of the compound **Benzoic acid, 3,4,5-trimethoxy-methyl ester.**

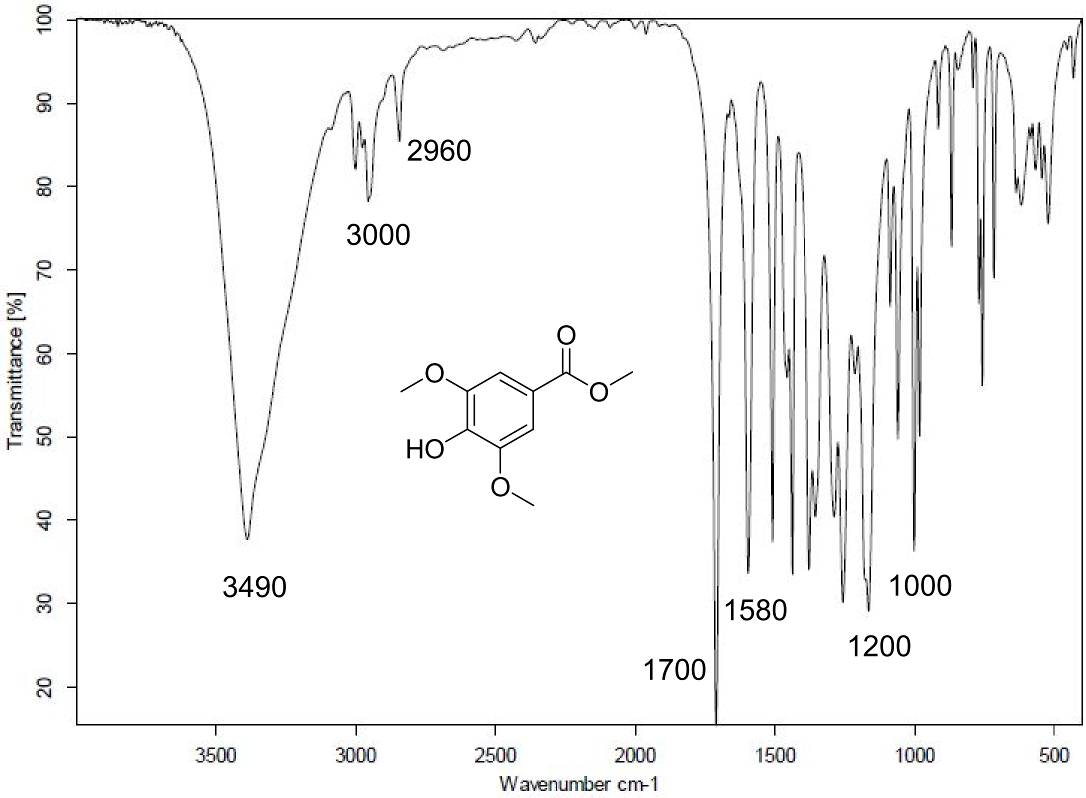


Figure S59: IR spectrum of the compound **4-hydroxy-3,5-dimethoxy-methyl ester**

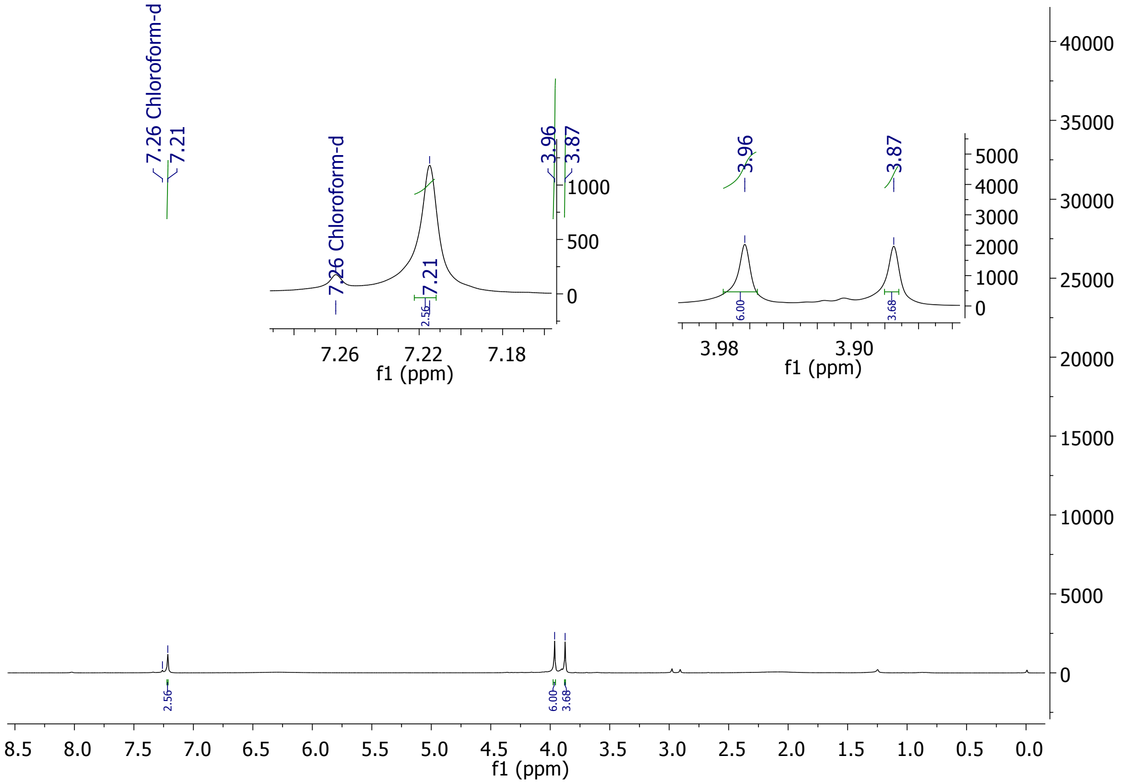


Figure S60: $^{1}$H-NMR (200 MHz, $CDCl_3$) spectrum of the compound **4-hydroxy-3,5-dimethoxy-methyl ester**

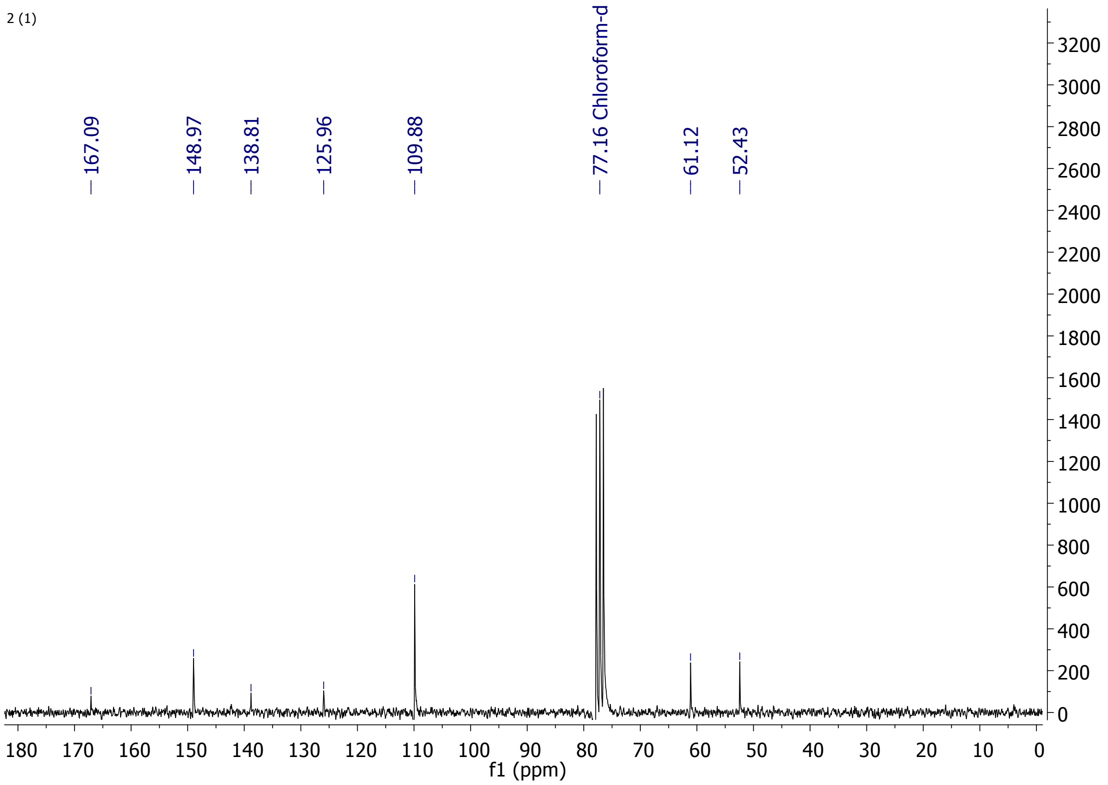


Figure S61: $^{13}$C-NMR (50 MHz, $CDCl_3$) spectrum of the compound **4-hydroxy-3,5-dimethoxy-methyl ester**

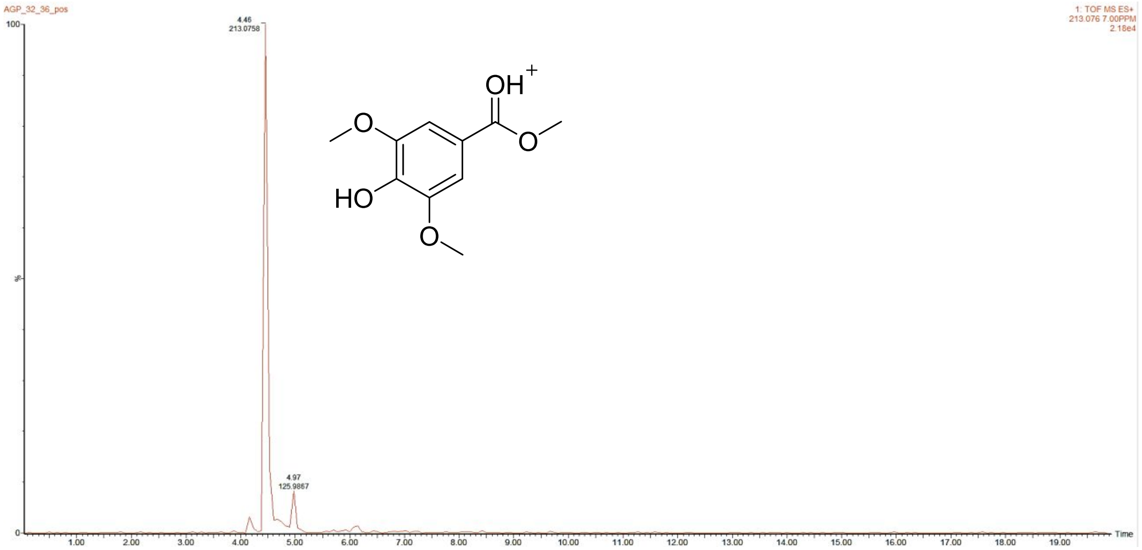


Figure S62: UPLC-ESI-MS of the compound **4-hydroxy-3,5-dimethoxy-methyl ester.**
